# A pan-*Zea* genome map for enhancing maize improvement

**DOI:** 10.1186/s13059-022-02742-7

**Published:** 2022-08-23

**Authors:** Songtao Gui, Wenjie Wei, Chenglin Jiang, Jingyun Luo, Lu Chen, Shenshen Wu, Wenqiang Li, Yuebin Wang, Shuyan Li, Ning Yang, Qing Li, Alisdair R. Fernie, Jianbing Yan

**Affiliations:** 1 National Key Laboratory of Crop Genetic Improvement, Huazhong Agricultural University, Wuhan, 430070 China; 2 Hubei Hongshan Laboratory, Wuhan, 430070 China; 3 Department of Molecular Physiology, Max-Planck-Institute of Molecular Plant Physiology, Am Mühlenberg 1, 14476 Potsdam, Golm, Germany

**Keywords:** Pan-*Zea* genome, Gene presence/absence variation (gPAV), Structural variation (SV), Narrow-sense heritability, GWAS

## Abstract

**Background:**

Maize (*Zea mays* L.) is at the vanguard facing the upcoming breeding challenges. However, both a super pan-genome for the *Zea* genus and a comprehensive genetic variation map for maize breeding are still lacking.

**Results:**

Here, we construct an approximately 6.71-Gb pan-*Zea* genome that contains around 4.57-Gb non-B73 reference sequences from fragmented de novo assemblies of 721 pan-*Zea* individuals. We annotate a total of 58,944 pan-*Zea* genes and find around 44.34% of them are dispensable in the pan-*Zea* population. Moreover, 255,821 common structural variations are identified and genotyped in a maize association mapping panel. Further analyses reveal gene presence/absence variants and their potential roles during domestication of maize. Combining genetic analyses with multi-omics data, we demonstrate how structural variants are associated with complex agronomic traits.

**Conclusions:**

Our results highlight the underexplored role of the pan-*Zea* genome and structural variations to further understand domestication of maize and explore their potential utilization in crop improvement.

**Supplementary Information:**

The online version contains supplementary material available at 10.1186/s13059-022-02742-7.

## Background

The increasing population and unpredictability evoked by global climate change have brought new demands to increase the productivity and quality of our crops [1]. Food production must increase 70% by 2050 to feed the increase in the world’s population [2]. The past few decades have witnessed a rapid evolution of sequencing and marker technologies alongside the widespread adoption of genome-based breeding approaches [3]. These technological revolutions have promoted innovations in crop breeding from conventional phenotype-based selection to genomics-assisted breeding and genetic engineering [4, 5].

While they harbor great potential, the development of breeding technologies and the explosive growth of biological information have also highlighted the insufficiencies in conventional genomics-assisted breeding strategies. The first of these insufficiencies is the use of a single reference genome. More and more evidences have shown that mapping reads onto a single reference genome can result in reference bias and missing information in highly polymorphic regions and regions that are not present in the genome [6–8]. Thus, a more comprehensive way is to replace the single reference genome with a pan-genome, which represents the complete genetic repertoire of a species. With reduced sequencing costs in recent years, the desirability to construct pan-genomes has spread from *Streptococcus agalactiae* [9] to eukaryotic species [10–12], including many major crops, such as rice, bread wheat, soybean, and tomato [13–16]. Secondly, the conventional genomics-assisted breeding strategies majorly rely on single nucleotide polymorphisms (SNPs) and short insertions/deletions (InDels, hereafter representing insertions/deletions < 50 bp) because they could be easily acquired from low-depth resequencing of cultivated lines. However, SNPs/InDels do not represent the complete genetic repertoire of a species [17, 18]. Other genetic variations, such as structural variations (SVs), also play important roles in plant genetics [19, 20], and their potential should be harnessed for crop breeding and improvement. Besides, applying multi-omic (e.g., transcriptomic, proteomic, metabolomic, and epigenetic) bio-data to reveal genetic mechanisms is becoming more practical [21]. It is highly conceivable that systematic integration of multi-omics data could accelerate crop breeding and improvement [22, 23]. Given these considerations, it follows that to aid in increasing the productivity and quality of crops from the perspectives of genomics and genetics, we should (i) construct a genus-level crop pan-genome, or “super-pan-genome” [24], that includes both cultivated and wild accessions within a genus; (ii) include more genetic variations (e.g., SVs) in addition to SNPs/InDels into genomics-assisted crop breeding, and (iii) systematically integrate multi-omics evidence to accelerate crop breeding.

Maize is a staple crop and a model organism for genetic research [25]. Since the first release of the maize B73 reference genome in 2009 [26], more than 40 maize genomes have been released to date. Moreover, multi-omics maize data, including DNA resequencing [27–31], transcriptomic [32, 33], metabolomic [34, 35], proteomic [36, 37], and epigenomic [38] data, have accumulated at the population scale. Recently, pan-maize gene sets have been constructed from the genome assemblies of the 26 founder lines of the Nested Association Mapping (NAM) population [39] and the population-level transcripts of hundreds of diverse lines [40, 41]. The potential effects of SVs on maize phenotypes have also been investigated [17, 20]. However, a pan-genome of the genus *Zea* (pan-*Zea* genome), including maize and wild taxa, and its graphical representation is still lacking. Here, we (i) constructed a pan-*Zea* genome from 11 public genome assemblies and de novo draft assemblies of 721 accessions, including 507 modern maize, 31 landraces, and 183 teosintes; (ii) revealed the patterns of genes and presence/absence variations in the genus *Zea*; and (iii) identified SVs among the maize population and systematically analyzed the potential role of the pan-*Zea* genome and SVs in maize phenotype variations. These resources and analyses will allow us to more comprehensively understand the genetic bases of complex agronomic traits in maize and provide valuable information for future improvements in maize.

## Results

### Pan-Zea genome construction and characterization

**Fig. 1:**
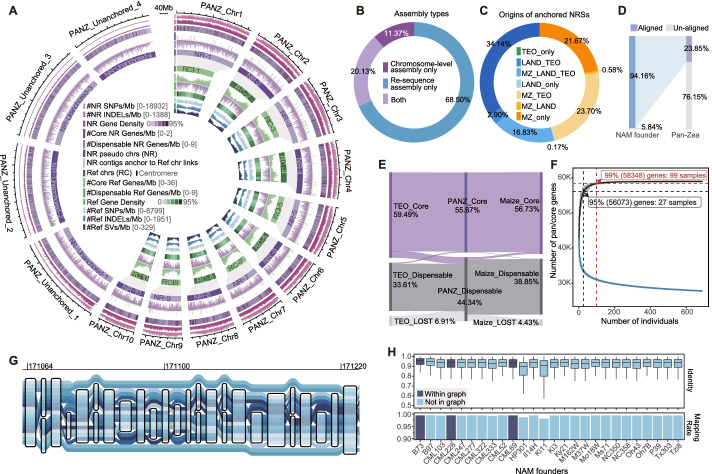
Pan-*Zea* genome, gene, orthologous group PAV, and variant graph genome representation. **A** The genomic landscape of the pan-*Zea* genome. The pan-*Zea* genome (PANZ) comprised the AGPv4 reference genomes (RefChr_1–10) and non-reference sequences (NRINS_1–10 and NRINS_Unanchored_1–4). The reference sequence did not belong to the 10 chromosomes and the related anchored non-reference sequences were not plotted. See the legend in the circle diagram for details. **B** Proportions of different assembly types in the anchored non-reference sequences. “Chromosome-level assembly only”, non-reference sequences that were only found in the 11 chromosome-level genome assemblies; “Re-sequence assembly only”, non-reference sequences that were only found in the 721 *WGS* de novo assemblies. **C** Proportion of anchored non-reference sequences with different sub-group origins. TEO, LAND, and MZ represent teosinte, landrace, and modern maize origins, respectively. **D** Comparison of non-reference sequences from this study to those generated from the founders of maize nested association mapping population (NAM founder). **E** Sankey plot of the proportions of the core and dispensable genes in pan-*Zea* (PANZ), the teosinte sub-group (TEO), and the maize sub-group. **F** Distribution of the number of pan (black) and core (blue) genes along with different numbers of sequenced individuals. See also Additional file 1: Fig. S8D–E. **G** Schematic of the variant graph genome representation for AGPv4 Chr2:171064-171220, with the SNP paths, short InDels, and a large deletion. **H** The identity and mapping rate distribution of the simulated short reads from the genomes of the 26 NAM founders against the variant graph. Dark blue individuals are presented on the variant graph, whereas light blue individuals are not

The pan-*Zea* genome was constructed from the alignments against the maize B73 reference genome V4 (AGPv4) of the de novo draft assemblies from 721 individuals, including 507 diverse maize inbred lines [42], 31 landrace individuals [43], and 183 teosinte individuals [44]. An additional 11 chromosome-level assemblies of the *Zea* genus from previous studies were also included (Additional file 1: Fig. S1–S3, Additional file 2: Table S1, Additional file 3: Table S2, Additional file 11: Supplementary Text S1). The resulting pan-*Zea* genome, with a total length of about 6.71 Gb, comprised ~2.14 Gb of the B73 AGPv4 reference genome (31.83%) and ~4.57 Gb of the non-B73 reference sequences (NRSs, 68.17%). More than half of the NRSs (58.86%) were anchored to the B73 AGPv4 reference genome (Additional file 11: Supplementary Text S1 and Additional file 12: Supplementary Materials and Methods) (Fig. 1A). Of the anchored NRSs, 68.50% were only found in the 721 re-sequence assemblies, and not in the B73 reference genome (Fig. 1B). Interestingly, more than one-third of the anchored NRSs were not present in the modern maize sequence pool, including 34.14% of the teosinte-specific sequences, 0.17% of the landrace-specific sequences, and 2.90% of the sequences shared by teosinte and the landraces (Fig. 1C). Alignments between our pan-*Zea* NRSs and the NRSs generated from the 26 NAM founders (NAM-NRSs, Additional file 11: Supplementary Text S1 and [39]) showed that the pan-*Zea* NRSs included almost all (98.76%) of the NAM-NRSs, as well as many (69.52%) additional NRSs that were not identified in the NAM-NRSs (Fig. 1D). This result indicates that our pan-*Zea* NRSs considerably enlarged our genetic catalog of the maize gene pool.

The gene models and functional annotations for the pan-*Zea* genome were next generated by merging the AGPv4 reference gene annotations with the non-reference genes that were annotated based on a combination of transcript evidence, homologous protein evidence, and ab initio gene predictions (Additional file 1: Fig. S4 and Additional file 12: Supplementary Materials and Methods), resulting in 58,944 genes (39,591 AGPv4 genes and 19,353 non-reference genes, see Additional file 4: Table S3) and 21,167 orthologous groups (Additional file 12: Supplementary Materials and Methods). About 85.82% of the genes were assigned to at least one functional annotation (Additional file 1: Fig. S5).

The gene presence/absence (gPAV) patterns for each maize inbred and teosinte individual (landrace individuals were excluded from the downstream analysis to avoid bias, leaving 691 genotypes for subsequent analysis, see Additional file 12: Supplementary Materials and Methods) were estimated using a read-mapping-based method that maintained robustness among different read depths (Additional file 1: Fig. S6A). The resulting gPAV patterns followed previous reports (Additional file 1: Fig. S6B–D), with an estimated genotyping accuracy of ~99.71% and ~95.84% for true presence and true absence, respectively (Additional file 1: Fig. S6E). Principal component analysis and linkage disequilibrium (LD) rank analyses of the gPAVs revealed that the gPAVs were related to the population structure and were well represented by SNPs with ~97.37% gPAVs displaying high LD with nearby SNPs (Additional file 1: Fig. S7 and Additional file 12: Supplementary Materials and Methods). Next, to investigate the PAVs of genes and the orthologous groups from a population perspective, we identified the “core” (with population-level loss rate not significantly greater than 1%) and “dispensable” (with population-level loss rate significantly greater than 1%) genes and orthologous groups based on the gPAV and the derived orthologous group PAV (oPAV) matrices (Additional file 1: Fig. S8A, Additional file 12: Supplementary Materials and Methods). The results of these analyses revealed that ~44.34% of the pan-*Zea* genes were dispensable, while only ~7.42% of the pan-*Zea* orthologous groups were dispensable (Fig. 1E and Additional file 1: Fig. S8B). An average of 6020 genes displayed PAV patterns between two individuals, with larger differences for two inter-subspecies individuals (~6779 between one teosinte and one maize) than inner-subspecies (~5520 between two maize and ~5635 between two teosintes) (Additional file 1: Fig. S8C). Given our knowledge of the core and dispensable gene/orthologous groups, we estimated the gene/orthologue-group set size for the pan-*Zea* genome and the core genome. The in silico simulation showed that the pan-*Zea* genome (Fig. 1F), as well as the subspecies pan-genomes (pan-maize genome and pan-teosinte genome, Additional file 1: Fig. S8D–E), displayed characteristics of a “closed pan-genome” [45] with plateaus in the size curves, suggesting that we identified almost all of the genes in maize and teosinte. The results show that 27 individuals represented an average of 95% of the pan-*Zea* gene-set (range ~91.9 to ~97.3%), while 99 individuals covered 99% of the pan-*Zea* genes (Fig. 1F).

In addition to the linear representation of the pan-*Zea* genome, we also constructed a graph-based genome (Fig. 1G), including the SNPs, short InDels (<50 bp in length), and SVs (>50 bp in length) (see sections below and Additional file 12: Supplementary Materials and Methods for details of the SVs). Furthermore, we estimated a representation of the variant graph genome using reads simulated from the 26 NAM founder genome assemblies. The results showed that reads simulated from the “not-in-graph” NAM founders (23/26) had compatible mapping rates (99.40% on average) and alignment identities (91.00% on average) with those of the “within-graph” NAM founders (3/26, including B73, with 99.44% and 91.14% for the average mapping rate and alignment identity, respectively) (Fig. 1H), indicating that the variant graph represented the vast majority of the maize genetic repertoire.

### The presence/absence patterns of pan-Zea genes and the orthologous groups

**Fig. 2:**
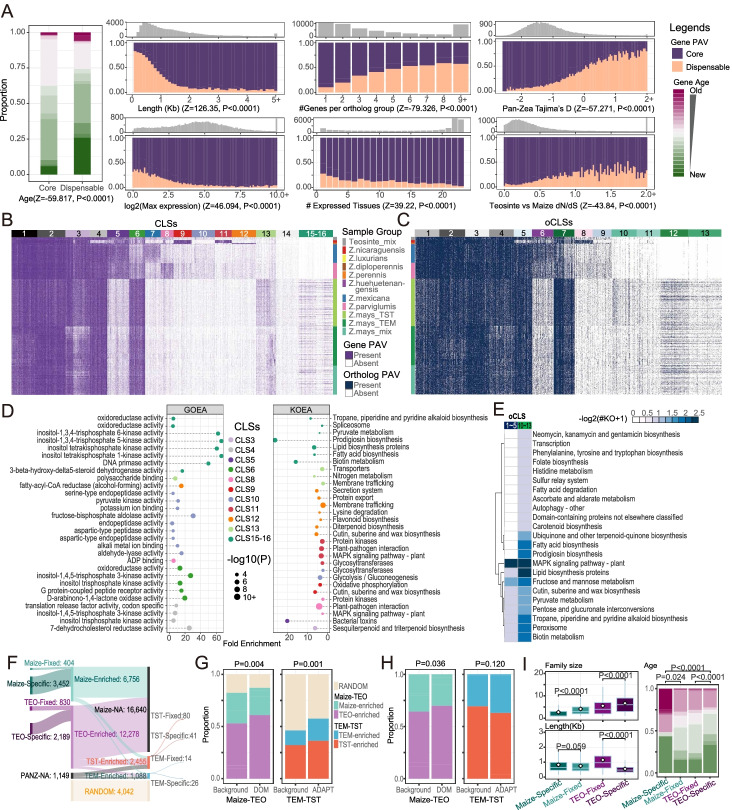
The features of the gene and orthologous group PAVs. **A** Distributions of the core and dispensable genes along with features, including gene age, gene length, number of genes per orthologous group, maximum expression, number of expressed tissues, pan-*Zea* Tajima’s *D* value, and dN/dS between the teosinte and maize populations. The gray histograms in **C**–**H** are the distributions of counts for each feature item. **B**, **C** Distributions of the PAV matrices for the dispensable genes (**B**) and the orthologous groups (**C**) according to the clusters and populations. *X*- and *Y*-axes represent the individuals and genes (or orthologous groups), with the top-most and right-most bars indicating the cluster information and sub-population groups, respectively. **D** The lollipop plots of the GO enrichment analyses (GOEA) and KO enrichment analyses (KOEA) of each gene cluster, with the *X*- and *Y*-axes indicating the fold enrichment and each enriched item, respectively. The point size indicates the *P*-value (only records with corrected *Q* values < 0.05 were plotted). **E** The heatmap plot indicates the KEGG pathways enriched in maize and concentrated in the orthologous group clusters (oCLS10–13) when compared to the pan-*Zea* scatted clusters (oCLS1–5). The color gradient indicates the number of KOs related to the current pathway. **F** Sankey plot of the proportions of unbalanced sub-group genes. **G**, **H** The distributions of random (**G**) and unbalanced (**H**) genes along the domestication (DOM) and adaptation (ADAPT) selective sweep regions. Background, regions that were not in the top 5% of the selective sweep signals. **I** Features of maize-specific and teosinte-specific genes. Shared legends with **A**, **F**, and **G**. The Z-scores and *P*-values were calculated from 10,000 permutations of the Wilcoxon-Mann-Whitney test. TEO, teosinte; TST, tropical/subtropical maize; TEM, temperate maize; RANDOM, balanced across groups; NA, not available due to core or lost in the entire group; the enriched genes were further divided into two categories: “Fixed”, present in all individuals of the current group, and “Specific”, present specifically in the current group (absent in all individuals of the other groups)

An interesting question is which genes are more likely to show gPAVs in the genus *Zea*. To address this question, we investigated the associations between the gPAVs and genic features, including the sub-genome origin, gene age, gene length, orthologue group size, expression levels, and selective constraints (Additional file 12: Supplementary Materials and Methods and Additional file 5: Table S4). The results of these analyses revealed that the gPAV was significantly associated with the genic features under investigation (Fig. 2A). Specifically, they showed that the absence of pan-*Zea* genes was more prevalent among genes that were newly derived, within large orthologous groups, and with genes that were either minimally or tissue specifically expressed. The dispensable genes were more likely to be evolving under relaxed selective constraint than the core genes. These findings are consistent with previous findings that older genes are more essential [46] and associated with higher expression levels and stronger purifying selection [47].

The 691 pan-*Zea* individuals were divided into three maize sub-populations and eight teosinte sub-populations (Fig. 2B and C, [42, 44]). To investigate whether these sub-populations lost genes and orthologous groups evenly, we clustered the dispensable genes into 16 clusters (CLS1–16 in Fig. 2B and Additional file 5: Table S4) and the orthologous groups into 13 clusters (oCLS1–13 in Fig. 2C). The distributions of the gPAVs and oPAVs were associated with the topology of the species tree. The distal-to-maize teosinte subspecies (*Zea nicaraguensis*, *Zea luxurians*, *Zea diploperennis*, *Zea perennis*, and *Zea mays* ssp. *huehuetenangensis*) had more subspecies-enriched genes and orthologous groups than close relatives of maize (*Zea mays* ssp. *mexicana* and *Zea mays* ssp. *parviglumis*).

Enrichment analysis suggested that the gPAV and oPAV clusters may reflect distinct molecular functions among the sub-populations (Fig. 2D–E, Additional file 6: Table S5, Additional file 7: Table S6 and Additional file 1: Fig. S9). Specifically, the teosinte concentrated genes (CLS5, 8, 9, 10, 11, and 12 in Fig. 2A) had enrichment signals, including plant-pathogen interactions, bacterial toxins, biosynthesis of flavonoids, di-/tri- or sesqui-terpenoids, cutin, suberin and wax, and the mitogen-activated protein kinase signaling pathway (Fig. 2D). These enriched pathways are all related to abiotic and biotic stress responses [48]. These findings corroborate the previous finding that reduced genetic diversity during crop domestication leads to the loss of several loci related to the stress response [49], suggesting the potential role of gene loss in the stress-susceptible changes in crops. Comparatively, maize-enriched genes (CLS6, 13, 15, and 16 in Fig. 2A) and orthologous groups (oCLS10-13 in Fig. 2B) were more likely to be related to germination, nutrition, and flavor-related pathways [50, 51]. For example, the maize concentrated orthologous group oCLS7 was enriched in amino acid and protein-related pathways when compared to the teosinte concentrated orthologous group oCLS6 (Additional file 1: Fig. S10), and the orthologous groups that are rarely present in teosintes (oCLS10–13) were enriched in metabolite pathways related to folate, fatty acids, ascorbate, carotenoids, biotin, and various carbohydrates (Fig. 2E).

To further address the contents of teosinte-specific genes and maize-specific genes, we investigated the sub-population gPAV distribution differences in teosinte versus maize (TEO-Maize) and tropical maize versus temperate maize (TST-TEM) in more detail. As results, 51.09% (3452/6756) of the maize-enriched genes were absent in all teosinte individuals (hereafter referred to as maize-specific genes), while 17.83% (2189/12,278) of the teosinte-enriched genes were lost in maize (teosinte-specific genes) (Fig. 2F). We detected 3543 TST-TEM unbalanced (enriched or diminished) genes, with only 1.93% of the sub-group-specific items. A total of 4042 genes were found with no specific distribution preference in any sub-group (random genes, Fig. 2F). Further analysis revealed that the random genes were significantly under-represented in the domestication and adaptation selective sweep regions (Fig. 2G), suggesting that sub-group unbalanced genes were selected during maize domestication and adaptation. Thus, we compared the proportion of different sub-group-enriched genes between selected and background regions (Fig. 2H). This analysis revealed that although the distribution of sub-group-enriched genes was not significantly different between TEM and TST maize, the teosinte-enriched genes were more likely to appear in domestication regions (Fig. 2H), suggesting that some teosinte genes were selected to be lost during domestication.

Another notable question is the pattern of gain-or-loss of teosinte/maize-specific genes, considering that a gene can become group-specific either through (i) loss of all members of the other group or (ii) gained from exogenous sources that were not available to the other group. While the gPAVs showed a predominance of teosinte-enriched genes, the oPAV clusters showed the opposite trend in that more orthologous groups were enriched in maize than in teosinte (Additional file 1: Fig. S11A and B), and the maize-enriched orthologous groups, particularly the maize-specific groups, tended to be smaller (Fig. 2I and Additional file 1: Fig. S11C). The comparisons of group-specific gene features indicated that the teosinte-specific genes showed a typical “easy-to-lose” pattern within larger families of shorter and newer. However, the maize-specific genes showed an opposite pattern of smaller family size and longer and considerably older genes (Fig. 2I). These analyses suggest that the teosinte-specific genes most likely resulted from gene loss, while at least a subset of the maize-specific genes were derived from resources outside of the *Zea* genus, perhaps through horizontal gene transfer from bacteria [52], fungi [53], or pests [54] or from lateral gene transfer with other grasses [55].

### Complementing the maize genetic variation map with structural variations

**Fig. 3:**
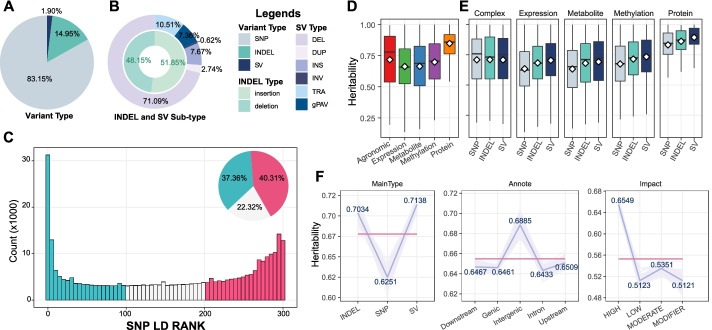
Features of the maize genetic variation map. **A** Pie plots of the proportions of SNP, InDel, and SV. **B** Pie plots of the proportions of InDel and SV sub-types. **C** Distribution of the number of SV *r*^2^ ranks (0–300) that are above the SNP-based median *r*^2^ value (referred to as SNP LD rank value) for common SVs. **D** Distribution of heritability within different omics trait classes. The white diamond within each box indicates the mean value, and hereinafter. **E** Distribution of heritability of the different variant types among the different omics trait classes. **F** Trend lines of heritability among the different genetic variant features. Light purple lines indicate the feature item’s mean heritability of all traits for each randomization. Blue lines indicate each feature item’s mean heritability for all 100 randomizations. Red lines indicate the mean heritability of all of the feature items. See also Additional file 1: Fig. S13

SVs have received significant attention and are responsible for various complex traits in many species [13, 18, 56]. Previous research has constructed a high-density haplotype map of the genus *Zea* using the same populations by mapping with the B73 reference genome [44]. To complement the genetic variation map and estimate the impact of SVs on the maize phenotypic variations, we constructed a comprehensive SV map by integrating evidences from variant graphs, comparative genomics, and short-read alignments. We filtered the SVs with a set of strict conditions, and only the common (MAF > 0.05) SVs within the maize population were retained (Additional file 1: Fig. S12 and Additional file 12: Supplementary Materials and Methods). We detected 274,649 common SVs, including 181,874 deletions (DELs), 19,628 insertions (INSs), 26,894 translocations (TRAs), 7020 duplications (DUPs), and 1577 inversions (INVs), as well as 18,828 common gPAVs from the aforementioned gPAV matrix. A total of 11,208,912 SNPs and 2,015,663 InDels (1,045,218 short-insertions and 970,445 short-deletions) that were common in the maize population were extracted from the *Zea* haplotype map and were combined with the common SVs identified in the current study to form a maize common genetic variation map for downstream analyses (Fig. 3A and B and Additional file 1: Fig. S13A-B). Considering the repeat-rich nature of maize, we also assigned the SVs to their closest transposable elements (TEs) according to physical overlap and sequence similarity (Additional file 12: Supplementary Materials and Methods) and found that ~60.03% of the SVs were TE-related (Additional file 1: Fig. S13B), indicating the TE origin of a sizeable proportion of SVs in maize. The size of most of the genotyped common SVs was smaller than 5 Kb (Additional file 1: Fig. S13C), which may have been caused by the limitation in WGS short reads [56]. The SNPs, InDels, and SVs showed similar MAF distribution patterns that skewed toward rare variants (Additional file 1: Fig. S13D). The estimate of the representativeness of each SV by nearby SNPs revealed that 37.36% of the SVs showed low LD levels with nearby SNPs (Fig. 3C and Additional file 12: Supplementary Materials and Methods), suggesting that these SVs could harbor information that cannot be represented by nearby SNPs.

With the representative genetic variation map that covered the typical genetic variation types, we conducted comprehensive investigations on the differences in the partitioning of narrow-sense heritability (*h*^*2*^) among the different genetic variation types. To address this, we estimated the phenotypic variance explained by the genetic variations from a collection of complex agronomic phenotypes and multi-omics molecular trait data, including gene expression, metabolites, protein contents, and DNA methylation (Additional file 8: Table S7 and Additional file 12: Supplementary Materials and Methods). This analysis revealed that each class of the multi-omics traits displayed high heritability (average values of 0.72, 0.66, 0.67, 0.70, and 0.85 for agronomic traits, expression, metabolites, DNA methylation, and proteins respectively; see also Fig. 3D), indicating their capability to characterize the heritability patterns among the genetic features. The *h*^*2*^ values of the SVs were higher than those of the SNPs and InDels in the multi-omics molecular traits (Fig. 3E) even though there were significantly more SNPs (43.8 times) and InDels (7.9 times) than SVs. To estimate the *h*^*2*^ differences in an unbiased manner, we binned, or partitioned and randomized, the genetic variations to keep each of the compared features, including MAF, the SNP LD rank, variant types/sub-types, genomic locations, and impacts on the genes, in the same volume (see Additional file 12: Supplementary Materials and Methods). As a result, the SVs showed a more clearly decreasing *h*^*2*^ pattern with the increase in the MAF than SNPs/InDels. The *h*^*2*^ values of the SVs were negatively correlated with the SNP LD rank values, in contrast to those of InDels (Additional file 1: Fig. S13E). When came into the same volume, SVs (with an average *h*^*2*^ of 0.71, range 0.70–0.72 for each randomization) explained an average of 14.19% and 1.48% more phenotypic variance than the SNPs (with average *h*^*2*^ of 0.63, range 0.60–0.65 for each randomization) and InDels (with an average *h*^*2*^ of 0.70, range 0.68–0.72 for each randomization), respectively (Fig. 3F). Higher *h*^*2*^ values were found in the intergenic variants, genic variants with a high impact on genes, gPAV, and TRA type of SVs, as well as the LTR and helitron-related SVs (Fig. 3F and Additional file 1: Fig. S13F). These findings suggest that SVs are more likely to lead to functional changes than other variants.

### The impact of the pan-Zea genome and structural variations on maize phenotypic variations

**Fig. 4:**
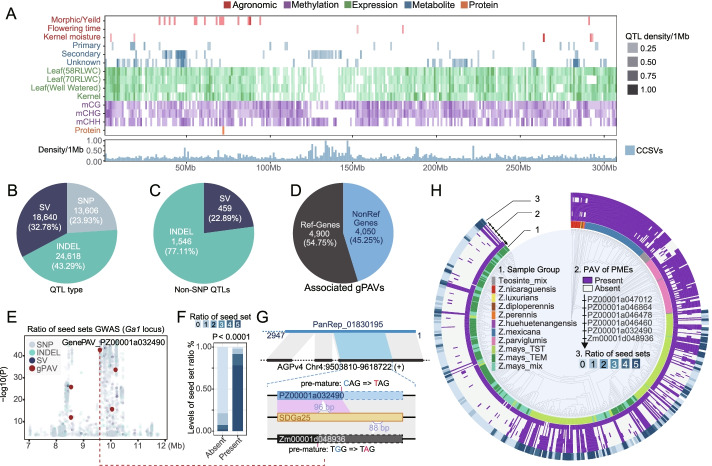
Characteristics of the phenotype associated QTLs, genes, and causal variants. **A** Distribution of the associated QTLs, genes, and causal variations (example with chromosome 1, see Additional file 1: Fig. S14 for the whole genome). The heatmap represents the QTL density within each 1-Mb window, while the histograms indicate the density of the candidate causal variants that were normalized with the number of all variants within the 1-Mb window. **B** Proportions of QTL types (QTLs lead with different variant types). **C** Proportions of INDEL/SV-specific QTLs (QTLs that cannot be detected by SNPs). **D** Proportions of associated gPAVs for the reference genome genes (Ref-Genes) and non-reference genome genes (NonRef Genes). **E** Manhattan plot of the association result of the *Ga1* locus related to the ratio of seed sets, with gPAVs highlighted in red. **F** The proportion of different levels of seed set ratio related to the absence/presence of PZ00001a032490; the larger the number, the higher the seed set ratio. **G** The genome alignment indicates the anchoring of the NRS (PanRep_01830195) on the AGPv4 genome, and the schematic plot illustrates the differences between the three *PME* genes (PZ00001a032490, SDGa25, and Zm00001d048936). Solid rectangles indicate the gene coding sequence, while the dashed rectangles indicate the missing coding part related to SDGa25. Gray ribbons indicate the matched blocks. Pink ribbons indicate the matched CDS blocks. **H** Distribution of the PAV patterns (track 2) of the six *PME* genes and the ratio of seed sets (track 3) according to the structure tree of pan-*Zea* individuals (track 1). **I** Distribution of the number of presented *PME* genes (# PMEs) related to the levels of the seed set ratio. The gray histogram is the distribution of total sample numbers (count) according to the *X*-axis, while the colored histogram indicates the proportions. The *P*-value was calculated from 10,000 permutations of the Wilcoxon-Mann-Whitney test

The comprehensive genetic variation map and the multi-level phenotypes provided opportunities to further investigate the potential effects of different genetic and genomic features on the phenotypes. To this end, we performed genome-wide association analyses for the complex traits (agronomic traits, metabolites, and protein contents) and local association analyses for the molecular traits (gene expression and DNA methylation) (see Additional file 12: Supplementary Materials and Methods for details). A total of 21,255 non-redundant QTLs with a median QTL interval of ~152.77 Kb were identified for 21,206 different traits (Fig. 4A, Additional file 1: Fig. S14 and Fig. S15). About 32.78% of the identified QTLs were SV-QTL (Fig. 4B). Among them, 459 QTLs were SV-specific (could only be identified by SVs, Fig. 4C and Additional file 9: Table S8). The proportion of SV-QTLs was much higher than the proportion of SVs in all of the variants, indicating that SVs are more likely to lead to functional changes.

About 47.54% (8950) of the tested gPAVs had associated signals (Fig. 4D and Additional file 12: Supplementary Materials and Methods), suggesting the functional importance of these gPAVs. An excellent example is shown in Fig. 4E–H in which a premature pectin methylesterase (*PME*) gene (Zm00001d048936) at the maize *gametophyte factor1* (*Ga1*) locus was reported to be causative of unilateral cross-incompatibility [163]. In our results, the major QTL contained at the *Ga1* locus was also detected to underlay the unilateral-crossing seed set ratio (a representation of the unilateral cross-incompatibility-related trait, see also Additional file 12: Supplementary Materials and Methods). Additionally, the presence of a non-reference gene (PZ00001a032490) was significantly related to a high ratio of seed setting (*P* = 1.91E−43, *R*^2^ = 0.51, Fig. 4E and F). The non-reference gene PZ00001a032490 was anchored to the position where the premature *PME* gene Zm00001d048936 was located in AGPv4. Further alignment showed that PZ00001a032490 was highly similar to the intact *PME* gene in SDGa25 reported by Zhang et al. [163] and the Zm00001d048936 flanking sequence, with a 96-bp DEL and a premature nonsense point mutation (Fig. 4G). Although both were premature, the gene body of PZ00001a032490 had an additional 126 bp coding sequence that was absent in Zm00001d048936 (Additional file 1: Fig. S16), which contributed to the PAV polymorphism that failed to be detected in Zm00001d048936 (Fig. 4H). These results indicate that although most of the gPAVs were represented by nearby SNPs, leveraging the pan-*Zea* genome and gPAVs was highly useful in identifying candidate genes, which could not be directly detected using a single reference genome. Notably, in addition to the two PME genes (Zm00001d048936 and PZ00001a032490) in the *Ga1* locus, we also found four more non-reference *PME* genes (PZ00001a047012, PZ00001a046864, PZ00001a046478, and PZ00001a046460) (Additional file 1: Fig. S17). These *PME* genes all showed similar presence/absence patterns with PZ00001a032490, and the presence of these *PME* genes was enriched in teosintes (with presence ratio of ~85.97% in teosintes and ~40.35% in maize, Fisher’s exact test *P*-value < 2.2e–16, Fig. 4H). The function of these newly identified *PME*-like genes merits further study.

Another question of concern is to estimate the feature priorities to identify the causative genetic variations underlying the phenotypic variations. Thus, we identified the candidate causal variant (CCV) set using a Bayesian-based statistical fine-mapping algorithm [146]. A total of 807,787 genetic variations (3.25% SVs, 15.73% InDels, and 81.02% SNPs) were kept as CCVs, as they were within the 95% confidence interval of the causal variant set for at least one trait. On average, the statistical fine-mapping kept ~18 variants as CCVs from ~229 nominally associated variants (with *P* < 0.001, see Additional file 12: Supplementary Materials and Methods) for each QTL. The number of CCVs was poorly correlated with the QTL quality score, the significance of the leading variant, and the number of genetic variations within the QTL (Additional file 1: Fig. S18A), indicating that CCVs could reflect additional information that cannot be represented by using the leading variants alone. The estimate of the effect sizes of the CCVs showed that SVs and gPAVs had a larger effect size than that of the SNPs/InDels (Additional file 1: Fig. S18B). The general feature enrichment analyses between the CCVs and the nominally associated variations (see Additional file 12: Supplementary Materials and Methods) showed that the SVs, particularly INSs and gPAVs, were more likely to be enriched in the causal variant sets than SNPs or InDels (Additional file 1: Fig. S18C). Specifically, INSs were enriched in expression, metabolites, and methylation, while gPAVs were only found enriched in expression (Additional file 1: Fig. S19). For SVs related to different TE classes, the helitron and TIR-related SVs were more likely to be causal than the LTR-related SVs. Genic variants, particularly those with a high impact on genes, were more likely to be causal (Additional file 1: Fig. S18C).

To further investigate the effect of different genetic variations on gene expression, we estimated the enrichment of CCVs in the *cis*-eQTLs along their distance to the transcription start site (TSS). The results showed that the CCVs were enriched in TSS-nearby regions (“Causal variants” track in Additional file 1: Fig. S20), following previous results [164]. Further investigation revealed that the SVs displayed the waviest trend for the fold enrichment changes along distances to the TSS than INDELs and SNPs (“SNP,” “INDEL,” and “SV” tracks in Additional file 1: Fig. S20). This pattern indicated that the TE-related SVs were more likely to be enriched in the upstream regions of TSSs (“TE-related SVs” track in Additional file 1: Fig. S20). These findings suggest that rather than directly affect TSSs or gene body regions, SVs (particularly TE-related SVs) would be more likely to affect gene expression by affecting nearby upstream regions of the gene, where most *cis*-regulatory elements (CREs) are located [165].

**Fig. 5:**
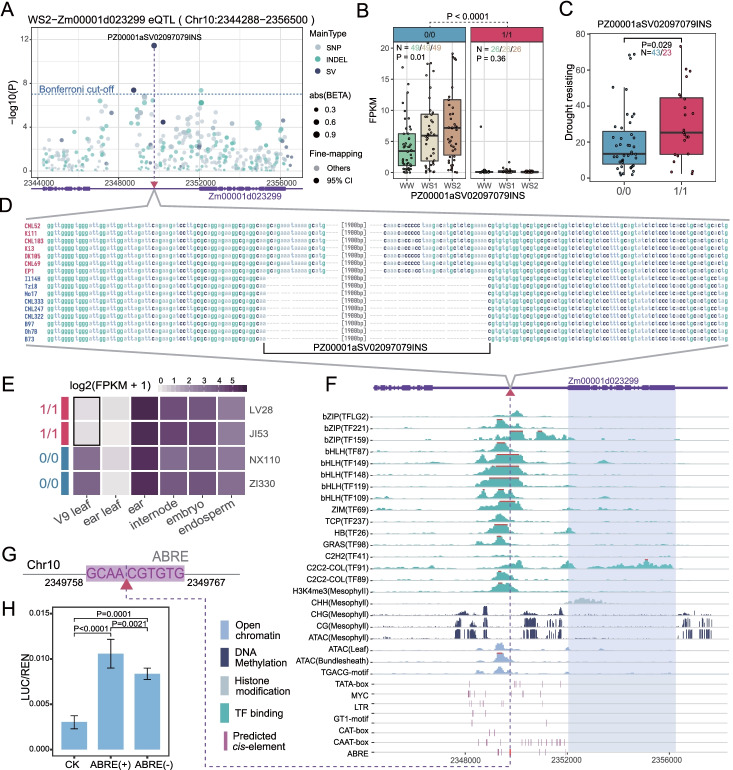
An example showing that inserting a transposon suppressed gene expression by destroying the tissue-specific TF binding element. **A** Manhattan plot of an eQTL (Chr10:2344288-2356500) of Zm00001d023299 expression in drought-stressed leaves. Note that the eQTL has no associated SNPs that passed the Bonferroni-corrected *P*-value cut-off, the leading variant had a large effect size (BETA), and the highest probability for the cause (fine-mapping) was an insertion (PZ00001aSV02097079INS) located 2269 bp upstream the target gene. **B** The influence of PZ00001aSV02097079INS on the Zm00001d023299 expression related to different levels of drought stress. WW, well-watered; WS1, 70% relative leaf water content (RLWC); WS2, 50% RLWC; 0/0, the reference allele; 1/1, the alternative allele. The significant differences were evaluated using the Kruskal-Wallis test. **C** The influence of PZ00001aSV02097079INS on the drought resistance-related trait (survival rate under drought stress). The significant differences were evaluated using the Wilcoxon-Mann-Whitney test. **D** The validation of PZ00001aSV02097079INS alleles by multiple genome alignment of the maize reference-level genomes. The genomes with reference and alternative alleles are blue and red, respectively. **E** Comparison of the Zm00001d023299 expression patterns in different tissues of four CUBIC parents with (red) or without (blue) the PZ00001aSV02097079INS insertion. The expression difference in the tenth leaf at the V9 stage is highlighted by the black rectangle. **F** The epigenetic patterns and TF binding sites of maize leaves, and the predicted *cis-*regulatory elements in the targeted eQTL region. Only items showing distinct peaks near the PZ00001aSV02097079INS were plotted for the epigenetic patterns and TF binding sites. Only the elements located in the positive strand and 4 Kb upstream of the target gene were plotted for the predicted CREs. **G** An illustration showing that the PZ00001aSV02097079INS inserted in a predicted *cis*-acting element involved in the abscisic acid responsive element (ABRE). **H** The relative promoter activities represented by the ratio of luciferase (LUC) to *Renilla* luciferase (REN) activity (LUC/REN in *Y*-axis) for the treatments (*X*-axis), including pGreenII 0800-LUC without a minimal promoter (CK), with ABRE (ABRE+) and without ABRE (ABRE−), see also Additional file 1: Fig. S23. The *P*-values were calculated from 10,000 permutations of the *t*-test

A detailed example is illustrated in Fig. 5. The impact of a SV on the expression of Zm00001d023299, a zinc finger CCCH domain-containing protein (ZEAMAP [57]) that has been previously proposed to be a candidate QTL (marked as IDP103 in MaizeGDB), related to drought and ultraviolet stressors [58] and was highly expressed in response to various stressors in maize (Additional file 1: Fig. S21). In the current study, an SV-specific eQTL related to the expression of Zm00001d023299 in drought-stressed leaves harbored a CCV named PZ00001aSV02097079INS (Fig. 5A). The expression of Zm00001d023299 in leaves [33] responded to different drought treatment levels, and the presence of PZ00001aSV02097079INS suppressed gene expression in leaves (Fig. 5B). The presence of PZ00001aSV02097079INS could also increase the survival rate of maize under drought stress [59] (Fig. 5C). Comparisons among maize genomes indicated that PZ00001aSV02097079INS is a 1947-bp Harbinger-transposon-like sequence (Additional file 1: Fig. S22) inserted 2269 bp upstream of Zm00001d023299 (Fig. 5D). This evidence suggests that PZ00001aSV02097079INS could be one of the causes of maize drought resistance by suppressing the expression of Zm00001d023299 in leaves. An investigation into the expression patterns in different tissues of four maize founder individuals from the Complete-diallele design plus Unbalanced Breeding-like Inter-Cross (CUBIC) population [60] with/without PZ00001aSV02097079INS showed that the impact of the PZ00001aSV02097079INS was restricted to the elongation stage (V9) of leaves (Fig. 5E). These associations were also validated in CUBIC offspring (Additional file 1: Fig. S23). These findings suggest that the suppressed expression was most likely caused by affecting tissue-specific CREs. Further epigenetic evidence and TF binding sites in maize leaves [61] revealed typical patterns of active regulatory elements near the inserted region of PZ00001aSV02097079INS, which lacked DNA methylation, and signals of several TFs, particularly the basic leucine zipper (bZIP) and basic/helix-loop-helix (bHLH) TF families (Fig. 5F). There were numerous predicted CREs within the upstream region of the Zm00001d023299 target gene (Fig. 5F), and remarkably the insertion of PZ00001aSV02097079INS was located exactly within a predicted abscisic acid responsive element (ABRE) motif (Fig. 5G). The activity of the predicted ABRE was validated by a luciferase experiment demonstrating that obliterating the function of the ABRE significantly reduced the expression of its downstream target gene (Fig. 5H, Additional file 1: Fig. S24, and Additional file 10: Table S9). This finding suggests that PZ00001aSV02097079INS may have affected the expression of Zm00001d023299 in maize leaves by transposing into an ABRE motif region and blocking the binding of some tissue-specific TFs (bZIPs and/or bHLHs), which suppressed the tissue-specific expression of Zm00001d023299 in leaves, and contributed to drought tolerance in maize.

In conclusion, we revealed the genomic feature priorities that were more causatively associated with multi-omics-level phenotype variations. We showed that leveraging SVs and pan-*Zea* genome-based gPAVs can be used to detect causative associations related to important agronomic phenotypic variations that could not be directly identified using single genome-based SNPs.

## Discussion

### Community resources to accelerate maize molecular breeding

To meet the upcoming food production challenge, considerable efforts are required in maize genomics and genetics to further understand the genetics of agronomic traits and increase the efficiency of molecular breeding. In this study, we constructed a genus-level pan-genome for *Zea*, identified the pan*-Zea* gene and orthologous group presence/absence patterns, complemented the maize genetic variation map by including common SV types, and systematically investigated the potential genetic and genomic feature priorities for multi-omics traits by identifying the trait-associated QTLs and candidate causal variants from GWAS summary statistics data. These resources will provide useful information for maize breeding in the following three ways.

First, the wild relatives of maize have shown their potential for increasing stress tolerance and overall yield [62–64]. The de novo fragmented assemblies of the 183 teosinte individuals that covered all seven teosinte subspecies, the pan*-Zea* genome, and the functional annotation of the pan*-Zea* genes and orthologous groups could all be useful alternatives. The pan*-Zea* gene-set size analyses indicated the ability to cover ~95% of the pan*-Zea* genes with a random sampling of 27 individuals, which provides general guidance for individual selection. Furthermore, the gene and orthologous group PAVs were categorized into sub-population-enriched clusters, and these clusters were often enriched with different biological functions. These results will enable breeders to select specific individuals for target traits based on genomic evidence.

Next, we assessed the potentiality of using SVs and the high-density genetic variation map for maize genome-assisted breeding. We constructed a comprehensive SV genotype matrix including all common SV types by combining the evidence from whole-genome comparisons, NGS mapping, and graph-based genotyping. The SV genotypes, along with the gPAV matrix and the previously reported SNPs and InDels from the same association mapping panel were composed into a comprehensive genetic variation map for maize genome-assisted breeding. Based on this genetic variation map and the multi-omics trait variation data of agronomic phenotypes, metabolites, expression, proteins, and methylations, (i) many of the SVs (~37.36%) were not well represented by nearby SNPs; (ii) SVs explained more heritability than SNPs and InDels in the same volume; (iii) SVs were more likely to be the cause of phenotype variation than SNPs and InDels; and (iv) SVs can represent QTLs that cannot be detected by SNPs only. These findings will enhance maize genome-based breeding in the future.

We also analyzed the practical value of the summary association statistics, QTL, associated genes, and causal variants for the multi-omics traits. The summary association statistics have shown their forces by being broadly used for humans in analyses involving gene-based association tests, fine-mapping, polygenic prediction, and cross-trait analyses [65]. While GWAS have been successful in decoding genotype-phenotype associations in maize [66], there is still a lack of comprehensive public release of summary association statistics for this crop. Here, a comprehensive genetic variation map was developed based on the summary association statistics for the multi-omics traits from a widely used maize association mapping panel [42, 66]. This map could be a resource for more specific analyses, such as imputing genotypes or discovering variant associations with small effects using meta-analyses. A challenge for genome-assisted breeding in the big data era is to explain or predict the biochemical and macroscopic level phenotypes from the underlying genomic and genetic information under different environmental conditions, which have spawned various machine learning applications to improve crops [67]. Thus, the associated QTL and candidate causal variants could be useful resources for optimal weighting of the marker information into genomic selection models [68].

### Untangling the genotype-phenotype relationship by leveraging the pan-Zea genome and structural variation map

A substantial proportion of the challenges and rewards in crop genetics are dependent upon understanding the genetic architecture of complex agronomic traits. Considerable progress has been made to untangle the puzzle of maize phenotypic variations [34, 38, 69–71], yet it still lacks global insight from the perspective of multi-omics integration. Our surveys on heritability of genetic variant feature partitions and the genetic feature priorities for the causes of the phenotypic variations provide an overview of the genetic architecture of agronomic traits in maize.

Estimates and randomization of narrow-sense heritability have shown high levels of maize omics phenotypic variance explained by genome-wide additive genetic factors. Intergenic variations, which were the majority of GWAS association hits, explained most of the phenotypic variations. However, enrichment of candidate causative variations showed that intergenic variants were less likely to be the cause of the GWAS QTL (which need to have large enough effects to be detected) than genic variants. These findings suggest the ubiquitous polygenic nature of maize agronomic traits, a largely additive genotype-to-phenotype relationship in maize, and that non-coding sequences may more likely contribute by adding multiple variants with small effects.

In addition to the general genotype-phenotype association patterns, we have also shown the potential of leveraging the pan-*Zea* genome, the comprehensive genetic variation map, and population-level multi-omics data to reveal genotype-phenotype relationships. Extra efforts would be needed to determine the underlying cause within the QTL when using a single reference genome, even if the association QTL could be detected through SNP-based analyses, particularly when the causative genes are absent in the reference genome. In our results, at least 32.83% of the pan-*Zea* genes were absent in the commonly used maize reference genome. We detected the associations between the causative gPAV and the unilateral cross-incompatibility in maize by harnessing the pan-*Zea* genome. In addition, by leveraging multi-omics data and statistical fine-mapping, we propose the potential mechanism of how a TE-derived SV affected drought resistance in maize by tissue-specific changes in gene expression. These cases have not only shown the practicability of harnessing the pan-*Zea* genome and SVs to substantially reduce the workload of genome-assistant breeding but have highlighted the potential of the pan-*Zea* genome and the SV map to better understand the internal mechanism of the associations between genetic and phenotypic variations, thus facilitating maize breeding and improvement.

## Conclusions

In summary, we constructed a pan*-Zea* genome, analyzed the gene presence/absence patterns, and investigated the impact of the pan*-Zea* genome and different genetic variant features on maize biochemical and phenotypic variations. These findings will provide useful information for unraveling the genetic architecture of maize complex agronomic traits, accelerate maize molecular breeding, and improve our understanding of maize domestication and adaptation procedure. Still, some limitations have hampered more informative pan*-Zea* genomic and genetic results in the current study.

Perhaps the most obvious holding-back of the pan*-Zea* genome in the current study is the lack of teosinte reference genomes and population-level deep long-read sequencing data for teosintes, landraces, and elite maize. With the fragmental assemblies generated from deep-WGS reads, we had to perform additional data polishing steps to ensure credibility, which inevitably affected the volume of the results, and led to an underestimate of the teosinte sequences and genes within the pan-*Zea* genome, the large and rare SVs, and the untangled variations in the highly repetitive regions.

Foreseeable actions that could address these limitations and boost our understanding of maize breeding and improvement would be to (i) enrich the pan*-Zea* genomic information pool with reference genomes and population-level long-read sequences of teosintes and maize landraces, (ii) enlarge the pan*-Zea* genetic variation matrices and their associations with more biochemicals, (iii) refine the genetic interactions by investigating causation and pleiotropy, and (iv) decrypt the regulatory network of maize phenotypic variation by combining the genetic interactions with other bio-networks, such as interactomes [72] and cell-cell communications [73]. The concept of the pan-genome has expanded from the whole gene and sequence set to the whole set of genomic and genetic variations within a genus. Moreover, with the development of reference-free whole-genome alignments [74] and genome graphic representations [8], it may be time to change the maize reference genomes [7]. We have many reasons to be optimistic when facing the imminent challenges of increasing the productivity and quality of crops with our current resources and the anticipated upcoming progress in crop genomics and genetics.

## Methods

The detailed materials and methods, including (i) collection of genomic and transcriptomic data, (ii) de novo assembly, whole-genome comparison, and pan-genome construction, (iii) pan-genome gene annotation, (iv) pan-*Zea* gene and ortholog group analyses, (v) characterizing gene features, (vi) genotyping and characterizing the maize genetic variation map, (vii) variant graph constructing, reads simulation, and mapping, (viii) phenotype data collection and normalization, (ix) estimating of narrow-sense heritability, (x) identifying trait-associated QTLs, genes, and variants, (xi) analyses of gPAV and SV cases, and (xii) miscellaneous statistical analyses and visualizations, are available in Additional file 12: Supplementary Materials and Methods.

## Supplementary Information

Additional file 1: Figure S1. Statistics of pan-*Zea* NGS *de novo* assemblies. Figure S2. Schematic diagram illustrated the filtering of assembly-to-assembly alignment. Figure S3. Sketch of the non-reference sequence anchoring and clustering pipeline. Figure S4. Pan-*Zea* gene annotation and functional annotation pipeline. Figure S5. Statistics of the proportions of functionally annotated genes. Figure S6. Validation of the gene present and absent variations (gPAVs). Figure S7. PCA and SNP LD rank analysis of gPAV. Figure S8. Core and dispensable genes and ortholog groups. Figure S9. KEGG pathways of each dispensable ortholog group cluster. Figure S10. The KEGG pathways enriched in maize concentrated ortholog groups when compared with teosinte concentrated ortholog groups. Figure S11. Distribution of sub-population enriched genes and ortholog groups. Figure S12. The sketch of SV calling and genotyping pipeline. Figure S13. Additional features of the maize genetic variation map. Figure S14. Distribution of the associated QTLs and causal variations along the chromosome. Figure S15. Distribution of the features of associated QTLs, genes and causal variations. Figure S16. Multiple sequence alignment of the PME genes in the *Ga1* locus. Figure S17. Multiple sequence alignments of the protein sequences of pan-*Zea* PME genes. Figure S18. Statistics of candidate causal variants. Figure S19. Enrichment of causal variants features within different omics trait classes. Figure S20. Enrichment of cis-eQTL causal variants along different distance to TSS. Figure S21. Expression patterns of the candidate genes of the example SV-QTLs. Figure S22. Blast graphic summary of PZ00001aSV02097079INS. Figure S23. Distribution of the Zm00001d023299 expression patterns in the tenth leaf of V9 stage in the CUBIC offspring population related to the haplotypes flanking 500Kb of Zm00001d023299. Figure S24. The diagram of vectors used in the luciferase reporter assays of Zm00001d023299 promoter with (A) or without (B) the predicted ABRE.

Additional file 2: Table S1. Estimating WGS de novo assemblies by whole genome comparison between WGS de novo assemblies and reference genome sequences of B73, SK, Mo17 and HZS.

Additional file 3: Table S2. Public genome assemblies used in the pan-*Zea* genome construction.

Additional file 4: Table S3. Statistics of pan-*Zea* gene annotations

Additional file 5: Tables S4. Pan *Zea* gene features.

Additional file 6: Table S5. Enrichment of molecular function GO items for each gene PAV cluster.

Additional file 7: Table S6. Enrichment of KEGG ontology for each gene PAV cluster.

Additional file 8: Table S7. Statistics of the complex and molecular trait variation data in this study.

Additional file 9: Table S8. Information of QTLs that cannot be detected by SNPs.

Additional file 10: Table S9. Primers used in the luciferase reporter assay of the activity of the predicted ABRE positioned upstream of Zm00001d023299.

Additional file 11: Supplementary Text S1.

Additional file 12: Supplementary Materials and Methods [88–159].

Additional file 13. Review history.

## Data Availability

The accessions of the whole-genome sequencing reads of the pan-*Zea* individuals could be retrieved from GenBank: the maize association mapping panel lines [75], the teosinte lines [76], and the maize landrace lines [77]. The accessions of the 11 publicly available genome assemblies are listed in Additional file 2: Table S1. The pan-*Zea* RNA sequencing data (RNA-Seq) were retrieved from GenBank: the RNA-Seq of the kernels of the AMP lines [78] and the RNA-Seq of the leaves of teosinte lines [79]. The genome assemblies of the maize NAM founders were downloaded from MaizeGDB [80]. The datasets generated in this paper, including the fragmental de novo assemblies of the 701 pan-*Zea* individuals, the pan*-Zea* genome and annotations, the maize structural variation map, and the gene PAV matrices, have been deposited in the China National GeneBank Sequence Archive (CNSA) [81] and figshare [82]. The above data, along with the graphic representation of pan-*Zea* genome and the GWAS summary data for QTL regions, could also be accessed in the download page of the ZEAMAP database [83] and the China National Center for Bioinformation (CNCB) [84]. The related analysis scripts, pipelines, and source codes are available at Github under MIT license and Zenodo: pan-*Zea* genome construction pipeline [85, 160], SV calling and genotyping scripts [86, 161], and miscellaneous analysis scripts [87, 162].
