# Supplementary material for: A pan-Zea genome map for enhancing maize improvement
Source: Genome Biol. 2022 Aug 23;23:178. doi: 10.1186/s13059-022-02742-7 (PMC9396798; doi:10.1186/s13059-022-02742-7)
Supplement: Supplementary file 1 — Additional file 1: Figure S1. Statistics of pan-Zea NGS de novo assemblies. Figure S2. Schematic diagram illustrated the filtering of assembly-to-assembly alignment. Figure S3. Sketch of the non-reference sequence anchoring and clustering pipeline. Figure S4. Pan-Zea gene annotation and functional annotation pipeline. Figure S5. Statistics of the proportions of functionally annotated genes. Figure S6. Validation of the gene present and absent variations (gPAVs). Figure S7. PCA and SNP LD rank analysis of gPAV. Figure S8. Core and dispensable genes and ortholog groups. Figure S9. KEGG pathways of each dispensable ortholog group cluster. Figure S10. The KEGG pathways enriched in maize concentrated ortholog groups when compared with teosinte concentrated ortholog groups. Figure S11. Distribution of sub-population enriched genes and ortholog groups. Figure S12. The sketch of SV calling and genotyping pipeline. Figure S13. Additional features of the maize genetic variation map. Figure S14. Distribution of the associated QTLs and causal variations along the chromosome. Figure S15. Distribution of the features of associated QTLs, genes and causal variations. Figure S16. Multiple sequence alignment of the PME genes in the Ga1 locus. Figure S17. Multiple sequence alignments of the protein sequences of pan-Zea PME genes. Figure S18. Statistics of candidate causal variants. Figure S19. Enrichment of causal variants features within different omics trait classes. Figure S20. Enrichment of cis-eQTL causal variants along different distance to TSS. Figure S21. Expression patterns of the candidate genes of the example SV-QTLs. Figure S22. Blast graphic summary of PZ00001aSV02097079INS. Figure S23. Distribution of the Zm00001d023299 expression patterns in the tenth leaf of V9 stage in the CUBIC offspring population related to the haplotypes flanking 500Kb of Zm00001d023299. Figure S24. The diagram of vectors used in the luciferase reporter assays of Zm00001d023299 promoter with (A) o [file 13059_2022_2742_MOESM1_ESM.pdf]

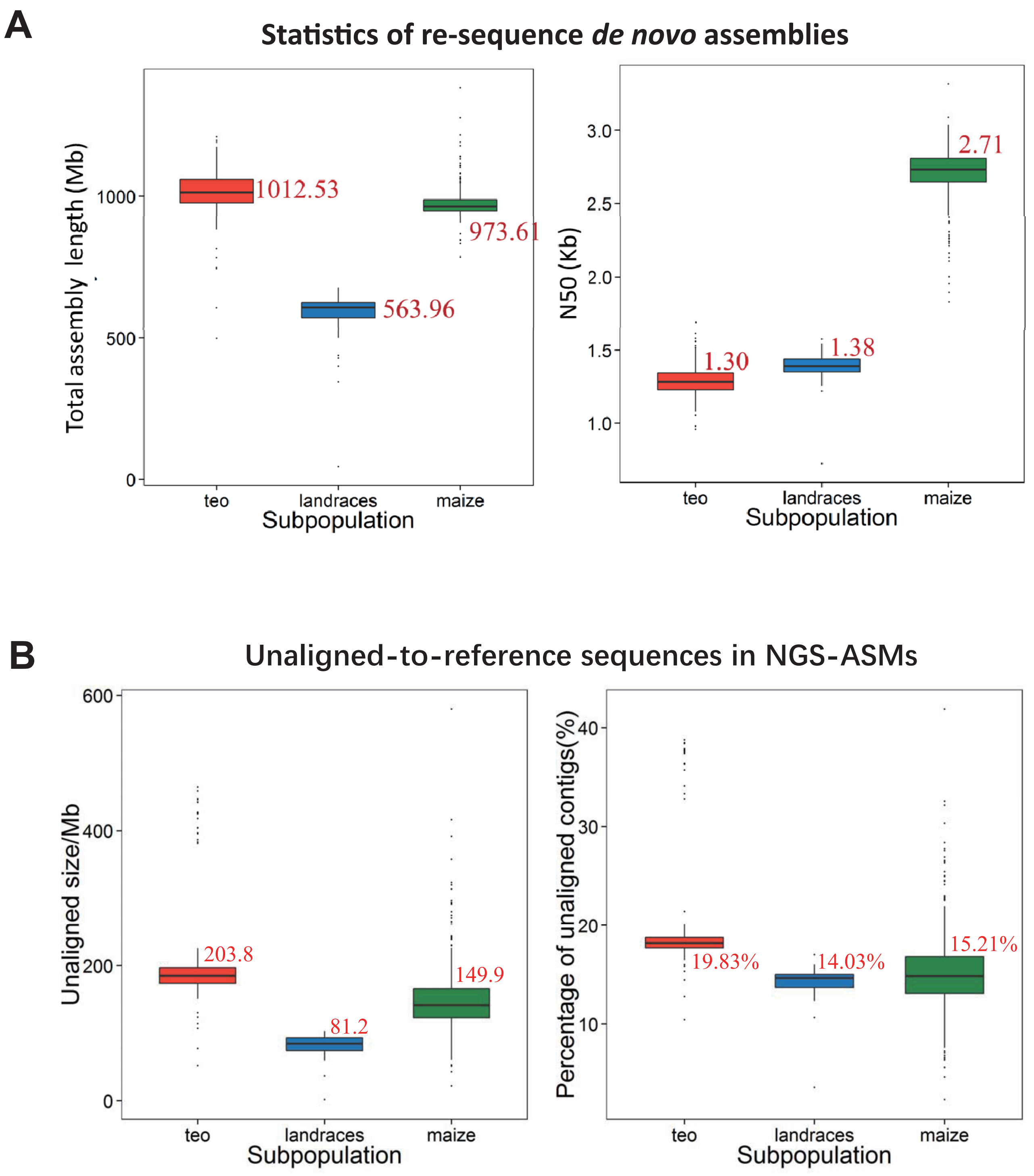

Figure S1. Statistics of pan-*Zea* NGS *de novo* assemblies.

(A): Distribution of total assembly length and contig N50 for each population. **(B):** Distribution of the unaligned to reference sequence total size and proportions. Red numbers indicated the mean values. “teo” indicated teosinte.

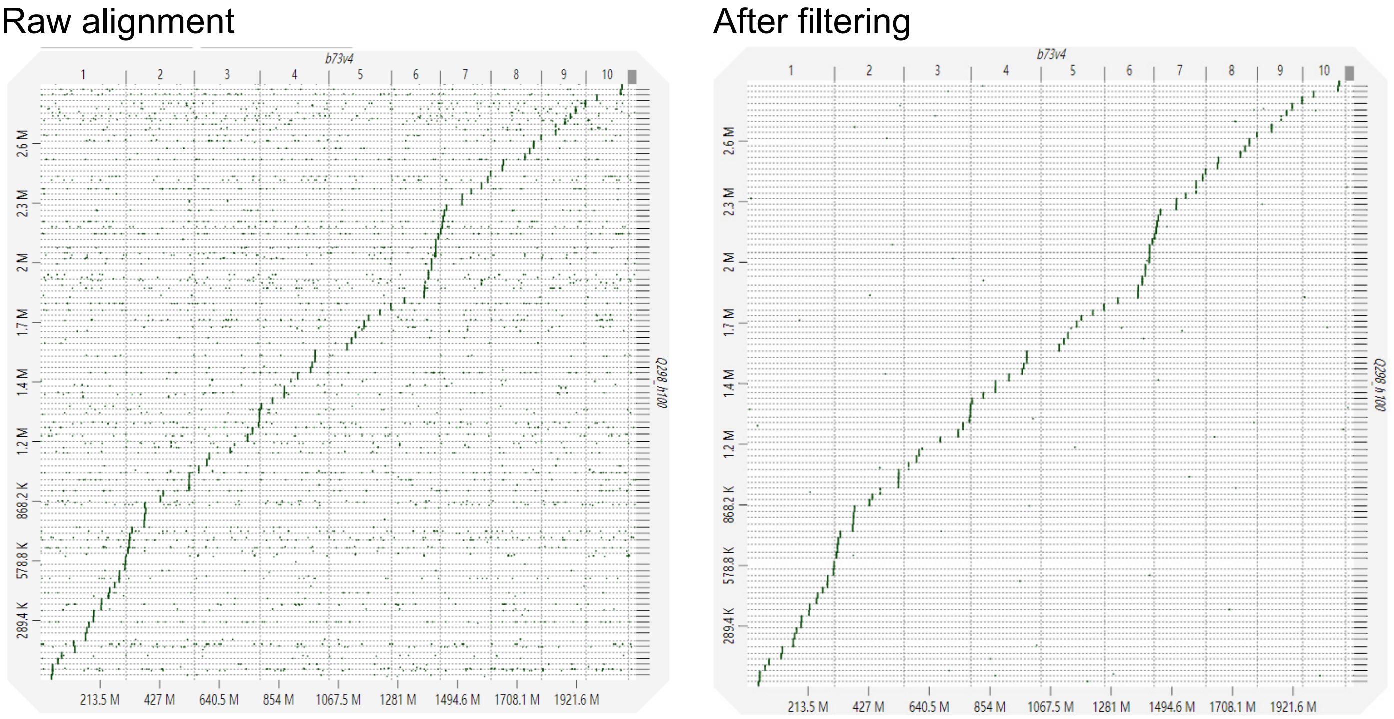

Figure S2. Schematic diagram illustrated the filtering of assembly-to-assembly alignment.

The plot showed the raw and filtered alignment between the top 100 contigs of maize inbred line JIAO51 and the B73 (AGPv4) reference genome. The filtering strategy clearly reduced the noise hits with the main hits retained.

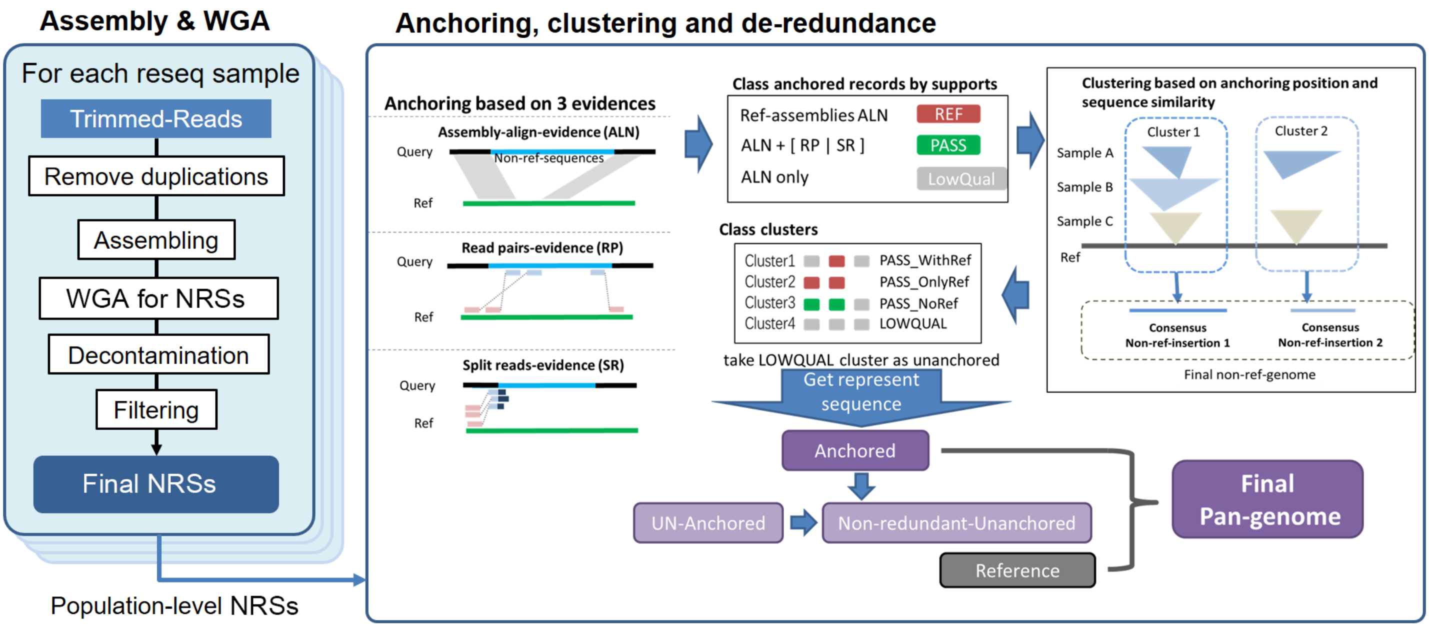

Figure S3. Sketch of the non-reference sequence anchoring and clustering pipeline.

The WGS reads of each inbred line were trimmed, de-duplicated and then assembled into contigs. The contigs were aligned to the reference genome to get the non-reference sequences (NRSs). The NRSs were filtered then anchored to the reference genome based on evidences of genome-to-genome alignment (ALN) and two short reads mapping evidences (RP and SR, for read pairs and split reads, respectively). The non-reference sequence was then classified into three classes based on the supported evidences. The sequences anchored to the same regions were clustered to get the non-redundant representations according to the non-reference sequence support classes. The un-anchored non-reference sequence were de-duplicated then combined with the anchored non-reference sequence to get the final non-reference sequence set.

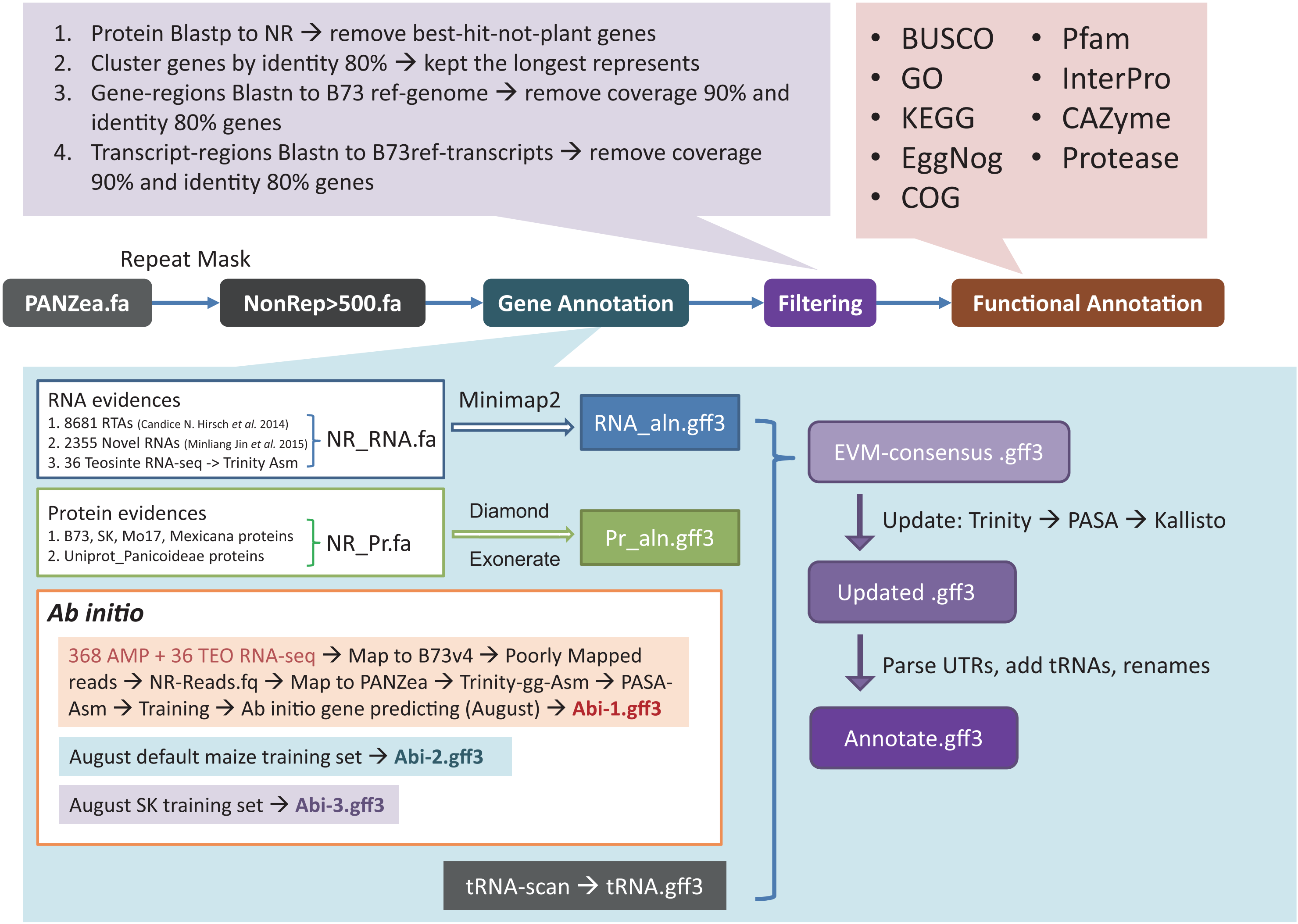

Figure S4. Pan-*Zea* gene annotation and functional annotation pipeline.
The non-reference genome was masked and annotated by combination of hybrid evidences and ab initio gene prediction. The predicted genes were filtered and mapped to common databases for functional annotation.

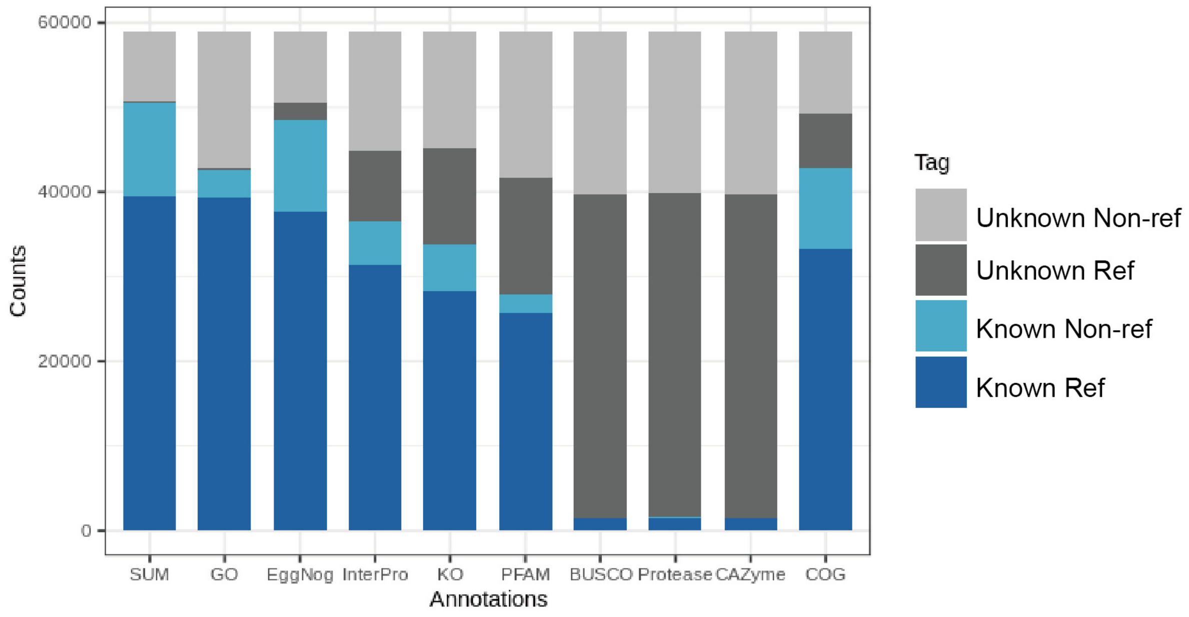

Figure S5. Statistics of the proportions of functionally annotated genes.

X axis indicated each functional annotation items while "SUM" represent for the summary of all functional annotations. Greys indicated genes without annotations on that item while blues indicated those with annotations.

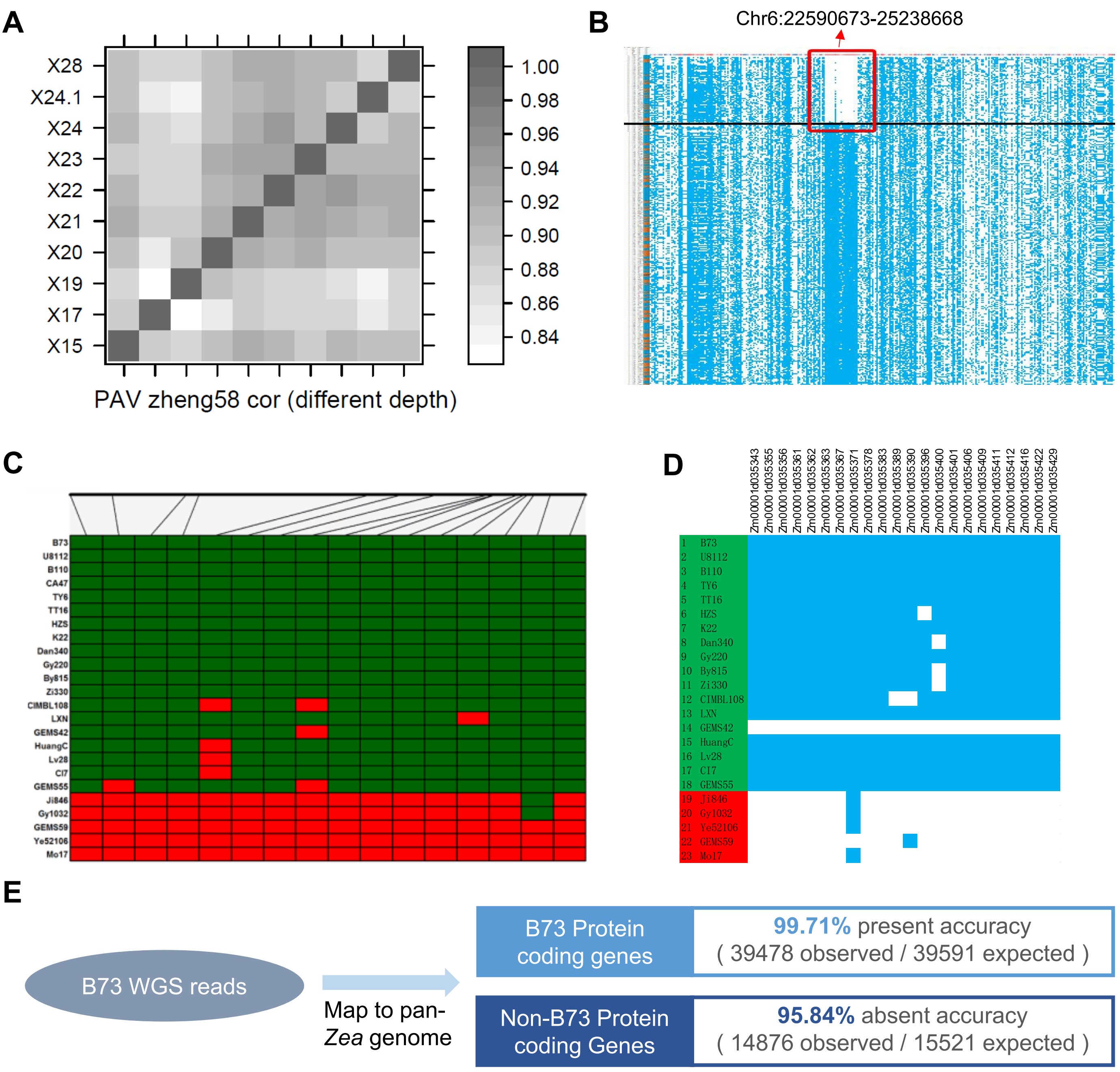

Figure S6. Validation of the gene present and absent variations (gPAVs).

(A): Heat map distribution of correlation r^2 values of the gPAVs of maize inbred ZHENG58 from NGS data of different read depths (X15-X28 in the Y-axis). **(B):** Schematic showed a continuous gene absent cluster on chromosome 6. Blue cells indicated gene presence. **(C):** The validation of a ~2.6 Mb deletion on chromosome 6 from PCR amplifications. Green indicated successful amplification for a particular inbred by primer combination while red indicates no amplification. Modified from the Supplementary Figure 5 of Yang et al. 2017 with permission. **(D):** The gPAV patterns of the genes located within the ~2.6 Mb deletion and of the same individuals used in the PCR validation in (C), with blue cells indicated gene presence. **(E):** The gPAV genotyping accuracy was estimated by the gPAV genotyping results of B73, which in expectation would have 100% reference genes present and 100% non-reference genes absent.

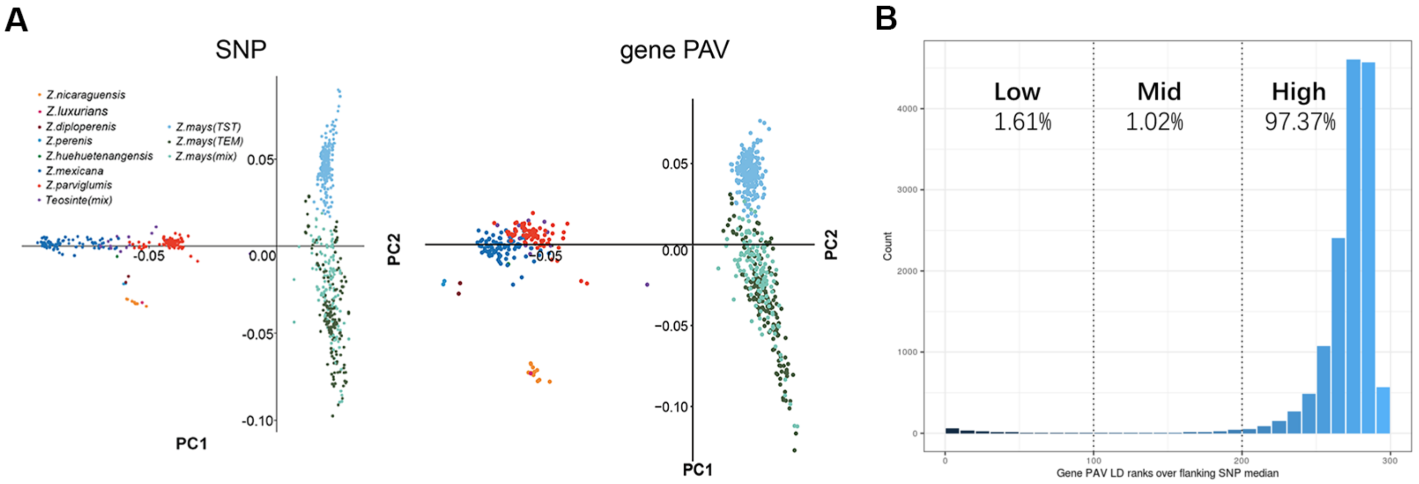

Figure S7. PCA and SNP LD rank analysis of gPAV.

(A): Comparison between the PCA result using pan-Zea SNPs and gPAVs. **(B):** Distribution of the number of gPAV r^2 ranks (0-300) that are above the SNP-based median r^2 value for common gPAVs.

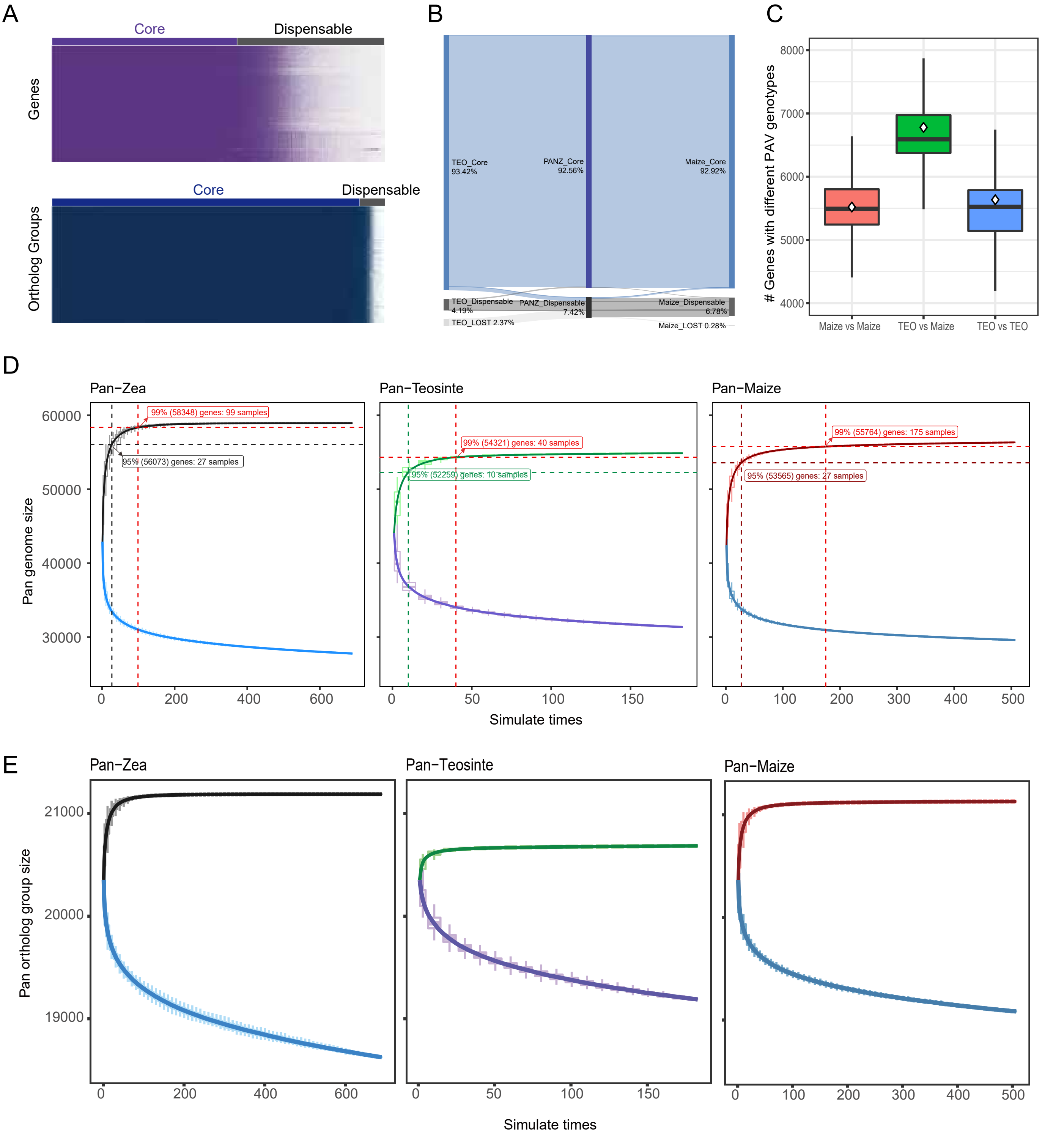

Figure S8. Core and dispensable genes and ortholog groups.

(A): Schematic of the present and absent (PAV) patterns of pan-*Zea* genes and ortholog groups. X and Y axes were genes (or ortholog groups) and individuals, respectively. The top-most grey bar indicated the dispensable items, while the top-most purple (or dark blue) bar indicated core or candidate core items. **(B):** Sankey plot of the proportions of the core and dispensable ortholog groups in pan-*Zea* (PANZ), teosinte sub-group (TEO) and maize sub-group. The “core” tracks represented the combinations of the core and candidate core ortholog groups. **(C):** Distribution of the pairwise comparison of the gPAV genotype differences between any two individuals. Comparison between the PCA result using pan-Zea SNPs and gPAVs. **(D and E):** Distributions of the size variation of pan and core genes **(D)** and ortholog groups **(E)** along with 100 times random addition of individuals for pan-Zea, pan-teosinte and pan-maize. X-axes indicated the total included individuals. The boxplots indicated the distributions of the sizes of the core or pan genes (or ortholog groups) in the 100 randomization. The lines were the connections of the mean values in each boxplot.

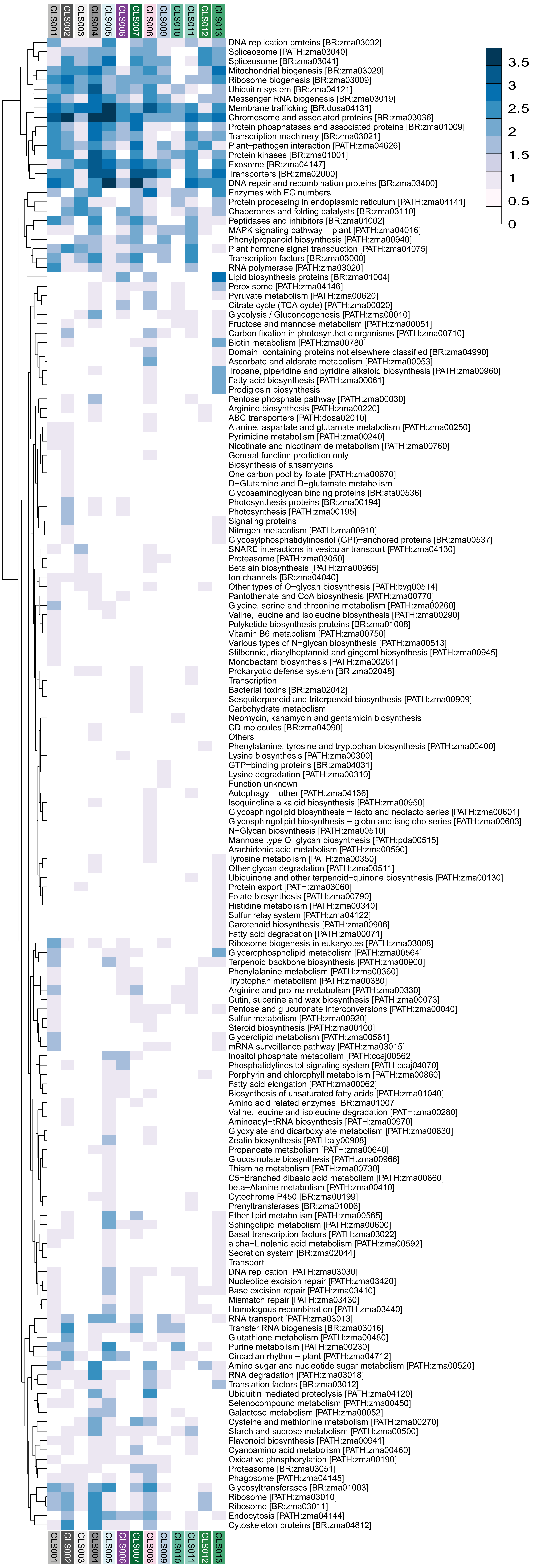

Figure S9. KEGG pathways of each dispensable ortholog group cluster.
The heat map plot indicated the KEGG pathways in each ortholog group cluster. The color gradient indicated the number of KOs related to the current pathway.

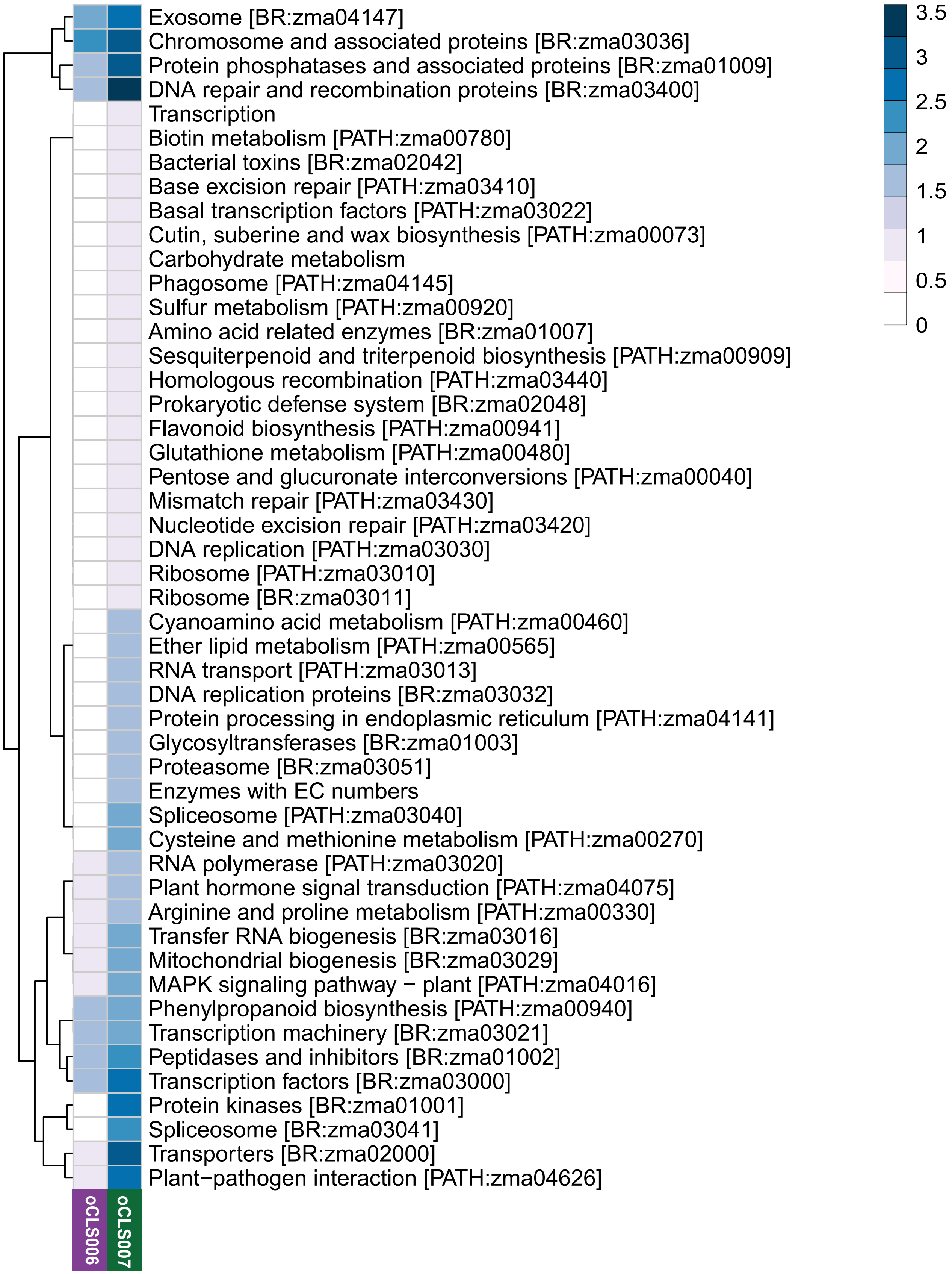

Figure S10. The KEGG pathways enriched in maize concentrated ortholog groups when compared with teosinte concentrated ortholog groups.

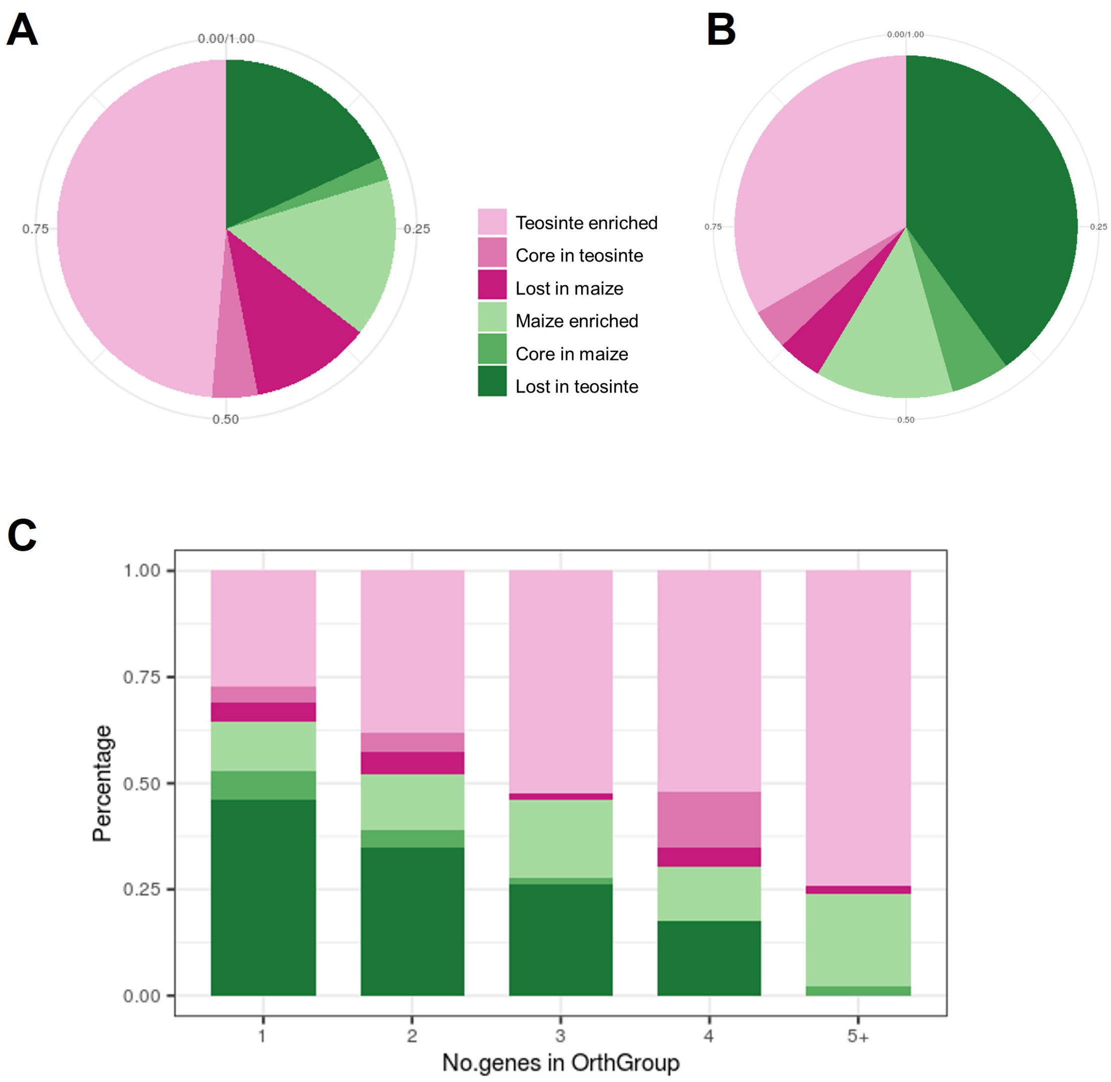

Figure S11. Distribution of sub-population enriched genes and ortholog groups.
(A) and (B): Pie plots of proportions of sub-population enriched genes (A) and ortholog groups (B). **(C):** Distribution of sub-population enriched ortholog groups along with the ortholog group size.

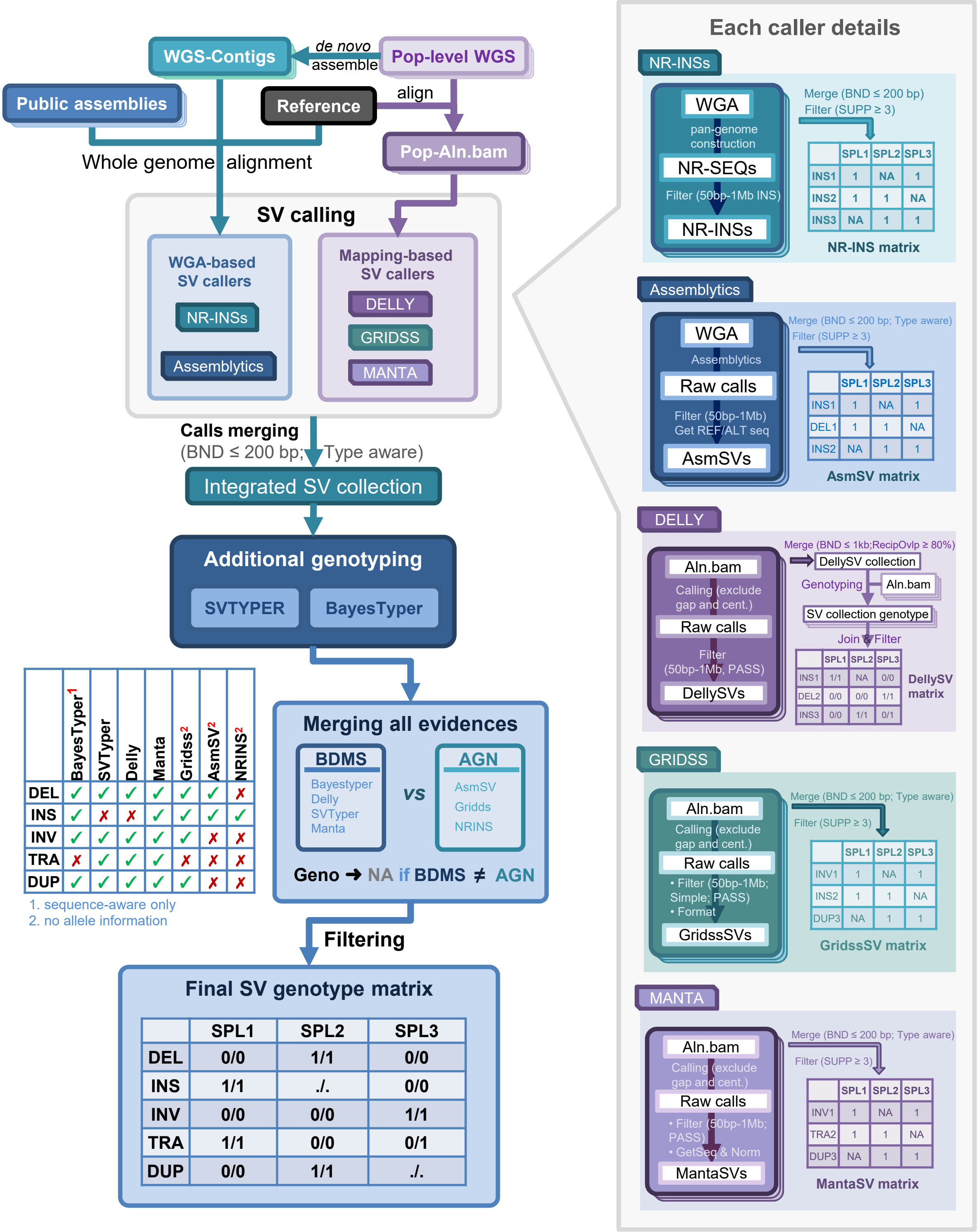

	BayesTyper[1]	SVTyper	Delly	Manta	Gridss[2]	AsmSV[2]	NRINS[2]
DEL	✓	✓	✓	✓	✓	✓	✗
INS	✓	✗	✗	✓	✓	✓	✓
INV	✓	✓	✓	✓	✓	✗	✗
TRA	✗	✓	✓	✓	✗	✗	✗
DUP	✓	✓	✓	✓	✓	✗	✗

	SPL1	SPL2	SPL3
DEL	0/0	1/1	0/0
INS	1/1	./.	0/0
INV	0/0	0/0	1/1
TRA	1/1	0/0	0/1
DUP	0/0	1/1	./.

Figure S12. The sketch of SV calling and genotyping pipeline.

The SVs were called from the combination of hits from the whole genome alignment (WGA) based evidences (NR-INSs and AsmSVs) and the next-generation whole genome sequencing (WGS) based SV callers (DELLY, GRIDSS, MANTA). Each of the SV callers was polished with both individual and population level filters. The final calls of each SV caller were integrated and additional joint SV genotyping steps were performed with SVTyper and Bayestyper. Finally, all the SV genotype evidences were merged and filtered to get the final SV genotype matrix. BND, break end; “Pop-“, population; aln, alignment; SPL, sample; RecipOvlp, reciprocal overlap; SUPP, number of samples supported the presence of a certain SV in the population; cent., centromere regions; PASS, the “PASS” filter tag in VCF4.0.

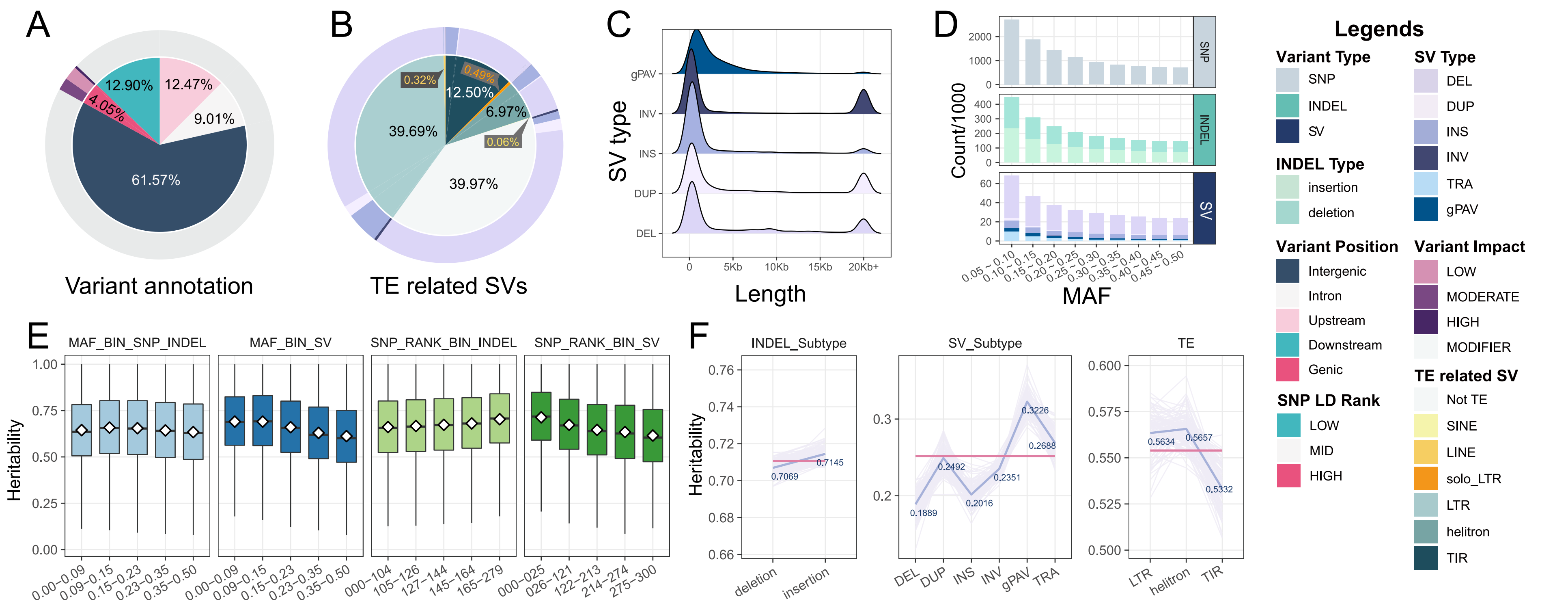

Figure S13. Additional features of the maize genetic variation map.

(A-B): Pie plots of the proportions annotations and relative position to the genes (A) and TE-related SVs (B). **(C):** Distribution of different SV sub-types and their lengths. Note that the length feature of the TRA type of SV was not available; thus, it was not provided in the plot. **(D):** Distributions of genetic variants along with MAF values. **(E):** Distribution of heritability and equal frequency MAF bins of the SNPs and InDels (light blue) SVs (blue), and SNP LD rank value bins of the InDels (light green) and SVs (green). **(F):** Trend lines of heritability among the different genetic variant features as supplementary of Figure 3F. Light purple lines indicate the feature item's mean heritability of all traits for each randomization. Blue lines indicate each feature item's mean heritability for all 100 randomizations. Red lines indicates the mean heritability of all of the feature items.

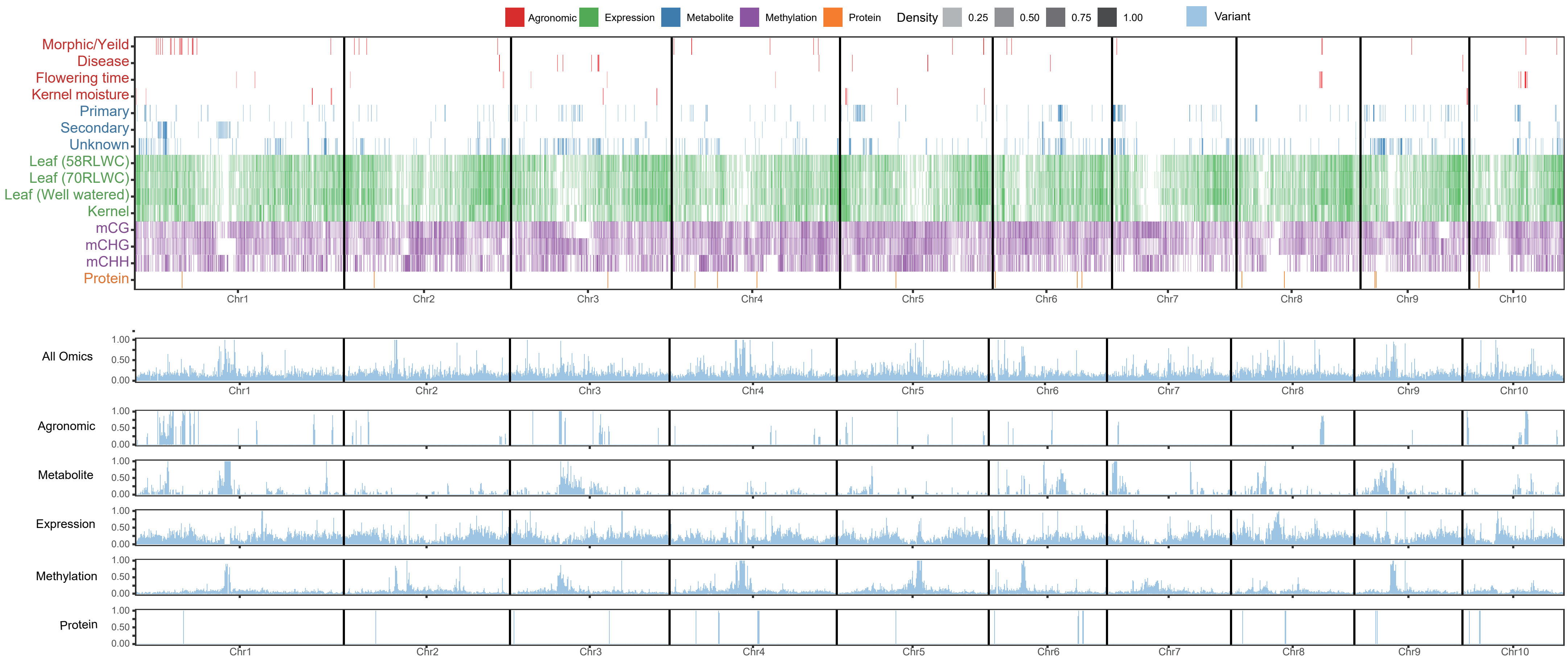

Figure S14. Distribution of the associated QTLs and causal variations along the chromosome.
The heatmap represents the QTL density within each 1Mb window. The histograms indicated the densities of the causal variants that were normalized with the number of all the variants within 1Mb window.

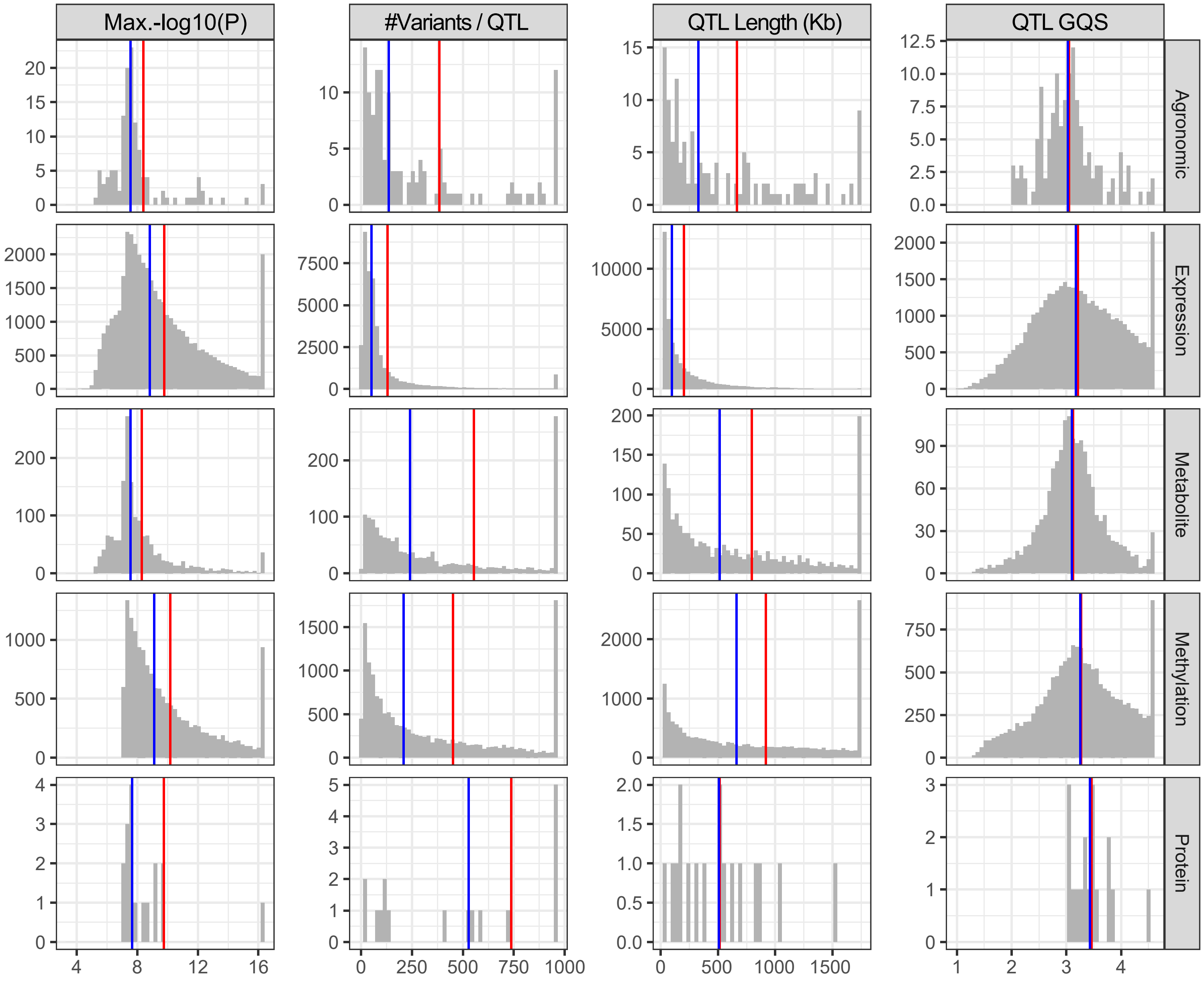

Figure S15. Distribution of the features of associated QTLs, genes and causal variations.
"Max.-log10(P)" indicated the negative log10 P-value of the leading genetic variation in the QTL. "QTL_GQS" indicated the QTL general quality score calculate using Manhattan Harvester. The blue and red vertical lines indicated the median and mean values, respectively.

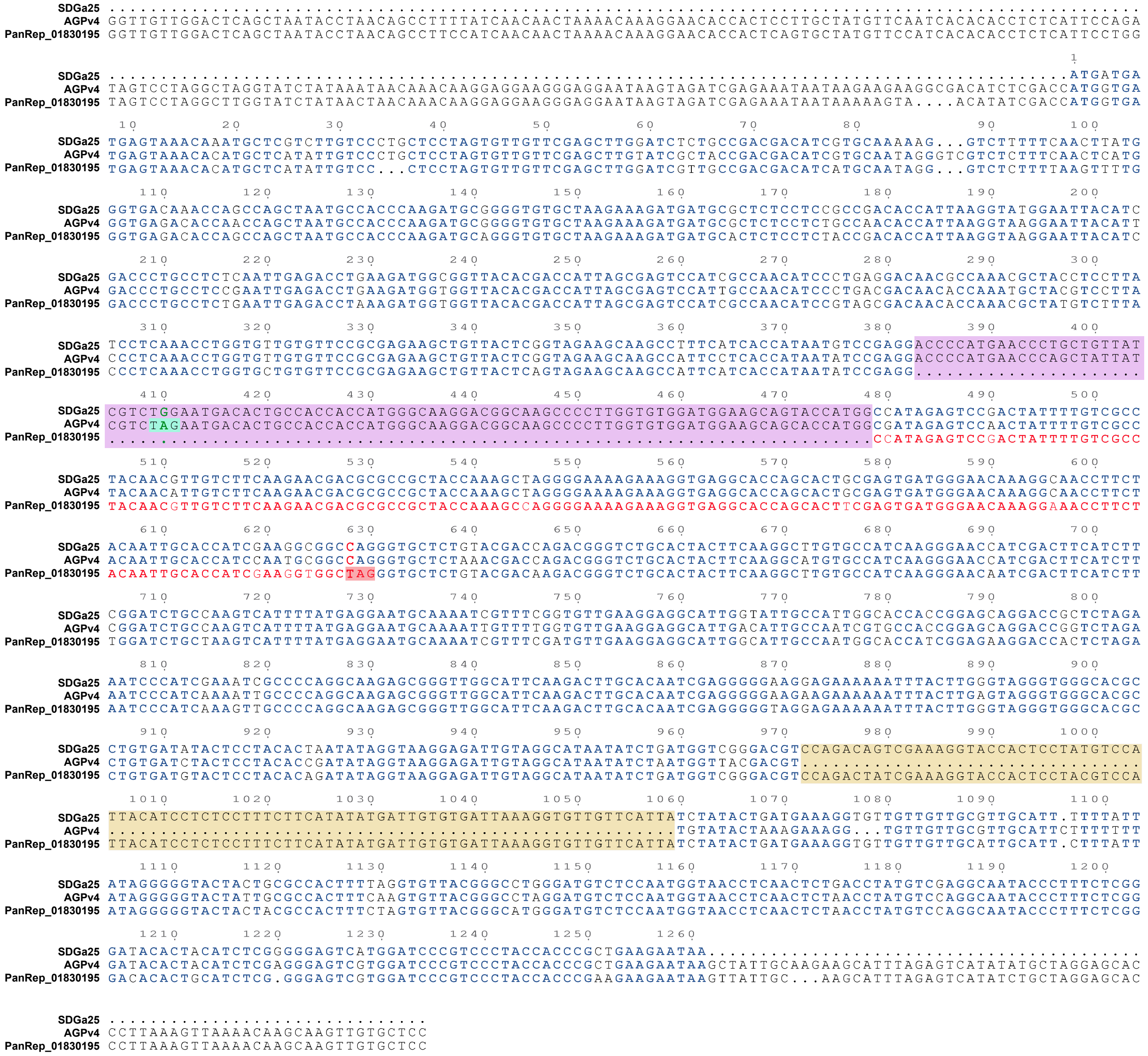

Figure S16. Multiple sequence alignment of the PME genes in the *Ga1* locus.

The base alignments represented the PME genes and their flanking sequences in SDGa25, AGPv4 and PanRep_01830196. The numeric ruler indicated the position of the intact PME gene in SDGa25. Conserved bases were highlighted as darkbule. The pre-mature mutations in AGPv4 and PanRep_01830196 were highlighted with green and red blocks, respectively. The deletions in AGPv4 and PanRep_01830196 when compared with SDGa25 were highlighted with yellow and purple blocks, respectively. The red bases indicated the additional coding sequence that presented in PanRep_01830196 but absent in AGPv4.

```
                       10        20        30        40        50        60        70        80        90       100
                   ....|....|....|....|....|....|....|....|....|....|....|....|....|....|....|....|....|....|....|....|
SDGa25             MMMSKQMLVLSLLLVLFELGSLPTTSCKK-VFFNLWVTNQPANATQDAGCAKKDDALSSADTIKVWNYIDPASQLRPEDGGYTTISESIANIPEDNAKRY
Zm00001d048936     MVMSKHMLILSLLLVLFELVSLPTTSCNRVVSFNSWVRHQPANATQDAGCAKKDDALSSANTIKVRNYIDPASELRPEDGGYTTISESIANIPDDNTKCY
PZ00001a032490     MFHHTPLIPGSPRLVLFELGSLPTTSCNR-VSFKFWVRHQPANATQDAGCAKKDDALSSTDTIKVRNYIDPASELRPKDGGYTTISESIANIRSDNTKRY
PZ00001a046864     -MNRRH-----------RDEALDETNAA------------PANATQDAGCAKKDDALSSADTIKVWNYIDPASQLRPEDGGYTTISESIANIPEDNAKRY
PZ00001a046460     MATREGGLSRQRRGQALGGGEVGALAAT------------PAGATRVWGEGRLSAGKRQGPPHR-GNYIDPASELRPEDGGYTTISESIANIPDDNTKRY
PZ00001a046478     MMMSKQMLVLSLLLVLFELGSLPTTSCKK-VFFNLWVTNQPANATQDAGCAKKDDALSSADTIKVWNYIDPASQLRPEDGGYTTISESIANIPEDNSKRY
PZ00001a047012     ----------------------------------------------------------------------------------------------------

                      110       120       130       140       150       160       170       180       190       200
                   ....|....|....|....|....|....|....|....|....|....|....|....|....|....|....|....|....|....|....|....|
SDGa25             LLILKPGVVFREKLLLGRSKPFITIMSEDPMNPAVIVWNDTATTMGKDGKPLGVDGSSTMAIESDYFVAYNVVFKNDAPLPKLGEKKGEAPALRVMGTKA
Zm00001d048936     VLTLKPGVVFREKLLLGRSKPFLTIISEDPMNPAIIV---------------------------------------------------------------
PZ00001a032490     VFTLKPGAVFREKLLLSRSKPFITIISE--------------------------------AIESDYFVAYNVVFKNDAPLPKPGEKKGEAPALRVMGTKE
PZ00001a046864     LLILKPGVVFREKLLLGRSKPFITIMS-------------------------------------------------------------------------
PZ00001a046460     VLILKPGVVFREKLLLGRTKPFITIIFEDPMNPVVIIWNDSATTMGKDDKPLGVDGSSTMAIESDYFVAYNVVFKNDAPLPKPGEKKGEAPALRVMGTKA
PZ00001a046478     LLILKPGVVFREKLLLGRSKPFITIMSEDPMNPAVIVWNDTATTMGKDGKPLGVDGSSTMAIESDYFVAYNVVFKNDAPLPKLGEKKGEAPALRVMGTKA
PZ00001a047012     ------------------------------MNPVVIVWNDTATTMGKDGKPLGVDGSSTMAIESDYFVAYNVVFKNDAPLPKLGEKKGEAPALRVMGTKA

                      210       220       230       240       250       260       270       280       290       300
                   ....|....|....|....|....|....|....|....|....|....|....|....|....|....|....|....|....|....|....|....|
SDGa25             TFYNCTIEGGQGALYDQTGLHYFKACAIKGTIDFIFGSAKSFYEECKIVSVLKEALVLPLAPPEQDRSRNPIEIAPGKSGLAFKTCTIEGEGEKIYLGRV
Zm00001d048936     ----------------------------------------------------------------------------------------------------
PZ00001a032490     TFYNCTIEGG------------------------------------------------------------------------------------------
PZ00001a046864     ----------------------------------------------------------------------------------------------------
PZ00001a046460     TFYNWTIEGGQGALYDQMGLHYFKACSIKGTIDFIFGSAKSFYEECKIISVLKEA----LAPPEQDRSRNPIKIAPGKSGLAFKTCTIEGEGEKIYLGRV
PZ00001a046478     TFYNCTIEGGQGALYDQTGLHYFKACAIKGTIDFIFGSAKSFYEECKIVSVLKEALVLPLAPPEQDRSRNPIEIAPGKSGLAFKTCTIEGEGEKIYLGRV
PZ00001a047012     TFYNCTIKGGQGALYDQTGLHYFKACAIKGTIDFIFGSAKSFYEECKIVSVLKEALVLPLAPPEQDRSRNPIEIAPGKSG--------------------

                      310       320       330       340       350       360       370       380
                   ....|....|....|....|....|....|....|....|....|....|....|....|....|....|....|....|
SDGa25             GTPVIYSYTNIGKEIVGIISDGRDVQTVERGYYCATFRCYGPGMSPMVTSTLTYVEAIPFLGIHYISGESWIPSLPPAEE
Zm00001d048936     --------------------------------------------------------------------------------
PZ00001a032490     --------------------------------------------------------------------------------
PZ00001a046864     --------------------------------------------------------------------------------
PZ00001a046460     GTPVIYSYTI----------------------------------------------------------------------
PZ00001a046478     GTPVIYSYTNIGKEIVGIISDGQDVQTVERGYYCATFRCYGPGMSPMVTSTLTYVEAIPFLGIHYISGESWIPSLPPAEE
PZ00001a047012     ------------------------------GYYCATFRCYGPGMSPMVTSTLTYVEAIPFLGIHYISGESWIPSLPPAEE
```

Figure S17. Multiple sequence alignments of the protein sequences of pan-*Zea* PME genes. The intact PME gene in SDGa25 was also included as a comparison.

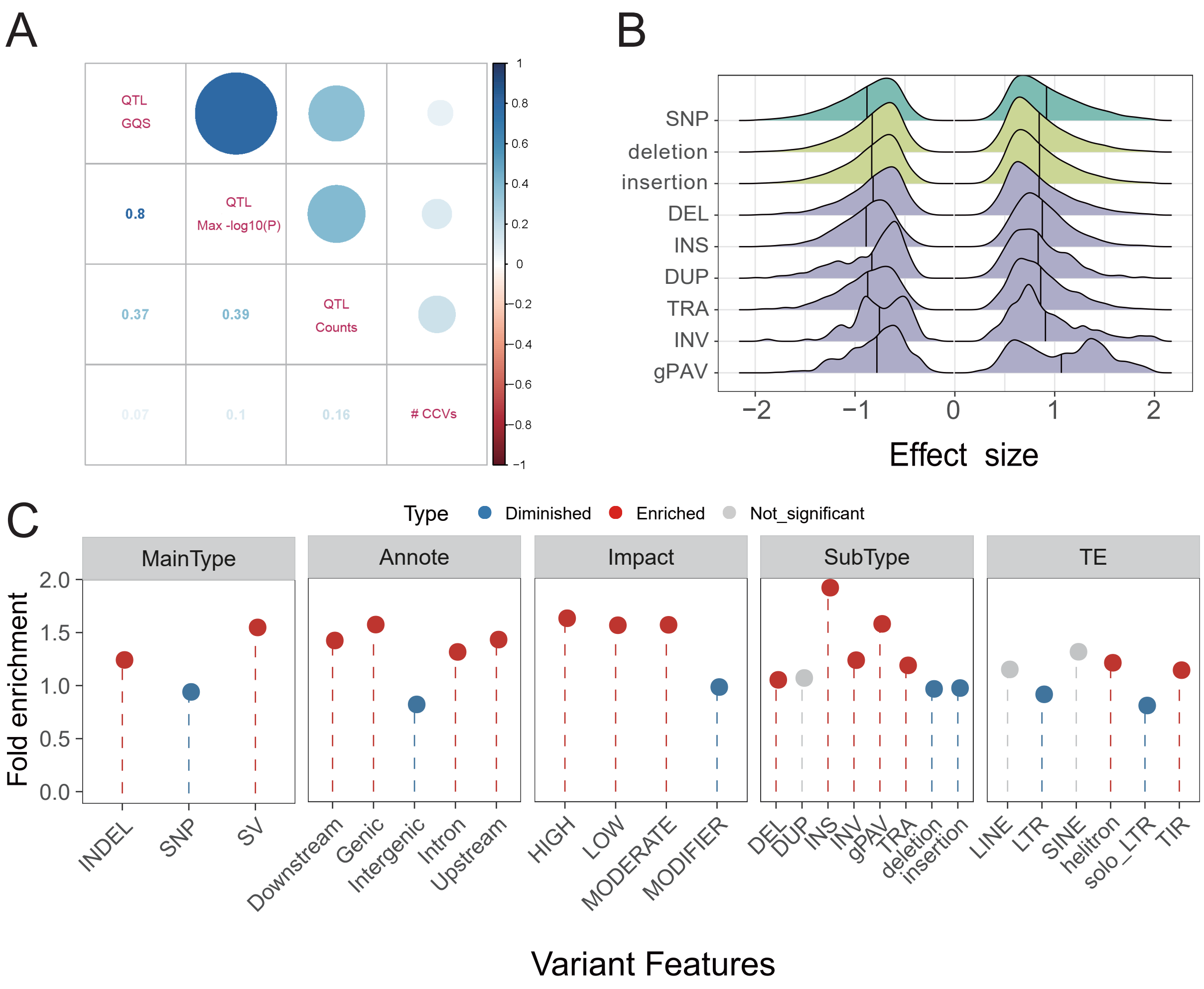

Figure S18. Statistics of candidate causal variants.

(A): Correlation of number of causal variant within a QTL with each QTL features. “QTL Counts”, the number of genetic variations within the QTL; “QTL Max -log10(P)”, the negative log10 P-value of the leading genetic variation in the QTL; “QTL GQS”, the QTL general quality score calculate using Manhattan Harvester; “#CCVs”, the number of candidate causal variants within the QTL. **(B):** Distribution of effect size along different type of causal variants, with the vertical lines indicated the mean values. **(C):** Enrichment of candidate causal variants under different variant features. Significant cutoff was set as Q value < 0.05.

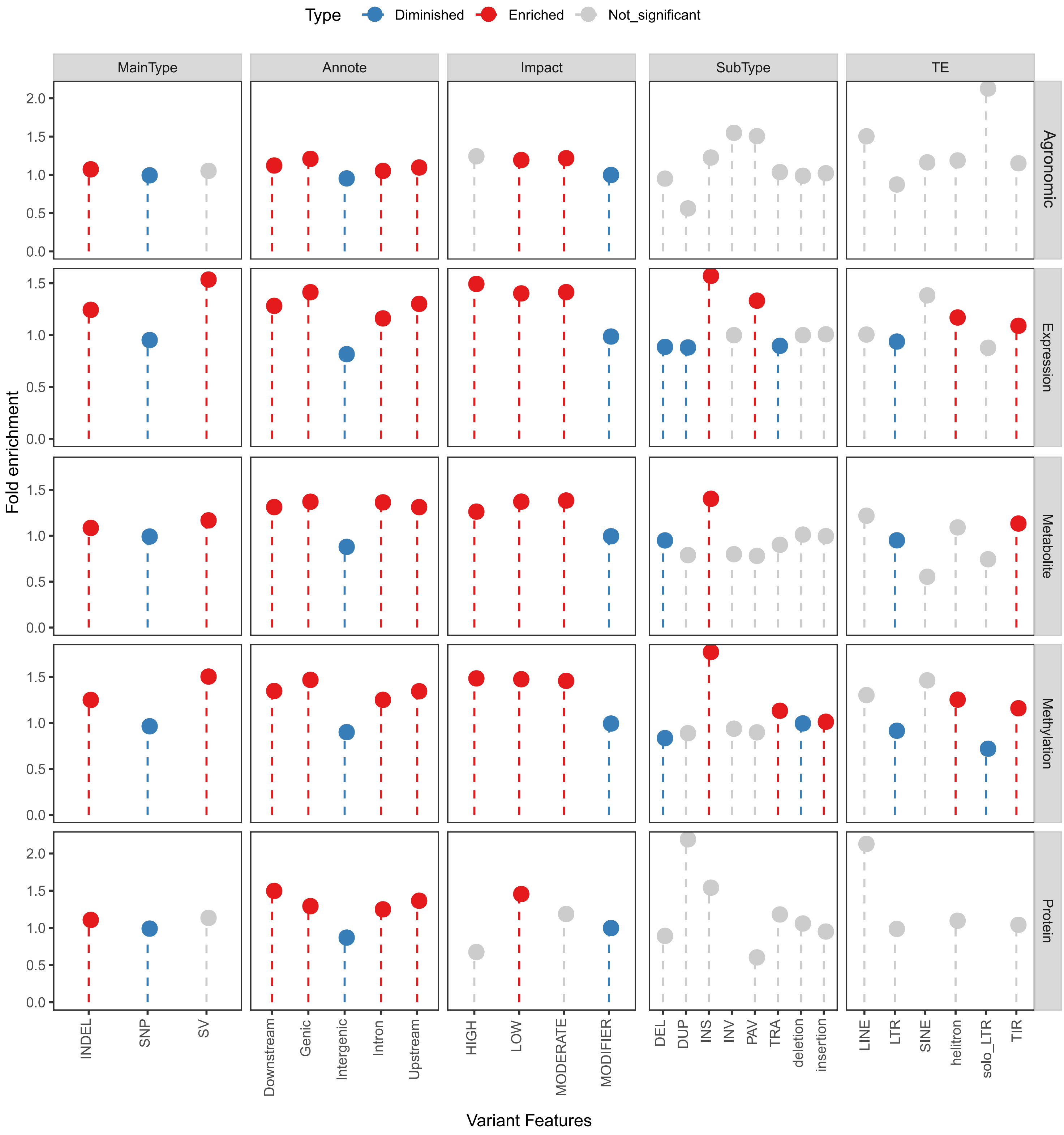

Figure S19. Enrichment of causal variants features within different omics trait classes. Significant cutoff was set as Q value < 0.05.

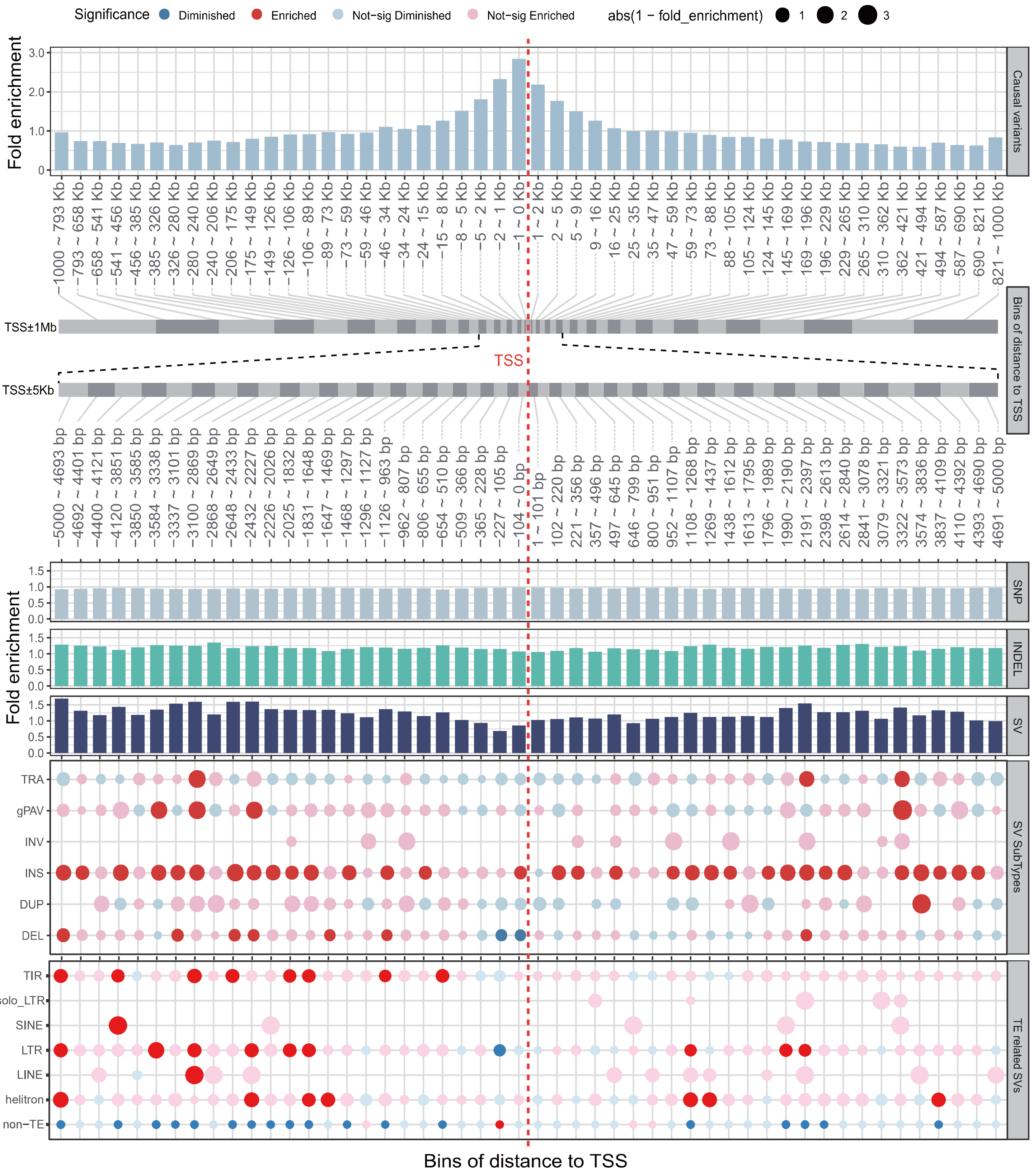

Figure S20. Enrichment of cis-eQTL causal variants along different distance to TSS.

Tracks from top to bottom: “Causal variants”, enrichment of all types of causal variants along 50 equal-frequency bins; “Bins of distance to TSS”, the equal-frequency binning of 50 bins along the flanking 1Mb and 5Kb of TSS, respectively; “SNP”, ”INDEL” and “SV”, the enrichment of causal SNPs, INDELs and SVs along 50 bins flanking 5Kb to TSS, respectively; “SV SubType”, enrichment of different SV subtypes along 50 bins flanking 5Kb to TSS; “TE related SVs”, enrichment of different TE types of TE-related SVs along 50 bins flanking 5Kb to TSS.

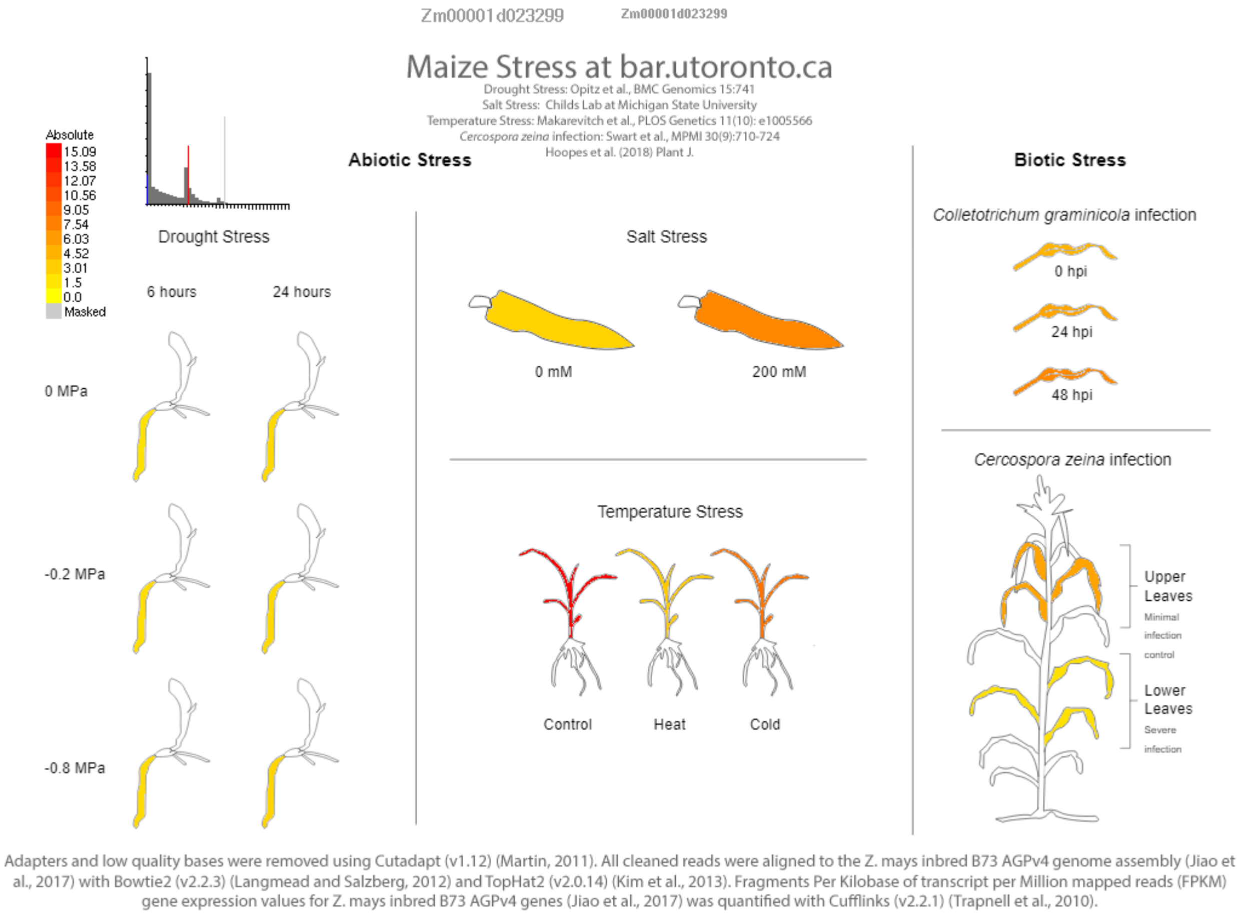

Figure S21. Expression patterns of the candidate genes of the example SV-QTLs.

The expression of Zm00001d023299 under different stress treatments. The results were acquired from the Maize eFP Browser (http://bar.utoronto.ca/efp_maize/cgi-bin/efpWeb.cgi).

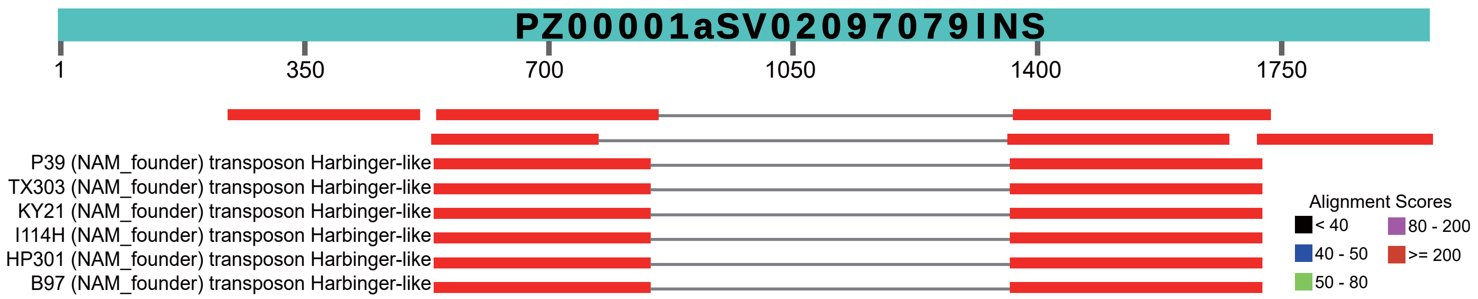

Figure S22. Blast graphic summary of PZ00001aSV02097079INS.

The PZ00001aSV02097079INS sequence was blasted against NCBI nt database with default options, only the top 18 hits were plotted.

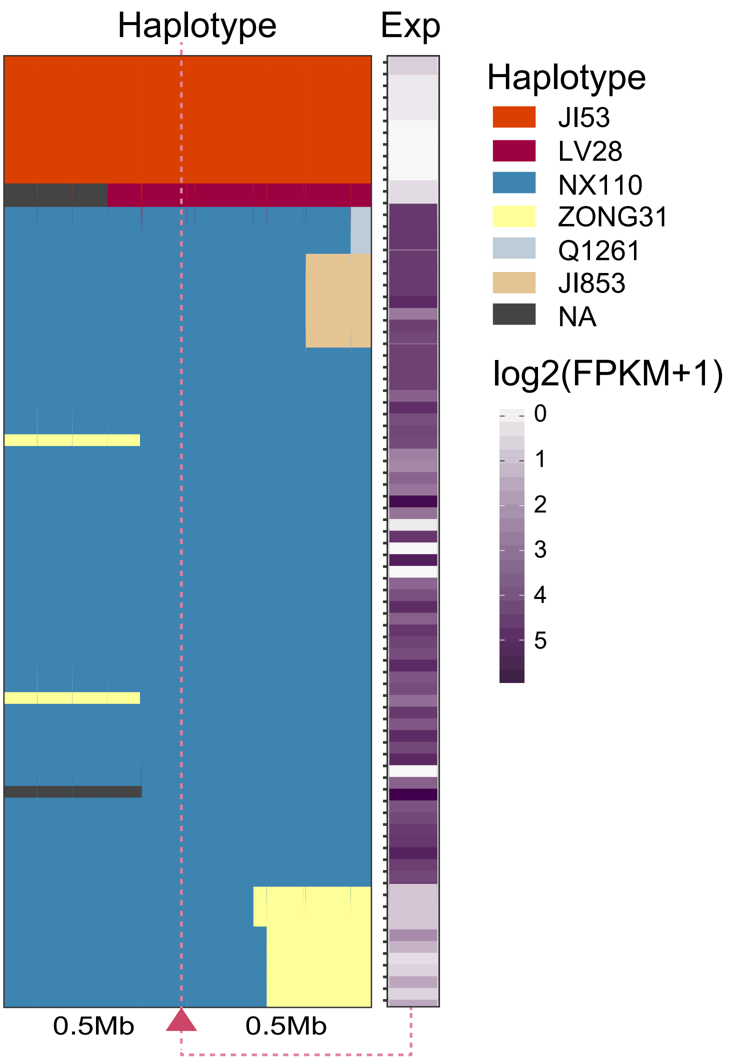

Figure S23. Distribution of the Zm00001d023299 expression patterns in the tenth leaf of V9 stage in the CUBIC offspring population related to the haplotypes flanking 500Kb of Zm00001d023299.
The haplotype of parent JI53 (light red) and LV28 (dark red) were the PZ00001aSV02097079INS containing haplotype, while the haplotype of NX110 (blue) was the PZ00001aSV02097079INS absent haplotype.

A

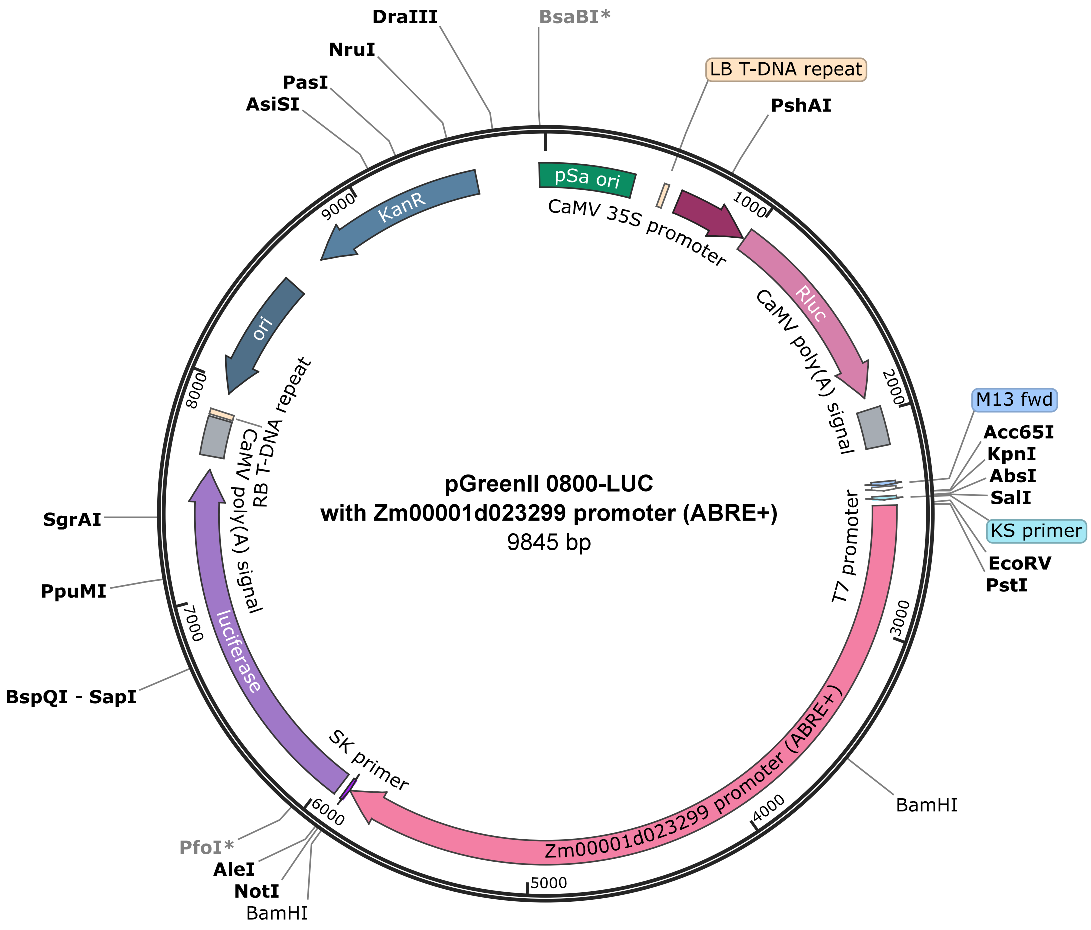

B

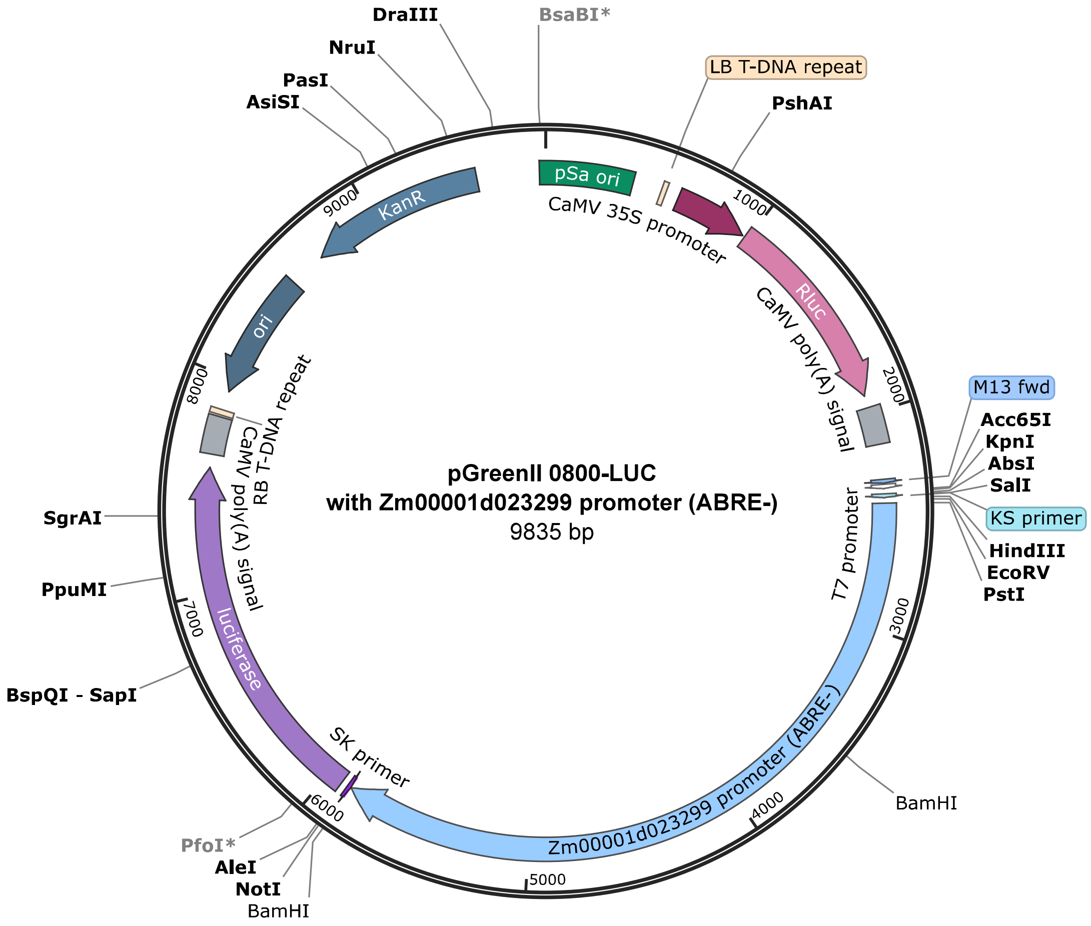

Figure S24. The diagram of vectors used in the luciferase reporter assays of Zm00001d023299 promoter with (A) or without (B) the predicted ABRE.
