# Supplementary material for: A pan-Zea genome map for enhancing maize improvement
Source: Genome Biol. 2022 Aug 23;23:178. doi: 10.1186/s13059-022-02742-7 (PMC9396798; doi:10.1186/s13059-022-02742-7)
Supplement: Supplementary file 11 — Additional file 11: Supplementary Text S1. [file 13059_2022_2742_MOESM11_ESM.pdf]

Supplementary Text S1

Construction and estimation of the pan *Zea* genome and pan *Zea* genes

A population that represents the full range of the genetic repertoire in the genus is required to construct a pan *Zea* genome. We collected whole-genome deep sequencing data (with an average depth of coverage greater than 20×) from 721 individuals, including 507 maize inbred lines from the maize association mapping panel (AMP) with tropical, subtropical, and temperate backgrounds and capable of representing global maize diversity [42], 31 landrace individuals representing six geographical regions that span the pre-Columbian range of maize cultivation [43], and 183 teosinte individuals covering all the seven teosinte subspecies [44]. The NGS reads of the teosintes, landraces and the maize AMP individuals were 150 bp pair-end, 100 bp pair-end, and 150 bp PCR-free pair-end, respectively. The NGS reads for each individual were cleaned and *de novo* assembled into contigs (hereafter referred to as NGS-ASMs) with average total lengths of 1,012,53 Mb, 563.96 Mb, and 973.61 Mb, and average contig N50 values of 1.30 Kb, 1.38 Kb, and 2.71 Kb, for the teosinte, landrace, and maize AMP individuals, respectively (Additional file 1: Fig. S1A). To estimate the quality and representativeness of the NGS-ASMs, we compared four NGS-ASMs (B73, Mo17, SK, and HZS) with their chromosome-level genome assemblies, which resulted in an average assembly accuracy of 98.83%, and the NGS-ASMs represented ~42.24% of their entire genome (~71.47% of the gene regions) (Additional file 2: Table S1). We also collected 10 chromosome-level maize genome assemblies and one teosinte draft assembly (hereafter referred to as CHR-ASMs) (Additional file 3: Table S2). The NGS-ASMs and CHR-ASMs were mapped and filtered against the maize B73 reference genome 4.0 (AGPv4) to obtain the assembly-to-assembly alignments (Additional file 1: Fig. S2 and Additional file 12: Supplementary Materials and Methods Section 2.2). The non-reference sequences (hereafter referred to as NRSs) were identified for each NGS-ASM and CHR-ASM, with average non-reference rates of 19.83%, 14.03%, and 15.21% for the teosinte, landrace, and maize individuals, respectively (Additional file 1: Fig. S1B). The NRSEQs were anchored to AGPv4 by merging evidence from the assembly-to-assembly alignments and the read-pair mapping, and redundant data were removed to obtain the final non-redundant NRS set (Additional file 1: Fig. S3 and Additional file 12: Supplementary Materials and Methods Section 2.3). In total, about 4,588.50 Mb of non-redundant NRSs were identified, and 58.86% (2700.75 Mb) could be anchored to the AGPv4 reference genome.

To estimate the representation of the pan-*Zea* NRs on maize population, we applied the pan-*Zea* genome constructing a pipeline to the 26 NAM founder genome assemblies [39], resulting in a NAM NRSEQ set containing 474,327 non-reference sequences with a mean length of 3,074 bp (range 100-100,956 bp) and a total size of 1,458,111,539 bp. The NAM NRSEQs and pan-*Zea* NRSEQs were compared using minimap2 with default options,

and the coverages of the mapped sequences (with criteria of at least 80% sequence were covered and with at least 80% identity) were collected as shown in Figure 1D.

To estimate the representation of the pan-*Zea* gene sets on the maize population, we have compared the 103,033 NAM pan-genes with the raw pan-Zea genes (those haven't been filtered according to the criteria in Section 3 of Additional file 12: Supplemental Materials and Methods, and Additional file 1: Fig. S4) with Blastp (hits with e-value < 1e-5 and at least 80% protein similarity and 80% either coverage was kept as matched). The results showed that ~81.08% (82,535/103,033) of the NAM pan-genes could be matched with the unfiltered pan-Zea genes (Supplementary Text Figure 1A). And ~6.97% of the NAM pan-gene hits were lost during the pan-Zea gene filtering procedure. This is a consequence we could bear, considering that the filtering procedure have also removed 96.36% low-quality gene annotations.

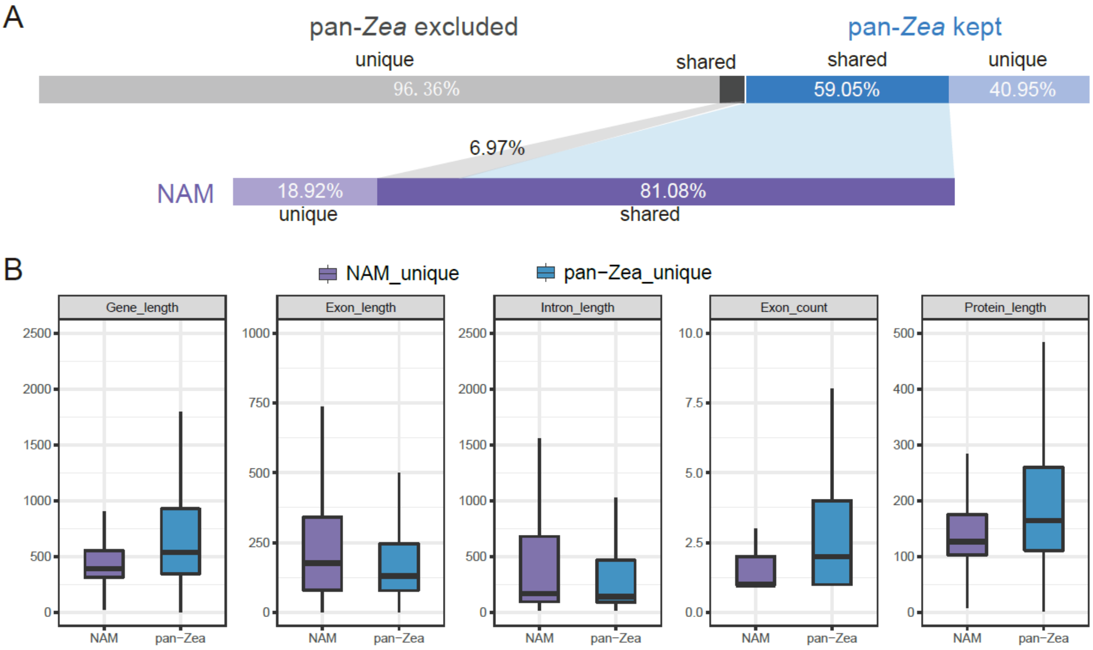

Supplementary Text Figure 1. Comparison between the pan-gene sets of NAM and pan-*Zea*. (A). Proportion of shared and unique genes between NAM pan genes and the unfiltered pan-*Zea* genes. pan-*Zea* exclude, the pan-*Zea* genes that were ultimately filtered out in the final pan-*Zea* gene set. pan-*Zea* kept, the final pan-*Zea* gene set. **(B).** Comparison of genic features between NAM unique genes and pan-*Zea* unique genes.
