# Supplementary material for: A pan-Zea genome map for enhancing maize improvement
Source: Genome Biol. 2022 Aug 23;23:178. doi: 10.1186/s13059-022-02742-7 (PMC9396798; doi:10.1186/s13059-022-02742-7)
Supplement: Supplementary file 12 — Additional file 12: Supplementary Materials and Methods [88–159]. [file 13059_2022_2742_MOESM12_ESM.pdf]

Supplementary Materials and Methods

1. Collection of genomic and transcriptomic data in pan-*Zea* individuals

The detailed information including the sequencing read sizes, geographic or pedigree information for the 721 pan-*Zea* individuals were listed in details from previous studies. Specifically, refer to [44] for the information of the 183 teosintes, refer to [43] for the 31 maize landraces, and refer to [20, 42, 44] for information of the 507 inbred lines in the maize association mapping panel (AMP). A copy of the sample information could also be accessed through Github via https://github.com/songtaogui/panz-paper-data/blob/master/05_samples_in_gwas/00_samples.tsv. The whole genome sequencing reads of the 507 inbred lines in the maize association mapping panel (AMP) were retrieved from the previously published study from GenBank with Bioproject accession PRJNA531553. The whole genome sequencing reads of the 183 teosinte lines were retrieved from PRJNA641489. The whole genome sequencing reads of the 31 landraces are retrieved from PRJNA300309. The accessions of the 11 publicly available genome assemblies were listed in Additional file 2: Table S1. The non-reference pan-maize transcript sequences were retrieved from previously published studies [40, 41]. The RNA sequencing data of the kernels of the AMP inbred lines and the RNA sequencing data of the leaves of 16 teosintes were retrieved from GenBank with accessions PRJNA208608 and PRJNA645739, respectively. The genome assemblies of the NAM founders were downloaded from MaizeGDB (https://www.maizegdb.org/NAM_project).

2. *De novo* assembly, whole genome comparison and pan genome construction

2.1 *De novo* assembly of each WGS samples

The optical duplicate sequences of each sample's WGS clean reads were removed using clumpify.sh from BBTools version 38.42 [88]. The clean reads were then assembled to scaftigs using IDBA-UD [89] with parameters of "--mink 20 --maxk 100 --step 20", and the resulting scaftigs longer than 500 bp were kept for downstream analysis. The assembly quality was evaluated by aligning the four assemblies which have reference genomes to their reference sequences using Quast version 5.0.2 [90] with parameters of "--debug --eukaryote --fragmented --no-snps --no-plots --no-icarus --no-sv".

2.2 Whole genome comparison to get non-reference sequences

To get the non-reference sequences (hereafter referred to as NRSs) for each sample, each *de novo* assembly was mapped to the maize B73 reference genome 4.0 (AGPv4) using minimap2 version 2.17-r941 [91] with parameters of "-c -x asm5 -B5 -O4,16 --no-long-join -r 85 -N 50 -s 65 -z 200 --mask-level 0.9 --min-occ 200 -g 2500 --score-N 2 --cs" (For reference level assemblies, the sequences were split into scaftigs before mapping). The alignments were filtered according to the idea of Quast version 5.0.2 [90] which kept the best alignment for each contig to minimize the variants . We took a sequence as candidate non-reference if (a) it is unaligned in the filtered alignment result, (b) its length ≥ 100 bp and (c) its belonging scaftig has at least one unaligned fragment ≥ 500 bp. The resulted candidate NRSs were then BLAST (blastn version 2.9.0, with options of "-task megablast -max_target_seqs 10 -evalue 1e-5 -perc_identity 0.5") against NCBI GenBank non-redundant nucleotide database (updated date: July 23rd, 2019) and removed the best-hit-not-plant records, the resulted sequences were then re-aligned to reference using BWA MEM [92] with default options and records with coverage > 80% and identity > 90% were filtered out to get the final NRSs.

2.3 Anchoring and clustering of non-reference sequences to get the final non-reference sequence set

Three criteria were considered in order to access the physical location of NRS: (i) the alignment information of the flanking sequences of the NRS (hereafter referred to as anchor-ALN), which was retrieved from the aforementioned assembly-to-reference alignment results, and (ii) read pairs or (iii) split reads evidences (hereafter reference to as anchor-RP and anchor-SR, respectively), which were generated by parsing the result of PopIns version damp_v1-151-g4010f61 [93] (See available codes for the details). For the 11 publicly available assemblies, each NRS that with left or right position anchored to AGPv4 were marked as "REF". For the WGS-scaftig level assemblies, each NRS were marked as "PASS" if its left or right position has anchor-ALN evidence and at least one of anchor-RP and anchor-SR evidences,

and the NRS with only anchor-ALN evidence supported were marked as "LowQual" (Additional file 1: Fig. S3).

To get the final non redundant NRS set, the anchored NRSs were clustered by their anchoring position flanking 200 bp. For each cluster, the cluster was token as anchored if it fulfills one of the criteria: (i) at least one NRSs in the cluster was marked as "REF", or (ii) at least two NRSs were marked as "PASS". Clusters with only "LowQual" NRSs were considered as unanchored (Additional file 1: Fig. S3). For each anchored cluster, the NRSs were filtered by removing redundancies using CD-HIT version 4.8.1 [94] with options "cd-hit-est -aS 0.8 -c 0.9 -n 10-d 0 -G 0". The representation of each CD-HIT group was chosen according to the priority of (i) the longest, (ii) both end anchored, (iii) marked as "REF" and (iv) marked as "PASS". The non-redundant anchored NRS set was generated by collecting the representation of all CD-HIT groups. For the unanchored NRSs, all the unanchored NRSs were mapped to the non-redundant anchored NRS set and filtered out records with coverage > 80% and identity > 90%. The remaining unanchored NRSs were then filtered using CD-HIT (same options as in anchored cluster) by taking the longest as representation. The final non-redundant NRS set was the combination of the non-redundant anchored and unanchored NRS sets.

3. Pan-genome gene annotation

To predict the gene models of the non-reference genome, the genome sequences were softly masked using RepeatMasker version open-4.0.9 (with options "-cutoff 250 -nolow -no_is -norna", against the Repbase and Dfam Consensus release 20181026) and RepeatModeler version 1.0.8 (http://repeatmasker.org), and only records with more than 500 bp non repeat sequence were passed to the downstream annotation pipeline.

The non-reference genome was annotated by combination of hybrid evidences and *ab initio* gene prediction using the funannotate pipeline version 1.5.3-21ad095 (https://github.com/nextgenusfs/funannotate). For transcript evidences, we have collected the 8,681 novel representative transcript assemblies (RTAs) and the 2,355 novel RNAs from previous maize pan transcriptome studies [40, 41]. Besides, we have *de novo* assembled the leaf RNA-sequencing data of 16 teonsintes using Trinity version 2.8.3 [95] with default options. All the three RNA sequence datasets were merged into the non-redundant RNA sequences and mapped to the non-reference genome as the transcript evidences. For protein evidences, we have combined the predicted proteins of three maize genomes and one teosinte genome (Additional file 1: Fig. S4) with the Panicoideae proteins in Uniprot version 2019_04 [96] to get the non-redundant proteins. The protein sequences were then mapped to the non-reference genome using Diamond version v0.8.22.84 (with options" diamond blastp -sensitive --evalue 1e-05 --max-target-seqs 1") [97] and exonerate version 2.2.0 [98] as the protein-based annotation evidence.

For *ab initio* prediction, we firstly mapped the population level RNA-sequence reads (368 inbred lines of maize association mapping panel and 16 teosinte lines) to the AGPv4 reference genome and get the non-redundant poorly mapped reads. The non-redundant poorly mapped reads were then mapped to the non-reference genome and assembled using a genome guide pipeline with Trinity. An evidence-driven prediction procedure were performed with the assembled transcripts using PASA version 2.3.3 [99], and Kallisto version 0.44.0 [100] was used to determine which PASA gene models to select at each locus. The resulting PASA gene models were then used to train the *ab initio* gene predictors with AUGUSTUS version 3.3.2 [101], two additional gene predictors using the default maize training set in AUGUSTUS and the training set of a tropical maize inbred line SK [20] were also used for gene prediction. The tRNA genes were predicted using tRNA-scan version 2.0 [102]. We then used Evidence Modeler version 0.1.3 [103] to generate the consensus gene models. Finally, an additional PASA and Kalisto step were performed to update the gene models. The updated gene models were then filtered with the following criteria to get the final gene annotations: (i) the predicted proteins were aligned with the GenBank non-redundant protein database (updated date: July 23rd, 2019) using BLASTP version 2.9.0 with default options, and the best-hit-not-plant genes were excluded, then (ii) the remaining genes were clustered with identify ≥ 80% and the longest representation of each cluster was kept, and (iii) the whole gene region sequences were blastn against the AGPv4 reference genome and the records with mapping coverage ≥ 90% and identity ≥ 80% were removed, finally, (iv) the transcript region sequences were blastn against the transcripts of AGPv4 reference genome, and the records with mapping coverage ≥ 90% and identity ≥ 80% were removed. The final pan *Zea* gene models were the combination of the non-reference gene models and the AGPv4.43 reference maize gene models.

The functional annotation information was added to the final pan *Zea* gene models using the previously described pipelines [57]. In general, the protein sequences of the predicted genes were compared against the InterPro database version 74.0 using InterProScan 5 [104] to identify functional protein domains. The proteins were further compared against the GenBank non-redundant protein database using BLASTP with the options "-p blastp –e 1e-05 –b 5 –v 5 –a 4 –m 7" and further analyzed by Blast2GO [105] to assign gene ontology (GO) terms. Kyoto Encyclopedia of Genes and Genomes (KEGG) annotations were performed by running BLAST against the KEGG database (version 84.0) with options "-p blastp -e 1e-05 -a 4 -m 8". The proteins were also searched against PFAM version 32.0 [106] using HMMer version 3.1b2 [107] with default parameters. The gene orthologs and clusters of orthologous groups (COGs) were annotated by mapping genes to EggNOG orthology database (version 4.5.1) [108] using emapper-1.0.3 [109]. To add gene-product annotations, the proteins were searched against UniProt database (version 2019_04) [96] using Diamond

with the options "--evalue 1e-05 --max-target-seqs 1", the UniProt and EggNog search results were combined to get the gene and product names using Gene2Product v1.32 (https://github.com/nextgenusfs/gene2product). Possible proteolytic enzymes were annotated by searching the proteins against the MEROPS database (version 12.0) [110] using Diamond with the options "--evalue 1e-05 --max-target-seqs 1". The proteins were also searched against the embryophyta single copy ortholog models from BUSCO Datasets (embryophyta_odb9, updated date: February 13th, 2017) [111] using HMMer with default options.

4. Pan *Zea* gene and ortholog group analyses

4.1 Gene and ortholog group PAVs

To call the gene presence and absence matrix, the raw WGS reads of each inbred line were mapped to the pan *Zea* genome using BWA mem with default options. The gene PAV information was parsed from the mapping bam file using SGSGeneLoss version 0.1 [112] with options "minCov=2 lostCutoff=0.2". The ortholog group PAV matrix were inferred from the gene PAV matrix using EUPAN gFamExist [113]. Because of the shorter total length of the draft assemblies and the shorter reads length of the 31 landrace individuals when compared with the maize and teosinte individuals, which may have potential bias, the landrace individuals were not included in the gPAV calling and the downstream analyses.

4.2 Determination of core and dispensable genes (or ortholog groups), and estimation of pan gene (or ortholog groups) set size

We defined the "core gene (or ortholog group)" as a gene (or ortholog group) with loss rate not significantly larger than 0.01. An exact binomial test on (present, loss) matrix with H_0 of loss_rate < 0.01. The significant P value was calculated with the binom.test function in R version 3.6.0 [114]. The genes (or ortholog groups) with P values ≤ 0.05 were regarded as "dispensable", while the genes (or ortholog groups) that with P values > 0.05 were regarded as "core".

To estimate the pan gene (or ortholog group) set sizes, the pan-*Zea*, pan-maize and pan-teosinte inbred lines were randomly added one by one (repeated 100 times), the pan gene (or ortholog group) sizes and the core gene (or ortholog group) sizes were recorded for the including of each new inbred line. The analyses were performed using eupan sim with options "-n 100".

4.3 PAV patterns among different subgroup

To cluster the genes (or ortholog groups) based on PAV patterns, we reduced the dimensions of the PAV matrices by constructing accurately approximated K-nearest neighbor graphs from the PAV matrices and then arranged the graphs in two-dimensional spaces using LargeVis [115]. The two-dimensional representations of the PAV matrices were then clustered using the K-means clustering algorithm. The number of clusters (k) were determined by calculating the within cluster sum of squared errors (WSS) for different values of k. Instead of choosing the k based on the elbow point in the plot of WSS versus k (which were usually 2 or 3 and represented the core and dispensable features of the genes), we chose the k as the point that first reached the plateau of the WSS *vs* k plot.

To further investigate the genes that showed significant maize-teosinte or TST-TEM PAV differences, the PAV patterns of each gene between two populations were compared using two-sided Fisher's exact test, and the genes with FPR corrected P values < 0.05 were considered significantly different distributed between the two populations. The dispensable genes that did not show significant enrichment P values in either maize-teosinte or TST-TEM comparisons were marked as "RANDOM".

5. Characterizing gene features

5.1 Gene and ortholog group age

The age of each gene was inferred using method similar with those in previously studies [16, 116]. Generally, the proteins in the GenBank non-redundant protein (nr) database were grouped into 23 taxonomic levels (P01: Cellular, P02: Eukaryota, P03: Viridiplantae, P04: Streptophyta, P05: Streptophytina, P06: Embryophyta, P07: Tracheophyta, P08: Euphyllophyta, P09: Spermatophyta, P10: Magnoliopsida, P11: Mesangiospermae, P12: Liliopsida, P13: Petrosaviidae, P14: Commelinids, P15: Poales, P16: Poaceae, P17: PACMAD clade, P18: Panicoideae, P19: Andropogonodae, P20: Andropogoneae, P21: Tripsacinae, P22: Zea, P23: Zea mays) based on NCBI taxonomy IDs. All the pan-genome proteins were aligned to the NR database using diamond with options of "blastp --evalue 1e-5 –id 0.3". The age of a

gene was considered as the taxonomic level of the oldest aligned protein. Genes that failed to align to the database were assigned to taxonomic level P23. The age of an ortholog group was considered as the age of the oldest gene within the ortholog group. The procedures were embedded in custom script “PANZ_gene_age.sh”.

5.2 Gene dN/dS and Tajima’s D

The dN/dS and Tajima’s D were calculated by comparing the variations between teosinte lines and maize lines. Genes that presented in less than four inbred lines of either teosinte or maize group were excluded. The longest transcript isoform of each gene was used as the representation of that gene, and only genes that show differences in at least one CDS region between the two groups were included. The consensus gene sequences for each inbred line were generated based on the SNPs (see section 6 below for the details of genetic variant calling and genotyping) using bcftools version 1.9 [117] with options “consensus –H 2”, and the stop codon was removed. The dN/dS for each gene was calculated using SNPGenie [118] with a sliding window approach (window size: 5 codons, sliding step: 1 codon) and a bootstrap number of 100 to calculate the standard error of dN-dS. The Tajima’s D for each gene was calculated using vcftools version 0.1.16 [119].

6. Genotyping and Characterizing the maize genetic variation map

6.1 SNP and INDEL calling and genotyping

The genotypes of SNPs and INDELs located on the AGPv4 reference genome were retrieved from [44]. Besides, we have also called the SNPs on the non-reference genome to estimate the pan-*Zea* gene dN/dS and Tajima’s D. We have mapped all the WGS reads to the pan-*Zea* genome using BWA mem with default options, and kept the reads that (at least one of the two reads in pair) mapped to the non-reference part of the genome for the downstream variant calling. We used sentieon version 201808.07 [120] to call the non-reference SNPs. Generally, the non-reference alignment bam files were inputted to sentieon to calculate the data quality summary information and remove the duplications using default options. The base quality score recalibration was then performed to remove experimental biases with default options. Then, the SNPs were called using the DNAscope algorithm with options “--emit_conf=10 --call_conf=10 --emit_mode gvcf”. Finally, the gvcf files of all the inbred lines were jointly called using GVCFtyper algorithm with default options. The resulting variant matrix was further filtered to keep records with MAF > 0.01 and genotype missing rate < 80% using bcftools as the final non-reference SNP set.

6.2 SV calling and genotyping

The SVs were called by combining the assembly-based methods and the WGS-reads-mapping based methods. The assembly-based SV calling evidences included SV calls from Assemblytics (hereafter referred to as AsmSV) and the non-reference insertions from the pan-*Zea* genome (hereafter referred to as NRINS). The WGS-reads-mapping based SV calling were performed with three classic NGS SV callers. Besides, a previously reported SV genotype matrix that included deletions and insertions called from the whole genome comparison between B73 and SK (hereafter referred to as BS-SV) was also included [20].

6.2.1 Assemblytics based SV (AsmSV) calling

The filtered scaftig to AGPv4 alignments (see section 2.2) were used as inputs for Assemblytics based SV calling. The Assemblytics script was modified to expand the maximum size of SV to 1Mb. Each inbred line’s SV calling result was converted to sequence aware VCF4.1 format, and SVs less than 50 bp were excluded. Then the SV result of all inbred lines were merged if they were the same SV type and their breakpoints were within 200 bp using SURVIVOR version 1.0.6 [121], and kept the merged record if the alternative allele was supported by at least one reference-levels-genome-to-AGPv4 evidence or at least three WGS-genome-to-AGPv4 evidences.

6.2.2 Non-reference insertions (NRINS)

The NRINSs were parsed from the non-reference sequences generated from pan-*Zea* genome construction and anchoring procedure (see section 2.3). Only the non-reference sequences that with both end anchored to the same chromosome within 1Mb region were consider as NRINSs. The NRINSs of all inbred lines were then merged and filtered with the same method as that of AsmSV.

6.2.3 WGS reads mapping based SV calling

The WGS read pairs for each inbred line were mapped to AGPv4 using BWA mem with default options, and the duplications were marked using sambamba version 0.5.9 [122]. Three SV callers, delly version 0.8.1 [123], gridss version 2.0.0 [124] and manta version 1.4.0 [125] were used.

For the delly pipeline, each inbred line's SVs were called with options of "-q 20 -r 20 -s 9" and excluded the gap regions greater than 100 bp and the centromere regions of AGPv4. Then the results were merged using delly merge with options of "-c -p -b 1000 -r 0.8 -n 1000000" to keep records less than 1 Mb and merge records with breakpoint offset less than 1 Kb and the reciprocal overlap rate greater than 80%. An additional delly call step with default parameters was performed using the merged SVs as reference to genotype the inbred lines. The genotyped vcf files were merged using bcftools and then filtered using delly filter with options of "-a 0.2 -r 0.75 -q 15 -e 0.8 -u 1.2" to get the final delly calls.

For the gridss pipeline, the SV calling was performed using the gridss' official pipeline with default options and the same excluded regions as the delly pipeline. The resulted SV calls were formatted by adding reference and alternative allele sequences and converting to vcf format. Each inbred line's calls were merged using SURVIVOR if they were the same SV type and their breakpoints were within 500 bp, and kept the records with a minimum minor allele number of three.

For the manta pipeline, each line's SV calls were identified using the default manta parameters and the same excluded regions as the aforementioned two WGS SV pipelines. The calls were formatted to add sequences to the sequence-aware records with custom script "04_fmt_manta_with_seq.sh", then filtered and merged using SURVIVOR with the same criteria as in the gridss pipeline.

6.2.4 Merging of all evidence, genotyping and filtering

The SV records of the five callers (AsmSV, NRINS, delly-SV, gridss-SV and manta-SV) were finally merged using SURVIVOR within 200 bp breakpoint offset. And the representation of each merged record was chosen based on the priority order of BS-SV, AsmSV (reference level assemblies), AsmSV (WGS assemblies), NRINS, gridss, manta then delly.

To get the genotype matrix of all merged SVs, we have performed two additional genotyping step, based on two SV genotyping tools, BayesTyper [126] and SVTyper [127]. For the BayesTyper-based genotyping procedure, only the sequence-aware SVs were selected because bayestyper use evidences from exact alignment of read k-mers to a graph representation of variants. Generally, the k-mers for each inbred line were calculated using K-Mer Counter (KMC) version 3.1.0 [128] with options of "-k55 -ci1". A read k-mer bloom filter for each sample were built using the 'makeBloom' module in BayesTyper version 1.5 with default parameters. To accurately estimate the noise parameters, we random selected one-tenth of the total SNPs and INDELs from the whole genome using custom script "get_rand_snpindel_SVs.sh" with a random seed of 12345, and all the unplaced contigs and organelle contigs in the reference genome were treated as decoy. Then we clustered the SNPs, INDELs and SVs using the 'cluster' module of BayesTyper. Each inbred line was genotyped using 'genotype' module in BayesTyper with default parameters. The genotyping outputs were then merged using bcftools with the parameters '--filter-logic x --info-rules ACP:max'. For the SVTyper-based genotyping procedure, we ran SVTyper version 0.7.0 using default options and taking the duplication masked bam file for each inbred line (see section 6.2.3) as input. The genotyping results were then merged using bcftools with default options.

So far, we have genotyping evidences of the SVs from bayestyper, delly, SVTyper and manta (hereafter referred to as BDMS-evidences), as well as evidences that could not distinguish between heterozygous and homozygous (AsmSV, gridss and NRINS, hereafter referred to as AGN-evidences). To get the final genotype matrix of the SVs, the BDMS-evidences and AGN-evidences were merged with custom script "PANZ_SV_Geno_merge.sh". Generally, for each SV in each inbred line, the BDMS-evidences were merged into BDMS-genotype by taking the most frequently occurred genotype in BDMS-evidences with weights of 2,1,1 and 1 for bayestyper, delly, SVTyper and manta, respectively. The AGN-evidences were also merged into AGN-genotype if at least one evidence support the alternative allele. The final genotype was assigned to (a) the BDMS-genotype if AGN-genotype is not available or consistent with BDMS-genotype, or (b) not available if BDMS-genotype is not consistent with AGN-genotype. The merged genotype matrix was then filtered to exclude SVs that with minor allele frequency greater than 0.01 and genotype missing rate greater than 0.8 using bcftools to get the final SV genotype matrix.

6.2.5 Estimating the representation

To compare the representation of our SV sets to the SVs generated in the NAM founders [39], we have i) estimated the proportion of the structural variants in the NAM project (hereafter referred to as "NAM-SV") detected based on both our unfiltered raw SV set (hereafter referred to as "raw-SV") and the final SV genotype matrix (hereafter referred to as

“final-SV”); and ii) estimated the genotyping compatibility between “final-SV” and NAM-SV based on the two germplasms (CML69 and CML228) shared between the two populations. The results were available on Github with url: https://github.com/songtaogui/panz-paper-data/tree/master/03_sv_pve_and_sv_snp_ld. The result showed that, our “raw-SV” set can cover ~84.13% (80971/96245) of the NAM-SVs (using the same SV merging criteria with those in [39], that is, taking SVs of the same type within 1000 bp as overlapped SVs). And after filtering, the final-SV set can cover ~42.89% (41283/96245) of the NAM-SVs. In general, the final-SV set showed a mean genotyping consistency of 92.68% (92.20% for CML69 and 93.16% for CML228) with the NAM-SVs.

6.3 Variant annotation

6.3.1 SNP and INDEL annotation

The SNPs and INDELs were annotated using the Ensembl variant effect predictor (VEP) [129] according to the pan-*Zea* annotation (a combination of reference annotation version 4.43 and the non-reference genome annotation in the current study) with options of “ --distance 2000 --pick”. The impact of genetic variants to nearby genes were assigned based on the annotations according to the criteria of Ensembl’s variant consequences.

6.3.2 SV annotation

The genetic or intergenic SVs were annotated based on the physical positions of the SV with the nearby genes using the custom script “PANZ_SV_Annotation.sh” by checking if the SV located within 2 Kb flanking regions of nearby genes. When assigning SVs with transposable elements (TEs), we annotated the SVs according to the previously released TE annotations of AGPv4 [130]. For non-insertion SVs, we assigned the TE annotation to the SV if more than 50% of the SV region is overlapped with the TE annotation and took the TE with highest overlapping rate as the representative annotation. For insertions, we annotated the insertion sequences using RepeatMasker with options “-cutoff 250 -nolow -no_is -norna” and took the TE with the highest score as the representative annotation. SVs with less than 50% region overlapped with TEs or with RepeatMasker mapping score less than 250 were not considered in the downstream TE related analyses.

6.4 SNP LD ranking analyses of InDels and SVs

To analysis the representation level by nearly SNPs of each SV and InDel, we have performed the LD ranking analyses according to the method in previous studies [20, 131]. Generally, for each common SV (MAF>5%), the nearest 150 upstream and 150 downstream common SNPs (MAF>0.05%) were selected. Pairwise genotype LD (r^2 values) were calculated within the 301 variants. The r^2 values were then ordered by decreasing rank and a median SNP-SNP rank value was calculated. For each of the 300 ranked surrounding positions, the number of times the SV rank was greater than the SNP-SNP median rank was calculated as a relative LD metric of SV to SNP. SVs with less than 100 ranks over the SNP-SNP median were classified as low-LD level. SVs with ranks between 100 and 200 were classified as mid-LD level, while SVs with greater than 200 ranks above their respective SNP-SNP median value were classified as high LD level with flanking SNPs. The pipeline was performed with custom scripts PANZ_SVflankSNP_LD.sh and SV_LD_type_draw.r.

7. Variant graph constructing, reads simulation and mapping

The genome graph representation of the genetic variants was constructed using vg version 1.30.0 [132]. Considering the limitations of the supported SV types in vg, only the SNPs, short INDELs and PAV type of SVs (insertions and deletions) were considered for the variant graph construction. The variant graph was constructed with options “construct -S -a -f -m 1000” for each AGPv4 chromosome and the resulting vg files were converted into GFA format with options “vg view –gfa” for downstream reads mapping.

To estimating the representation of the variant graph. We have compared all the NAM genomes to the AGPv4 reference genome using the same method in the pan-*Zea* genome construction section (See section 2.2). We random selected a 30 Mb region in AGPv4 (chr6:1-30000000) and get the sequences in the corresponding region of each NAM founder genome according to the whole genome comparison results. Then we simulated 100,000 paired-end 150 bp reads for each NAM founder using the randomreads.sh module of BBtools with options “reads=100000 length=150 paired=true maxq=35 midq=20 minq=5”. The simulated reads were then aligned to the variant graphs using GraphAligner version 1.0.11 [133] with default options.

8. Phenotype data collection and normalization

All the phenotypic data used in the study, including the raw complex traits including agronomic traits, grain moisture related traits, flowering time related traits and disease related traits, the traits of the primary and secondary metabolites, the transcript expression matrices of AMP kernels and leaves, the 2,750 proteins from the leaves of 98 AMP inbred lines and the differentially methylated regions (DMRs) of the AMP inbred lines, were retrieved from previous publications [32-36, 38, 134-139] and Maizego Resources (http://www.maizego.org/Resources.html), see Additional file 6: Table S5 for the categories and summaries , and the list of the individuals included in GWAS for each category was available at https://github.com/songtaogui/panz-paper-data/blob/master/05_samples_in_gwas/00_samples.tsv. The phenotypes were manually grouped based on their omics and features, while the metabolites were grouped according to PubChem classifications [140]. Each trait matrix was normalized for downstream analyses using rank-based inverse normal transformation embedded in the custom script “PANZ_rankINT.sh” with default options.

9. Estimating of narrow sense heritability

All the common reference genome based genetic variants (MAF > 0.05, located on the AGPv4 genome) were used for the narrow sense heritability (hereafter referred to as h^2) estimation. The genetic variants were imputed using beagle 4.0 (version r1399) [141] with options “window=50000 overlap=5000”. In order to estimating the possible h2 differences among different genetic variant features (positions relative to nearby genes, impact to nearby genes, MAF, variation types and sub-types, nearby SNP LD ranking levels and related TE types) in an unbiased manner, we need to resample or bin the genetic variants in each feature class to make sure the variants with different feature items are in the same volume when calculating h^2. For continuous features (MAF and nearby SNP LD ranking levels), the feature values were binned into five groups of equal frequency using discretize function of R/infotheo package version 1.2.0 [142]. For discrete features (positions relative to nearby genes, impact to nearby genes, variation types and sub-types and related TE types), we randomly resampled 100 times of each feature items using a sample size of 80% of the size of the minimum feature item. The resampling procedure was performed using custom script “PANZ_Rand_Variant_feature.sh” (with random seeds ranged from “001” to “100”).

The h^2 values of each phenotype according to the entire genetic variant sets and each subset that partitioned according to the features of the genetic variants were calculated using ldak version 5.0 [143]. Generally, for each inputted genetic variant set, the kinship matrix was calculated using ldak with options “--calc-kins-direct --ignore-weights YES --power -0.25”. Then the h^2 value was calculated using a generalized REML (restricted maximum likelihood) solver in ldak with options “--reml --constrain YES”. The h^2 values were then filtered to keep only the records with likelihood ratio test P value smaller than 0.05.

10. Identifying trait associated QTLs, genes and variants

10.1 GWAS and QTL analyses

The GWAS was performed for each normalized trait using a compressed mixed linear model accounting for the population structure and familial relationship [144] based on subsets of the 507 maize association mapping panel individuals (Additional file 6: Table S5). An easing P value cut-off that allowed 1 possible false discovery per independent association analysis was firstly used to get the “primary QTL” regions. The number of independent genetic variants (N_{ind}) was estimated by prune away variants with pairwise LD values (R^2) > 0.2 within 1Mb sliding window size, resulting in an easing P value cut-off of ~4.04e-06 (-log10(P value) of ~5.39). The primary QTL regions of each trait’s GWAS result were identified using Manhattan Harvester version 0.1 [145] with options “-inlimit 0.001 -peak-limit 5.39 -dots 5 -shrink 2”. The resulted QTL regions were further merged if their boundaries were within 20 Kb. For expression QTLs and methylation QTLs, instead of estimating the associations of all the genetic variants (which is time consuming), we have only estimated the *cis* expression QTLs (within flanking 1 Mb of the gene region) and the local methylation QTLs (within flanking 10 Mb of the differentially methylated region) [38]. The QTLs of the same trait that differed on years, locations, tissues (for expression) and experimental replications were clustered and merged to get the consensus QTL region according to their physical positions. If at least one of the QTLs in the QTL cluster has a leading -log10(P value) greater than the Bonferroni cut-off (~7.11), the QTL cluster was kept as the final QTL.

10.2 Fine-mapping of causal variants

We used DAP-G [146] to predict causal variant credible set for each QTL region with Bayesian multi-variant analysis. Generally, for each QTL region, the GWAS summary information of the genetic variants with P < 0.001 were retrieved from the GWAS results, and the LD matrix of these variants were calculated using plink version 1.90b4 [147] with default options. The fine-mapping step was performed using DAP-G with options of "-ld_control 0.25", and the credible sets of causal variants were constructed using the "get_credible_set.pl" utility of DAP-G (https://github.com/xqwen/dap/blob/master/utility/get_credible_set.pl) with a confidence posterior inclusion probability (PIP) cutoff of 0.95. The QTL identification and fine-mapping steps were embedded in the custom script "PANZ_QTL_FineMap.sh".

10.3 Identifying QTL type

The QTL type was defined as the genetic variant type of the variant with the highest PIP that within the 95% CI of that QTL. If two or more variants shared the same highest PIP, the longest variant was used. The INDEL/SV specific QTLs were defined as the QTLs that with the leading SNP P-values failed the Bonferroni cut-off.

11. Analyses of gPAV and SV cases

11.1 PAV_PZ00001a032490 and PME genes

The gPAVs that associated with phenotypes were defined as gPAVs that (a) located in the QTLs as identified in section section 10.1, (b) with association P values < 0.0001 and (c) with their genes expressed in no less than five individuals. The trait variations of ratio of seed sets in AMP was kindly provided by W. Li and Y.B. Wang, please refer to their research for more details [148]. The anchoring information of the PZ00001a032490 containing non-reference sequence PanRep_01830195 was retrieved from the pangenome anchoring procedure as described in section 2.3. The detailed multiple sequence alignments among the flanking sequences of the PME genes and their protein sequences were performed using MUSCLE v3.7 [149] with default parameters. The distribution of PME gene PAV pattern was drawn using iTOL v6.4 [150] by using the same tree structure as described in [44]. The significance P value of the association between numbers of accumulated PME genes and the ratio of seed sets was tested using Wilcoxon-Mann-Whitney test.

11.2 PZ00001aSV02097079INS and the expression of Zm00001d023299

The drought resistance trait data of AMP was retrieved from [59]. The re-sequencing reads and the expression patterns of the CUBIC parents, the haplotype data of CUBIC offsprings and their populational gene expression in V9 leaves were retrieved from [60], and could also be acquired online from MaizeCUBIC [151]. The epigenetic and TF binding information of maize leaves were retrieved from C3C4 ENCODE data portal (http://www.epigenome.cuhk.edu.hk) with a query region of "10:2344288-2356500". The *cis*-elements were predicted using PlantCARE [152] and only the predicted *cis*-elements that in the same strand with the target gene Zm00001d023299 were kept.

The activity of the predicted ABRE that breaked by PZ00001aSV02097079INS was estimated by luciferase (LUC) reporter assays using method as described in [153]. Generally, the promoter fragments of the target gene Zm00001d023299 with or without the predicted ABRE were amplified by PCR from the B73 line using primers listed in Additional file 8: Table S7, and were cloned into the multiple-cloning site of pGreenII 0800-LUC. A *Renilla* luciferase (REN) gene under the control of the 35S promoter in the same construct was used as an internal transformation control. The promoter-luciferase gene funcion constructs were then transient transformed in maize Mesophyll protoplasts isolated from leaves of 10-day-old etiolated B73 seedlings. The firefly LUC and REN activities were measured using the Dual-Luciferase Reporter Assay System (Promega, Madison, WI, USA), and the LUC activity of each construct was measured with three technical replicates for each of three biological replicates. Relative LUC activity was calculated by normalizing the firefly LUC activity to REN activity.

12. Miscellaneous statistical analyses and visualizations

12.1 Enrichment analyses

The gene ontology enrichment analyses were performed using GOATOOLS version 0.9.9 [154] with the GO terms and the GO slim terms updated on July 4th, 2019. The KEGG Enrichment Analyses were performed with the custom

script "PANZ_KEGG_enrich.sh" using the KEGG hierarchical text of plants (downloaded on January 6th, 2020) as references. The custom enrichment analyses of different gene, variant or QTL features were calculated using two-sided Fisher's exact test, with FPR corrected P values < 0.05 as significant cutoff. Specifically, for enrichment of gene features, the feature frequencies of associated genes (see section 10.2) were taken as queries and those of all the genes as targets; for enrichment of genetic variants, the feature frequencies of causal variants were taken as queries and those of associated variants (located within the QTL and had P-values < 0.001) as targets. The custom enrichment analyses were embedded in the script "PANZ_freq_enrich.sh". The pan-gene GO and KO annotation files were available at https://github.com/songtaogui/panz-paper-data/tree/master/06_ko_go_anno for the reproductions of the related enrichment results.

12.2 Permutation analyses

The permutations of the differences between the distribution of different gene features among core and dispensable genes were performed using the Wilcoxon-Mann-Whitney test embedded in the R/coin package [155] with 10,000 permutations.

12.3 Visualizations

The circos plot of the pan-*Zea* genome was drawn using R/circlize package [156]. The variant graph plot was retrieved from the graph genome Visualization tool Sequence Tube Map (https://github.com/vgteam/sequenceTubeMap). The basic statistic plots such as the histograms, boxplots, densities, lines and dots were drawn using R/ggplot2 [157]. The heat map plots were drawn using R/pheatmap [158]. The sankey plots were drawn using SankeyMATIC (http://sankeymatic.com/). The plots were composed using R/cowplot [159], R/patchwork (https://github.com/thomasp85/patchwork), and Adobe Illustrator CS6 (https://adobe.com/products/illustrator).
