# Supplementary material for: A pan-Zea genome map for enhancing maize improvement
Source: Genome Biol. 2022 Aug 23;23:178. doi: 10.1186/s13059-022-02742-7 (PMC9396798; doi:10.1186/s13059-022-02742-7)
Supplement: Supplementary file 13 — Additional file 13. Review history. [file 13059_2022_2742_MOESM13_ESM.docx]

Review History

**First round of review**

**Reviewer 1**

**Were you able to assess all statistics in the manuscript, including the appropriateness of statistical tests used?**

Yes, and I have assessed the statistics in my report.

**Comments to author:**

In their study "A pan-Zea genome map enhancing maize improvement", the authors use public sequencing data for the agriculturally important Zea genus to construct a pan-genome. The authors use this pangenome to detect structural variants and lineage-specific dispensable genes. Additionally, the authors conduct a GWAS using SNPs and structural variants, providing two interesting examples of structural variants associated with previously characterised QTL (Ga1 locus and IDP103).

I believe the study could be of broad interest and provides a useful community resource, though despite greater sample size and inclusion of teosinte samples it may not present a significant advance over previous work such as the pangenome study of de novo assembled maize founder lines (Hufford et al. 2021; doi: 10.1126/science.abg5289).

The study has appropriate methods and is well documented and I particularly appreciate that key data was uploaded to FigShare and code was uploaded to github. As noted under my 'Minor comments' however, there are some cases where additional data would be useful to share to further support the results presented in figures. Finally, while most conclusions of this study are well supported by the presented evidence, in a small number of cases I believe the authors overreach with their conclusions. In my comments below I outline in more detail some major areas of potential improvement for the paper as well as several more minor issues.

Major Comments

1. Although the authors report finding 69.52% additional non-reference sequence (Line 93) that was not identified in the NAM non-reference sequence, Hufford et al. (2021) report 103,033 pan-genes, which is almost twice as many as the 58,944 genes reported by the authors (Line 99). The authors thus seem to be finding much more sequences but much fewer genes. This unexpected discrepancy suggests some considerable limitations in the pangenome approach taken by the authors, which do not seem adequately addressed in the manuscript. Moreover, as shown in Table S3, the non-reference genes presented in this study have a mean length of 995bp as compared to the mean length of 4162bp in the AGP v4 reference genes. This discrepancy is often found in short read based pangenome studies and further suggests technical limitations in assembling and/or detecting genes based on short reads. As the authors write on Line 461 their methods may lead to an "underestimate of the teosinte sequences and genes within the pan-Zea". Thus, a closer comparison with Hufford et al. (2021) and clearer assessment of the limitations of the short read approach would be useful.

2. In a similar vein, what proportion of structural variation do the authors expect to be able to access using their short read approach? Specifically, it may help to report what proportion of the Hufford et al. (2021) structural variants and pan-genes were detected by the authors.

3. As described in Section "3. Pan-genome gene annotation" in Additional File 4 (Supplementary Materials and Methods) "The final pan Zea gene models were the combination of the non-reference gene models and the AGPv4.43 reference maize gene models." This suggests that the full gene set is a mosaic of two distinct gene annotation approaches, potentially biasing downstream analyses. For this reason many pangenome studies carry out gene annotations for the entire pangenome from scratch. Although I think the authors' approach can be justified based on wanting to use the relatively high quality AGP v4 annotations, I think it would be helpful to explicitly justify this choice of approach based on the literature or data from the study.

4. In the sections describing the QTL results, the authors make some outlandish claims. For example on Line 270, the authors write that "the combination of all QTLs covered almost the entire genome (~99.35%). These findings suggest that each sequence of the genome may be functional (Figure 4B)". In the discussion of these results on Line 423 the authors further note that "It has been reported that ~85% of all common human genetic variation is at least nominally associated with gene expression [72]. Correspondingly, we found that ~84.58% of the maize genomic regions were covered with cis-eQTL, and more than 99% of the genomic regions were covered with at least one biochemical or phenotypical trait". The suggestion that all sequences of the highly repetitive maize genome may be functional is an extraordinary claim and the low resolution QTL intervals inferred in this study are insufficient to support it. Further the authors seem to be suggesting that 85% coverage of the maize genome with cis-eQTL corresponds to 85% of human genetic variation being associated with gene expression, despite this being an apples to oranges comparison (QTL intervals versus variants). I would suggest revising or adding more evidence for these extreme claims, and further explanation of why such high QTL coverage is not a result of low resolution mapping and the QTL merging described in the supplementary methods.

5. In the discussion (Lines 394-397) the authors suggest that "many of the SVs (~37.36%) were not well represented by nearby SNPs" and "SVs were more likely to be the cause of phenotype variation than SNPs and InDels". Earlier on Line 274 the authors also write that "The proportion of SV-QTLs was much higher than the proportion of SVs in all of the variants, indicating that SVs are more likely to lead to functional changes." These findings seem to me to be contrary to expectations. It would thus be worthwhile to contrast these findings with with Hufford et al. (2021), who found that SVs are in high linkage disequilibrium with SNPs and combining SNPs and SVs only marginally increases the percentage of variance explained for a range of traits (see section of their manuscript titled "Structural variation and impact on phenotype").

6. Overall, the paper has a high volume of information which can be challenging to take in. For example, the authors prepared 5 main figures, each with 8-11 panels. Similarly, the Results contain a large amount of information from disparate analyses. Although this is more of a take-it-or-leave-it comment that is not essential to address, I would suggest that the authors shift some information to the supplement to focus more on presenting the results they find the most interesting to readers.

7. The analysis on gene PAV (trends in gene loss and GO enrichment) shown in Figure 2 and described on lines 153 to 207 are based on analyses including custom analyses with Fisher's exact test, but the data including p-values does not seem to be included in the manuscript. I suggest the authors add tables with this data to the supplement to make it easier for readers to follow up on genes of interest that the authors found to show signs of GO enrichment or lineage-specific evolution.

Minor Comments

8. There are minor typos and wording issues, mostly in Results and Methods/Supplementary Methods. I would emphasize that this is a minor issue and the manuscript is overall very clear and well-written.

9. I found it a little unclear what samples were used for the GWAS based on the main text. Based on the supplementary methods I am assuming this was based on a subset of the AMP lines. However, this still seems a little unclear and I think the manuscript would benefit from a clarification of the GWAS sample size and a list of the included individuals.

10. Several of the URLs linking to data and code in the references have formatting issues (often missing a full stop). For example, the FigShare data sharing link is "https://doiorg/106084/m9figshare19097447v1".

11. L59: change „stable crop" to „staple crop"

12. In the Figure S3 legend, I would clarify what RP and SR stand for. One could refer to the Supplementary Methods and/or explain these indicate read pairs and split reads.

13. L228 I found this sentence difficult to understand: "The SNPs, InDels, and SVs showed similar MAF distribution patterns that those with more variations had smaller MAF values (Figure 3F)."

14. L293: Do you mean genotypes? "gPAVs was highly useful in identifying the phenotypes of candidate genes, which could not be directly detected using a single reference genome"

15. L297: It may be helpful to quantify the enrichment stated in the sentence "These PME genes all showed similar presence/absence patterns with PZ00001a032490, and the presence of these PME genes was enriched in teosintes (Figure I)."

**Reviewer 2**

**Were you able to assess all statistics in the manuscript, including the appropriateness of statistical tests used?**

Yes, and I have assessed the statistics in my report.

**Comments to author:**

In this manuscript, the authors presented a pangenome for maize and its wild relatives based on the published high quality NAM genomes (https://www.science.org/doi/10.1126/science.abg5289) and ~700 low quality assemblies generated by Illumina PE reads (~20X, ~200 newly sequenced). I agree with the authors that maize is a super important crop and a high quality pan-reference genome is required for maize genetics and breeding. However, there is an obvious imbalance of genome qualities of NAM assemblies and Illumina assemblies. In other words, the 4.6 Gb non-B73 sequences may be mainly from NAM samples. I also had some other major and minor comments for the authors.

Major:

1. The authors made great efforts to combine LOTS of analyses in this study, however, I didn't see much novel aspects. The authors argued that > 97% SVs are linked with SNPs. Is this true? Based on the population genetic analyses of SVs in drosophila (https://www.nature.com/articles/s41467-019-12884-1), grape (https://www.nature.com/articles/s41477-019-0507-8) and rice (https://doi.org/10.1093/molbev/msaa185), the SVs are mostly unlinked with SNPs, and under strong purifying selection with low population frequency. At later paragraphs, the authors also pointed that SVs explained ~70% while SNPs explained ~60% of the phenotypic variants. This has been a major point in this review paper: https://doi.org/10.1038/s41477-018-0210-1 that some of the missing heritability might be hidden in SVs. However, the genetic basis is that SVs are not linked with SNPs. I suggest the authors to check the filtering steps of SVs. To me, the SVs with low frequency might had been removed? or there was a genotyping problem for SVs with low population frequency.

2. The maize pangenome had already been generated by Hufford et al based on high quality NAM genomes. In my mind, the adding of ~700 low quality assemblies could NOT make big differences because the SVs (especially insertions) are mostly undetectable by Illumina reads or low quality assemblies. The authors could either adding several high quality assemblies of wild maize to make the pan-zea genome or make more efforts on the application of the NAM pangenome, and could focus on the gain and loss of genes and regulatory elements during maize domestication and improvement, as well as the population genetics and quantitative genetics of SVs in maize.

3. It is unclear to me whether the samples was collected from global sampling or restricted to some regions. The detailed information should be provided for the ~700 resequenced samples, for example, the GIS information. At the same time, the authors didn't offer any explanations why different number of samples were used in different analyses, which made the related parts very hard to follow.

4. Why only PAVs were analyzed in detail. Many adaptive and phenotypes were associated with INVersions in maize literature. It might be interesting to revisit the classical examples using the new SV genomic map. Such analyses had been conducted in tomato (https://www.sciencedirect.com/science/article/pii/S0092867420306164). At the same time, some of the analyses might be problematic, for example, what is an OPAV? Do the authors mean derived PAVs? Does it overlap with gPAV? Please use standard terms instead.

5. The figures in the pdf file is barely readable to me, please use high-resolution figures.

Minors:

L68: why only 11 were used? There were more than 25 high quality maize assemblies available.

L69: Please describe the design of the sampling.

L90-92: Please check the numbers here.

L119: "7% were dispensable", this is very low number, I suspect the the low frequency or sample specific gPAVs were under estimated in this study.

L120-122: These are big numbers, does this mean ~1/5 gene content difference between any given pair of maize samples.

L1132: What about variants with a length of 50?

L156-158: What does the authors mean by "the direction evolution"?

L178-207: I suggest the authors to use standard population genetic methods.

L216-218: There is an obvious insertion-deletion imbalance here. At the same time, INS and DEL should be relatively defined based on the derived status.

L225: This is much lower than in rice (https://doi.org/10.1093/molbev/msaa185). Did the authors annotate the INSertions properly?

L252: This is interesting and worth further digging.

L439-440: Only ~33%? This should be much more if high quality data is used.

**Authors Response**

**Point-by-point responses to the reviewers’ comments:**

Reviewer #1:

In their study "A pan-Zea genome map enhancing maize improvement", the authors use public sequencing data for the agriculturally important Zea genus to construct a pan-genome. The authors use this pangenome to detect structural variants and line-age-specific dispensable genes. Additionally, the authors conduct a GWAS using SNPs and structural variants, providing two interesting examples of structural variants associated with previously characterised QTL (Ga1 locus and IDP103).

I believe the study could be of broad interest and provides a useful community re-source, though despite greater sample size and inclusion of teosinte samples it may not present a significant advance over previous work such as the pangenome study of de novo assembled maize founder lines (Hufford et al. 2021; doi: 10.1126/science.abg5289).

The study has appropriate methods and is well documented and I particularly appreci-ate that key data was uploaded to FigShare and code was uploaded to github. As noted under my 'Minor comments' however, there are some cases where additional data would be useful to share to further support the results presented in figures. Fi-nally, while most conclusions of this study are well supported by the presented evi-dence, in a small number of cases I believe the authors overreach with their conclu-sions. In my comments below I outline in more detail some major areas of potential improvement for the paper as well as several more minor issues.

*Response: Thank you for the nice and constructive comments. We have performed detailed comparisons between the genes and SVs in the current study with those in NAM founder genomes, removed the overreach conclusions, and revised minor issues as suggested by the reviewer. Please find below the detailed responses one by one.*

Major Comments

1. Although the authors report finding 69.52% additional non-reference sequence (Line 93) that was not identified in the NAM non-reference sequence, Hufford et al. (2021) report 103,033 pan-genes, which is almost twice as many as the 58,944 genes reported by the authors (Line 99). The authors thus seem to be finding much more sequences but much fewer genes. This unexpected discrepancy suggests some consid-erable limitations in the pangenome approach taken by the authors, which do not seem adequately addressed in the manuscript. Moreover, as shown in Table S3, the non-reference genes presented in this study have a mean length of 995bp as compared to the mean length of 4162bp in the AGP v4 reference genes. This discrepancy is of-ten found in short read based pangenome studies and further suggests technical limi-tations in assembling and/or detecting genes based on short reads. As the authors write on Line 461 their methods may lead to an "underestimate of the teosinte se-quences and genes within the pan-Zea". Thus, a closer comparison with Hufford et al. (2021) and clearer assessment of the limitations of the short read approach would be useful.

*Response: Thank you for the suggestions. We agree with the reviewer that it is necessary to provide a closer comparison with the NAM pan-gene sets. However, several factors need to be considered before performing comparison of the pan-gene sets between our “pan-Zea” and NAM:*

*i). During the construction of the pan-Zea genome, the non-reference sequences were deduped based on the similarity and anchor-information. That is, the redundant removing steps were performed within each anchor-cluster, or within the unanchored sequences (as illustrated in Section 2.3 of Supplemental Materials and Methods). These criteria were implemented in order to keep as many as “non-reference insertions” (Sherman et al., 2019). However, this would also lead to an inflated non-reference sequence size;*

*ii). The criteria to construct the pan-gene sets differs between “mul-ti-reference-genomes-based project” and “population-level-WGS-based project”. When multiple high-quality reference genomes and consensus annotations are availa-ble, pan-gene sets were usually constructed based on inferring one-to-one homolog relationships based on both sequence similarity and synteny blocks, as the NAM-project did. However, in WGS-based projects, we cannot get enough synteny evidences to distinguish the true homologs from other sequence similarities (for exam-ple, paralogs) due to the fragmental assemblies. Besides, the present of high-similar genes would affect the reads-mapping-based gene PAV analyses. We think it is more import to accurately genotyping gene PAVs among populations than enlarging the pan-gene set sizes in our project. Thus, several filtering steps were added after the initial annotation of pan-gene sets (as illustrated in Section 3 of Supplemental Materi-als and Methods, and Figure S4) to exclude those shown high-similarity with the kept ones (similar procedure was also performed in Rice 3K project (Wang et al., 2018)).*

*With these factors in mind, in order to make a clearer assessment between pan-Zea and NAM pan-gene sets, we have compared the NAM pan-genes with the unfiltered raw pan-Zea genes with Blastp (hits with e-value < 1e-5 and at least 80% protein similarity and 80% either coverage were kept). The results revealed that ~81.08% (82,535/103,033) of the NAM pan-genes could be matched with the pan-Zea genes (please refer to ResponseFigure 1A with url: https://github.com/songtaogui/panz-paper-data/blob/master/99_figures/Response_Figure1.pdf). And ~6.97% of the NAM pan-gene hits were lost during the pan-Zea gene filtering procedure. This is a consequence we could bear, because the filtering proce-dure have also removed 96.36% low-quality gene annotations. The results are available on Github with url: https://github.com/songtaogui/panz-paper-data/tree/master/01_vs_nam_pan-genes*

*To further check if there are length biases in WGS based pan-gene annotation, we have compared the distributions of several genic features between the NAM pan-genes that cannot be matched with pan-Zea (referred to as “NAM unique” in Re-sponseFigure 1B) and the pan-Zea genes cannot be matched with NAM (referred to as “pan-Zea unique” in ResponseFigure 1B with url: https://github.com/songtaogui/panz-paper-data/blob/master/99_figures/Response_Figure1.pdf). The result showed that the pan-Zea unique genes have in general longer gene length, longer protein length, and more exons, than the NAM unique ones. However, the per exon length and intron length of the pan-Zea unique genes were shorter. These results indicate that, the gene length differences between the reference genes and the non-reference genes were largely due to the gene filtering step, and the WGS based pan-gene annotations may be limited in identifying genes with large exon or introns.*

*To sum up:*

*For the reviewer’s concern of “finding more sequences but much fewer genes than NAM”, we think the reasons would be a) the redundancy of the pan-Zea se-quences and the non-redundancy of the pan-Zea genes; b) the gene annotation filter-ing procedure and c) the limitation of the WGS-based pan-gene identification.*

*For the reviewer’s concern of the discrepancy between reference genes and non-reference genes, we think it is mainly caused by the removing of redundant, while the limitation of the short-read approach in identifying genes with longer exon/intron could also be possible factors.*

*For the reviewer’s concern of “the clearer assessment of the limitations of the short-read approach”, our analyses indicated that our approach has the potential of covering at least 81.08% of the NAM pan-gene sets, indicated the limitation of the short read approach would be the ~20% missing of the pan-genes. However, this is still a rough estimation, since we currently lack the reference genomes and reference gene annotations of other Zea species.*

*We have revised the Supplementary Text to add the above comparison between NAM and pan-Zea gene sets. We hope these would answer the reviewer’s concerns and provide clearer information for the readers.*

2. In a similar vein, what proportion of structural variation do the authors expect to be able to access using their short read approach? Specifically, it may help to report what proportion of the Hufford et al. (2021) structural variants and pan-genes were detect-ed by the authors.

*Response: Similar to the situation of pan-gene sets, the final structural variations in our study were heavily filtered by combining multiple evidences (as illustrated in Section 6.2.4 of Supplemental Materials and Methods) to guarantee the genotype accuracy and the reliability of the downstream analyses. Besides, the NAM SVs were from 26 maize germplasms while the dataset in our study contain 507 maize germplasms, there are chances that the SVs provided in the NAM founders didn’t show reasonable allele frequencies in the 507 maize panel and thus would not be included in the final result of our SV genotype matrix.*

*Thus, to compare the two SV sets in an unbiased way, we have a) estimated the proportion of the structural variants in the NAM project (hereafter referred to as “NAM-SV”) detected based on both our raw SV set (hereafter referred to as “raw-SV”) and the final SV genotype matrix (hereafter referred to as “final-SV”); and b) estimated the genotyping compatibility between our “final-SV” and NAM-SV based on the two germplasms (CML69 and CML228) shared between the two popu-lations. The results are available on Github with url: https://github.com/songtaogui/panz-paper-data/tree/master/03_sv_pve_and_sv_snp_ld*

*The result showed that, our “raw-SV” set can cover ~84.13% (80971/96245) of the NAM-SVs (using the same SV merging criteria with those in the Hufford et al. (2021), that is, taking SVs of the same type within 1000 bp as overlapped SVs). And after filtering, the final-SV set can cover ~42.89% (41283/96245) of the NAM-SVs. In general, the final-SV set showed a mean genotyping consistency of 92.68% (92.20% for CML69 and 93.16% for CML228) with the NAM-SVs.*

*These analyses indicated that the pan-Zea genome and the WGS data here has the potential of covering ~84.13% of the NAM SVs, and the final SV sets has a reasona-ble genotyping accuracy for downstream analyses. We have updated the Supplemen-tary Material and Methods in the revised manuscript to include the above estimations in a new section “6.2.5 Estimating the representation”.*

3. As described in Section "3. Pan-genome gene annotation" in Additional File 4 (Supplementary Materials and Methods) "The final pan Zea gene models were the combination of the non-reference gene models and the AGPv4.43 reference maize gene models." This suggests that the full gene set is a mosaic of two distinct gene an-notation approaches, potentially biasing downstream analyses. For this reason many pangenome studies carry out gene annotations for the entire pangenome from scratch. Although I think the authors' approach can be justified based on wanting to use the relatively high quality AGP v4 annotations, I think it would be helpful to explicitly justify this choice of approach based on the literature or data from the study.

*Response: The strategy was adopted from the pan-genome analyses of 3,010 diverse acces-sions of Asian cultivated rice (Wang et al., 2018), and similar strategies were applied in the construction of many pan-genomes (Golicz et al., 2016b; Hübner et al., 2019; Montenegro et al., 2017). The commonly used WGS-based pan-genome constructing strategies (Golicz et al., 2016a) all need to compare with the reference genome, either before assembly (iterative assembly strategy) or after assembly (de novo assembly strategy, as used in our study). Thus, the potential biases may have already been in-troduced during that procedure. To our limited knowledge, many (if not all) linear representation of pan-genomes (Hübner et al., 2019; Sherman et al., 2019) and even graphic pan-genomes (Li et al., 2020; Liu et al., 2020) leveraged the reference genome as framework, and the implementation of unbiased graphic pan-genome is still in pro-gress (https://github.com/pangenome/pggb). Besides, as illustrated in response to the reviewer’s first concern, to ensure the accuracy of gene PAV calls using the map-to-pan strategy, a high-quality reference gene annotation is needed.*

*The using of a maize genome as reference may most likely bias the analyses of teosinte specific genes. An estimation based on an unpublished teosinte genome (please refer to the last paragraph of the answer to Reviewer 2’s 2nd major concern) indicated that the PAV of teosinte specific genes showed a similar accuracy with those in maize, which indicated that the downstream analyses may not be appreciably biased.*

4. In the sections describing the QTL results, the authors make some outlandish claims. For example on Line 270, the authors write that "the combination of all QTLs covered almost the entire genome (~99.35%). These findings suggest that each sequence of the genome may be functional (Figure 4B)". In the discussion of these results on Line 423 the authors further note that "It has been reported that ~85% of all common human genetic variation is at least nominally associated with gene expression [72]. Corre-spondingly, we found that ~84.58% of the maize genomic regions were covered with cis-eQTL, and more than 99% of the genomic regions were covered with at least one biochemical or phenotypical trait". The suggestion that all sequences of the highly re-petitive maize genome may be functional is an extraordinary claim and the low resolu-tion QTL intervals inferred in this study are insufficient to support it. Further the au-thors seem to be suggesting that 85% coverage of the maize genome with cis-eQTL corresponds to 85% of human genetic variation being associated with gene expression, despite this being an apples to oranges comparison (QTL intervals versus variants). I would suggest revising or adding more evidence for these extreme claims, and further explanation of why such high QTL coverage is not a result of low resolution mapping and the QTL merging described in the supplementary methods.

*Response: Thank you for pointing out this arbitrary claim. We now realized that it is inap-propriate to compare the QTLs from a small GWAS population as in the current study to those from a much larger human population. Considering that the original idea of these claims was to describe the general distribution of the multi-omics QTLs, and the present of these claims didn’t support the main findings in the study, we thus decided to remove the related descriptions from the revised manuscript, to avoid inadvertently misleading the reader.*

5. In the discussion (Lines 394-397) the authors suggest that "many of the SVs (~37.36%) were not well represented by nearby SNPs" and "SVs were more likely to be the cause of phenotype variation than SNPs and InDels". Earlier on Line 274 the authors also write that "The proportion of SV-QTLs was much higher than the propor-tion of SVs in all of the variants, indicating that SVs are more likely to lead to func-tional changes." These findings seem to me to be contrary to expectations. It would thus be worthwhile to contrast these findings with Hufford et al. (2021), who found that SVs are in high linkage disequilibrium with SNPs and combining SNPs and SVs only marginally increases the percentage of variance explained for a range of traits (see section of their manuscript titled "Structural variation and impact on phenotype").

*Response: In the aspect of the reviewer’s concern on the percentage of variance explained by SV, Hufford et al. (2021) drawn the conclusion that SVs only marginally increases the percentage of variance explained (hereafter referred to as PVE) by comparing the PVE of the combination of SVs and SNPs (hereafter referred to as Total-PVE) to the PVE of SNPs only (hereafter referred to as SNP-PVE), based on 36 complex traits. Similarly, in our study, we found that the PVE of only SVs (hereafter referred to as SV-PVE) were slightly smaller than SNP-PVE in complex agronomic traits (as illus-trated in Figure 3I of the original submission, now as Figure 3C of the revised manu-script). To make a thorough comparison with the results in (Hufford et al., 2021), we have also calculated the Total-PVEs based on the multi-omics traits in our study, and compared them with the SNP-PVEs. The results (as illustrated in ResponseFigure 2A with url: https://github.com/songtaogui/panz-paper-data/blob/master/99_figures/Response_Figure2.pdf) also support the conclusion of adding SV only marginally increased the PVE. We think these results were largely influenced by the differences in scales between SNPs and SVs, which was also highlighted in Hufford et al. (2021) that “Much of the phenotypic variation was also explained by SNPs, which were much more numerous (288-foldmore) relative to our conservative set of SVs”. In fact, that’s the main moti-vation for us to performed the permutation-based PVE analyses (as illustrated in Fig-ure 3K of the original submission) to compare SNP-PVE and SV-PVE in the same scale.*

*About the reviewer’s concern on the LDs between SNPs and SVs, SVs were found not highly linked with SNPs in many species, e.g., in Arabidopsis (Stuart et al., 2016), grape (Zhou et al., 2019) and rice (Kou et al., 2020), as also mentioned by Re-viewer 2. Besides, we failed to find data or references in the main article and the sup-plementary information in (Hufford et al., 2021) to support their conclusion of “most SVs are in high linkage disequilibrium with SNPs”. Since we cannot figure out their criteria for estimating the LD between SVs and SNPs, we have decided to test the LD levels between NAM SVs and NAM SNPs with our criteria (as illustrated in Sec-tion 6.4 of Supplemental Materials and Methods, with codes available as https://github.com/songtaogui/pan-Zea_utilities/blob/master/PANZ_SVflankSNP_LD.sh) using the SVs and SNPs genotyping data in 4,950 NAM RILs (the data was ac-quired from Cyverse, with path of /iplant/home/shared/NAM/NAM_genome_and_annotation_Jan2021_ re-lease/SUPPLEMENTAL_DATA/NAM-SV-projected-V9/NAM_rils_projected-SNPs-SVs.projected.final.v9.hmp.txt.gz, considering that the whole dataset is huge and estimating LD levels between all SVs and SNPs is time-consuming and hard for the reviewer to reproducing, we have only analyzed the SV and SNPs on chromosome 1 of the maize genome, which including 15,595 SVs). The results, as illustrated in Re-sponseFigure 2B (available with url: https://github.com/songtaogui/panz-paper-data/blob/master/99_figures/Response_Figure2.pdf), showed a similar pattern with those in our study, that ~36.87% of the NAM SVs cannot be well represented by nearby SNPs (compared with ~37.36% in our study).*

*To sum up, the accumulated PVE of all variations showed similar results with those in (Hufford et al., 2021), and the LD levels between SNPs and SVs from (Hufford et al., 2021) also showed a similar pattern with those in our study when ap-plying the same criteria. The above results are available on Github with url: https://github.com/songtaogui/panz-paper-data/tree/master/03_sv_pve_and_sv_snp_ld*

6. Overall, the paper has a high volume of information which can be challenging to take in. For example, the authors prepared 5 main figures, each with 8-11 panels. Sim-ilarly, the Results contain a large amount of information from disparate analyses. Alt-hough this is more of a take-it-or-leave-it comment that is not essential to address, I would suggest that the authors shift some information to the supplement to focus more on presenting the results they find the most interesting to readers.

*Response: We appreciate the reviewer’s nice comments and the suggestions for making the manuscript more readable. In order to highlight the main findings of the study, we have now moved some sub-panels of Figure 3 to Supplementary Figure S13, to focus more on the main features of SVs in the main manuscript.*

7. The analysis on gene PAV (trends in gene loss and GO enrichment) shown in Fig-ure 2 and described on lines 153 to 207 are based on analyses including custom anal-yses with Fisher's exact test, but the data including p-values does not seem to be in-cluded in the manuscript. I suggest the authors add tables with this data to the sup-plement to make it easier for readers to follow up on genes of interest that the authors found to show signs of GO enrichment or lineage-specific evolution.

*Response: Thank you for the suggestion. We have provided the GO and KO enrichment re-sults in two additional Supplementary Tables, namely Table S5 and Table S6 in the revised manuscript, and the raw Table S5-7 were renamed as Table S7-9 accordingly. In consideration of the reproducibility, we have also made the raw GO and KO anno-tations of the pan-Zea genes accessible at https://github.com/songtaogui/panz-paper-data/tree/master/06_ko_go_anno and up-dated Section 12.1 of the Supplementary Materials and Methods in the revised manu-script to include the accession accordingly.*

Minor Comments

8. There are minor typos and wording issues, mostly in Results and Meth-ods/Supplementary Methods. I would emphasize that this is a minor issue and the manuscript is overall very clear and well-written.

*Response: We apologize for the careless typos. Thank you for kindly pointing them out. We have reviewed the manuscript and changed the typos listed below:*

*1) “Figure I” on Line 291 and Line 298 should be “Figure 4I”, and now as “Figure 4H” in the revised manuscript;*

*2) Tissue label “tassel” should be “ear” in Figure 5E;*

*3) “Transparent Method” should be “Supplementary Materials and Methods” in Ad-ditional files;*

*4) “The NGS reads of the teosinte landrace and the maize AMP individuals ...” is revised to “The NGS reads of the teosintes, landraces and the maize AMP indi-viduals” in Supplementary Test;*

*And the mistakes that listed by the reviewers were all corrected in the revised manu-script.*

9. I found it a little unclear what samples were used for the GWAS based on the main text. Based on the supplementary methods I am assuming this was based on a subset of the AMP lines. However, this still seems a little unclear and I think the manuscript would benefit from a clarification of the GWAS sample size and a list of the included individuals.

*Response: Yes, all the GWAS were performed based on subsets of the 507 AMP lines. To make the sample size more explicit, we have updated the Table S5 in Supplementary Tables to add additional columns for recording the sample sizes of each trait group. Besides, we have uploaded a file that listed all the sample names for each trait group to Github (https://github.com/songtaogui/panz-paper-data/blob/master/05_samples_in_gwas/00_samples.tsv), and updated the Supplementary Materials an Methods to add the acces-sion in the revised manuscript accordingly.*

10. Several of the URLs linking to data and code in the references have formatting issues (often missing a full stop). For example, the FigShare data sharing link is "https://doiorg/106084/m9figshare19097447v1".

*Response: We apologized for that mistake. The URLs have now been updated.*

11. L59: change „stable crop" to „staple crop"

*Response: We have corrected the description accordingly.*

12. In the Figure S3 legend, I would clarify what RP and SR stand for. One could refer to the Supplementary Methods and/or explain these indicate read pairs and split reads.

*Response: We have added a description of “RP and SR, for read pairs and split reads, re-spectively” in the Figure S3 legend.*

13. L228 I found this sentence difficult to understand: "The SNPs, InDels, and SVs showed similar MAF distribution patterns that those with more variations had smaller MAF values (Figure 3F)."

*Response: We apologized for the unclear description. What we mean is the three type of variations all showed a similar MAF distribution patterns that most of the variation were rare in the population (with small MAF values). We have changed the descrip-tion to “... showed similar MAF distribution patterns that skewed toward rare vari-ants”*

14. L293: Do you mean genotypes? "gPAVs was highly useful in identifying the phenotypes of candidate genes, which could not be directly detected using a single reference genome"

*Our response: We have revised this description as “gPAVs was highly useful in identifying can-didate genes, which ...”*

15. L297: It may be helpful to quantify the enrichment stated in the sentence "These PME genes all showed similar presence/absence patterns with PZ00001a032490, and the presence of these PME genes was enriched in teosintes (Figure I)."

*Response: We have added statistical tests and revised the sentence as “... and the presence of these PME genes was enriched in teosintes (with presence ratio of ~85.97% in teo-sintes and ~40.35% in maize, Fisher’s exact test P-value < 2.2e-16)...”.*

Reviewer #2:

In this manuscript, the authors presented a pangenome for maize and its wild relatives based on the published high quality NAM genomes (https://www.science.org/doi/10.1126/science.abg5289) and ~700 low quality assem-blies generated by Illumina PE reads (~20X, ~200 newly sequenced). I agree with the authors that maize is a super important crop and a high quality pan-reference genome is required for maize genetics and breeding. However, there is an obvious imbalance of genome qualities of NAM assemblies and Illumina assemblies. In other words, the 4.6 Gb non-B73 sequences may be mainly from NAM samples. I also had some other major and minor comments for the authors.

*Response: Thank you for the comments. We apologize if we didn’t make the manuscript as explicit as we expected to. Instead of leveraging the NAM genomes when construct-ing the pan-Zea genome, we used them as an assessment to the representation of the pan-Zea genome for the maize genetic repertoire. Our analyses, as illustrated in the result section of the manuscript, indicated that the 4.6 Gb non-B73 sequences were largely based on non-NAM-origin sequences (as estimated in the revised manuscript, ~76.15% of the pan-Zea non-reference sequences could not be represented by the NAM non-reference sequences). Based on the results and the additional analyses as suggested by the Reviewer 1, we believe that our pan-Zea genome have both shown reasonable representativeness of the maize genetic repertoire and provided considera-ble new genetic resources for the community. Please find the detailed responses one by one in the following.*

Major Comments

1. The authors made great efforts to combine LOTS of analyses in this study, however, I didn't see much novel aspects. The authors argued that > 97% SVs are linked with SNPs. Is this true? Based on the population genetic analyses of SVs in drosophila (https://www.nature.com/articles/s41467-019-12884-1), grape (https://www.nature.com/articles/s41477-019-0507-8) and rice (https://doi.org/10.1093/molbev/msaa185), the SVs are mostly unlinked with SNPs, and under strong purifying selection with low population frequency. At later para-graphs, the authors also pointed that SVs explained ~70% while SNPs explained ~60% of the phenotypic variants. This has been a major point in this review paper: https://doi.org/10.1038/s41477-018-0210-1 that some of the missing heritability might be hidden in SVs. However, the genetic basis is that SVs are not linked with SNPs. I suggest the authors to check the filtering steps of SVs. To me, the SVs with low fre-quency might had been removed? or there

was a genotyping problem for SVs with low population frequency.

*Response: For the reviewer’s concern of “> 97% SVs are linked with SNPs”, we think it may be referred to Line 109-112 of the original submission “the gPAVs were related to the population structure and were well represented by SNPs with ~97.37% gPAVs displaying high LD with nearby SNPs”. This result was the LD levels of nearby SNPs for gene PAVs, not for SVs (which is illustrated in the 3rd sub-section of the Result section).*

*We agree with the reviewer that SVs are not highly linked with SNPs. In fact, in the current study, we estimated a proportion of ~37.36% for SVs that cannot be well represented by nearby SNPs (as illustrated in Figure 3G and line 230-233 of the initial submission). A similar proportion was shown in the SVs of the NAM population (please refer to ResponseFigure 2B and related descriptions in the answers to Re-viewer 1’s 5th major concern). These findings are in close agreement with the findings in the listed researches by the reviewer.*

*Regarding the reviewer’s concern of SV filtering and genotyping. We filtered the SVs as illustrated in Section 6.2.4 of Supplementary Materials and Methods. Besides, we have performed a comparison with the NAM SVs, as illustrated in response to Reviewer 1’s 2nd major concern. The results showed a general SV genotyping accu-racy of 92.68%, which to us is acceptable for downstream analyses.*

2. The maize pangenome had already been generated by Hufford et al based on high quality NAM genomes. In my mind, the adding of ~700 low quality assemblies could NOT make big differences because the SVs (especially insertions) are mostly unde-tectable by Illumina reads or low quality assemblies. The authors could either adding several high quality assemblies of wild maize to make the pan-Zea genome or make more efforts on the application of the NAM pangenome, and could focus on the gain and loss of genes and regulatory elements during maize domestication and improve-ment, as well as the population genetics and quantitative genetics of SVs in maize.

*Response: Thank you for the suggestions. We agree with the reviewer that the high-quality NAM founder genomes are valuable pan-maize genomic resources, and could provide useful information beyond the WGS data in the current study. However, we believe our study could make some differences to the community in the following aspects.*

*i). In addition to the maize genome assemblies, the population-level WGS data can provide ~68.50% more sequences to the non-reference pan-Zea sequence pool (Figure 1B);*

*ii). About 37% of the anchored non-reference sequence in the pan-Zea genome were not with maize origins (Figure 1C);*

*iii). About 76.15% of the anchored non-reference sequence in the pan-Zea ge-nome cannot be represented by the NAM founder genomes (Figure 1D, updated ac-cording to the reviewer’s minor comments);*

*iv). The 507 maize subgroup of the pan-Zea population used in the current study are broadly used as association mapping panel. Hundreds of agronomic and metabo-lomic traits, and tens of thousands of population-level transcriptomic and epigenetic data have been accumulated based on this panel. These are perfect resources to study the impact of SVs from the multi-omics points of view. Thus, we believe genotyping SVs in the panel is of certain value to the community.*

*Admittedly, it is not as easy to genotype SVs based on WGS data as the SNPs, but it is generally practicable (Alkan et al., 2011; Ho et al., 2020). With the combina-tion of several SV calling methods, SV genotype matrix with acceptable accuracy could be acquired (Sudmant et al., 2015). And with the application of the string graph related algorithms, the accuracy of WGS-based INSertion calls have been remarkably improved (Sibbesen et al., 2018). With the still-high cost of the third generator HIFI sequencing, and the further application of string graph to improve the short reads mapping and variant calling in pan-genomes (Hickey et al., 2020; Siren et al., 2021), we believe the WGS-based SV genotyping would not leave stage in the near-term. In a previous study, we have tested the feasibility of improving the genotyping of INS with WGS data (Yang et al., 2019). And in the current study, we have combined sev-eral evidences to ensure the quality of SV genotyping (as illustrated in details in Sup-plemental Figure S12 and Section 6 of Supplemental Materials and Methods) compe-tent for downstream analyses, as illustrate in response to the reviewer’s previous comment.*

*We appreciate for the reviewer’s insightful suggestions of “adding several high quality assemblies of wild maize”. However, the high heterozygosity and complexity of the teosinte genomes makes this task time and cost consuming, and is beyond the contents of the current project. In fact, in another project, we do have assembled a draft genome assembly of a teosinte (Zea mays ssp. parviglumis) based on more than 90x PacBio HiFi reads (unpublished). In order to check if the lacking of genomes of maize wild relatives could bring biases to the results in the current study (for example, the differences between maize and teosinte specific genes), we have mapped the teo-sinte-core genes and teosinte-lost genes identified in the current study to the Z. par-viglumis genome. We found that 95.24% (29,864/31,355) of the WGS-based teosin-te-core genes had hits in the Z. parviglumis genome, while 98.23% (3,999/4,071) of the WGS-based teosinte-lost genes cannot be mapped to the Z. parviglumis genome. This result is comparable with the maize gPAV accuracy (as illustrated in Figure S6E of Supplementary Figures), indicated that the differences between maize and teosinte specific genes were less likely to be caused by technical biases. We appreciate if the reviewer would understand that we could not currently make this genome public available, however, we are pleased to share the related information to the reviewer for reproducing the above estimations if necessary.*

3. It is unclear to me whether the samples was collected from global sampling or re-stricted to some regions. The detailed information should be provided for the ~700 resequenced samples, for example, the GIS information. At the same time, the authors didn't offer any explanations why different number of samples were used in different analyses, which made the related parts very hard to follow.

*Response: The detailed pedigree information of the maize association mapping panels was listed in Table S1 of (Yang et al., 2011). The detailed GIS information for the 31 landrace samples was listed in Table S1 of (Wang et al., 2017), and the GIS infor-mation for the teosintes can be found in the recently publication (Chen et al., 2021). We have updated the section 1 of Supplementary Materials and Methods to add an explicit statement to refer to these researches for the detailed GIS information. A copy of the detailed information for these samples has also been available for the re-viewer at https://github.com/songtaogui/panz-paper-data/blob/master/07_panz_sample_info/00_PANZ-sample_info.xlsx.*

*There are mainly three subsets of sample sizes in the analyses: i) all the 721 sam-ples were included in the construction of the pan-Zea genome, ii) the 31 landrace in-dividuals were excluded from the gene PAV calling and the downstream analyses, with reasons illustrated in Section 4.1 of Supplementary Materials and Methods “Be-cause of the shorter total length of the draft assemblies and the shorter reads length of the 31 landrace individuals when compared with the maize and teosinte individuals, which may have potential bias, the landrace individuals were not included in the gPAV calling and the downstream analyses”, and iii) only the 507 maize association mapping panel individuals were included in the genetic analyses, in consideration that a) the WGS reads of these 507 samples are PCR-free thus could avoid artificial biases during SV calling and genotyping and b) the multi-omics traits data were only availa-ble in subsets of the 507 samples.*

4. Why only PAVs were analyzed in detail. Many adaptive and phenotypes were as-sociated with INVersions in maize literature. It might be interesting to revisit the clas-sical examples using the new SV genomic map. Such analyses had been conducted in tomato (https://www.sciencedirect.com/science/article/pii/S0092867420306164). At the same time, some of the analyses might be problematic, for example, what is an OPAV? Do the authors mean derived PAVs? Does it overlap with gPAV? Please use standard terms instead.

*Response: In the current study, there are mainly two advantages of leveraging the pan-Zea genomic resources in enhancing maize improvement that we would like to share to the community: i) using pan-Zea sequences and pan-Zea genes can find the causations that could barely reached by using single reference genome, ii) leveraging SVs and the multi-omics traits in maize genetic mapping could help identifying the molecular cau-sations of complex agronomic phenotypes.*

*The two detailed cases were chosen mainly in consideration of their fitness in highlighting the above advantages of leveraging our pan-Zea genome. In the case of the ga1 locus, by leveraging the pan-Zea genome, the causal effect of the PAV of a reference-genome-prematured gene to maize unilateral cross-incompatibility could be directly identified, and the PAV of a serial of genes in the same family with the target gene were found in association with the phenotype, and all showed distinct PAV pat-tern differences among the teosinte subgroup and the modern maize subgroup, which is in agreement with our conclusion on the selection of gene PAV during maize do-mestication. In the other case of the IDP103 locus, we found a TE-related INS in a SV-specific eQTL, could be the causation of the suppression of the tissue specific expression of a gene by breaking the ABRE TF-binding site, which is also related to the variations in response to the drought tolerance.*

*We agree with the reviewer that using SV genomic map would bring new sight to classical cases, including the INV-mediated ones. In fact, both the two cases in the study still need deep mining to ultimately reveal the mechanisms. However, as men-tioned by Reviewer 1, the current study has high volume of information. Thus we de-cided to only keep the cases that could bring intuitive impression on the advantages of the pan-Zea genomic resources to the community. We appreciate for the reviewer’s insightful suggestions on re-visiting classical cases to uncover the INV-mediated phe-notype variation. We would work on these issues as suggested by the reviewer in the next studies and also hope the community will work on them since all the data are public available.*

*In response to the reviewer’s concern of “what is an OPAV”, it referred to “pres-ence and absence of orthologous group”. We have had it defined in Line 116-118 of the main manuscript (please also refer to Section 4.1 of Supplementary Materials and Methods for the identification of oPAVs).*

4. The figures in the pdf file is barely readable to me, please use high-resolution fig-ures

*Response: We apologize for that inconvenience. We have provided vector graph for the main figures in the revised manuscript. In case the reviewer may failed in downloading the source figures we submitted, we have also provided a copy of these figures in Github with accession of: https://github.com/songtaogui/panz-paper-data/tree/master/99_figures.*

Minor Comments

L68: why only 11 were used? There were more than 25 high quality maize assemblies available.

*Response: The 26 NAM founder genomes were not released until 2020, our pan-Zea genome was already constructed at that time. Three reasons were considered when deciding how to leverage the new 26 high-quality maize genomes: i) the construction of the pan genome required several rounds of whole genome alignment and step-wise reads map-ping for anchoring, it is quite time-consuming to reconstruct the pan-genome with the additional 26 NAM founder genomes, ii) we need a standard dataset to estimate the representativeness of our pan-Zea genome, and iii) the additional reference genomes were all modern maize genomes, which may not add much teosinte information to the pan-Zea genome. Taking all these reason, we have decided to separately construct a “pan-NAM non-reference genome” rather than add them to the pan-Zea genome. As illustrated in the previous responses, the pan-Zea genome were capable of representing the information that NAM non-reference genomes would add.*

L69: Please describe the design of the sampling.

*Response: To construct a pan genome that including as much the genomic information as possible, we need the samples to have general representation for all the species in the Zea genus. We collected 183 teosinte accessions encompassing all the species and sub specific taxa in the genus Zea (Chen et al., 2021), a public available dataset of 31 maize landraces spanning the pre-Columbian distribution of maize (Wang et al., 2017), and a widely used association mapping panel of modern maize representing those grown in both temperate and tropical regions (Yang et al., 2011). This sampling as-sured the representation of both the speciation of different Zea species and the diver-sities during maize domestication and adaptation.*

L90-92: Please check the numbers here.

*Response: We appreciate for the reviewer’s perceptive comment. After a thorough check, we found that there were redundant that we should have filtered out in the NAM non-reference sequences, which made the statistics of the NAM non-reference se-quence inaccurate. We have now revised the statistics of the alignment between pan-Zea non-reference sequences and NAM non-reference sequences in both the main manuscript and the Supplementary Test S1 (the detailed alignment result is accessible through https://github.com/songtaogui/panz-paper-data/blob/master/00_vs_nam_nrseq/Filter_xcov80_pid80.tsv.gz), the updated proportion of NAM non-reference sequence that could be mapped with pan-Zea non-reference sequences is now ~94.16% (1,372,909,821 bp / 1,458,111,539 bp). This is a mistake we would be hard to detect if it weren’t for the reviewer’s comment.*

L119: "7% were dispensable", this is very low number, I suspect the low frequency or sample specific gPAVs were under estimated in this study.

*Response: As mentioned in response to the reviewer’s major concern 4, the statement of “7% were dispensable” was referred to ortholog groups. For genes, the proportion of dis-pensable genes was ~44.34% (Line 118). But still, the gPAVs were underestimate with our WGS-based approaches, when compared with those in (Hufford et al., 2021), which found that ~49.59% of the NAM pan-genes were dispensable.*

L120-122: These are big numbers, does this mean ~1/5 gene content difference be-tween any given pair of maize samples.

*Response: Based on our pan-gene set of 58,944 genes, it is more like ~1/10 gene content differences between two maize samples. We have also performed pairwise gene dif-ference analyses based on the 26 NAM founder reference genomes based pan gene sets (results available at https://github.com/songtaogui/panz-paper-data/tree/master/04_nam_pairwise_diff_pav), and the results showed that the gene difference varies from 13,827 to 21,685, with a mean of 18,316, which is more like a ~1/5 proportion. Taking the differences when dealing with gene redundant (as illustrated in the response to Reviewer1’s first major concern) between the two pan-gene sets into account, we thought the number of gene differences in the current study was conservative, if not underestimated.*

L1132: What about variants with a length of 50?

*Response: We apologize for this typo. The criteria for INDELs should be “<= 50 bp”, and we have revised the manuscript accordingly.*

L156-158: What does the authors mean by "the direction evolution"?

*Response: By “the direction of evolution”, we mean the order of the Zea species according to the divergence time. We have revised the description as “the topology of the spe-cies tree” to avoid misleading.*

L178-207: I suggest the authors to use standard population genetic methods.

*Response: Thank you for the suggestions. The analyses here were in general highly inspired by the similar analyses in the gene PAVs of 3,010 diverse accessions of Asian culti-vated rice (Wang et al., 2018). The main purpose for the analyses was to identify the genes that showed different PAV patterns (frequencies) in two groups (teosintes vs maize), which we think is suitable to test with the hypergeometric distribution based Fisher’s exact test. Besides, standard population genetic methods usually test the dif-ferences of allele frequencies. In the cases of gene PAVs here, the presence or absence of a gene was inferred from the read coverage of exons (Golicz et al., 2015) rather than directly sequence variations. Thus, we think it may not be appropriate to take the PAV of a gene as different alleles in a population. Although not directly, we do have leveraged the standard population genetic methods based results, by joining the un-balanced gene PAV results with the selection signals generate according to SNP-based Fst and XPCLR analyses.*

L216-218: There is an obvious insertion-deletion imbalance here. At the same time, INS and DEL should be relatively defined based on the derived status.

*Response: We do have kept more DELs than INSs in the final SV matrix, which is mainly because that the genotyping of DELs based on WGS data are easier than the geno-typing of INSs. As illustrated before, to ensure a general accuracy for downstream analyses, we would rather filter out the SVs with low supportive evidences than kept them. We apologize for not fully understand the reviewer’s comment of “INS and DEL should be relatively defined based on the derived status”, the INS and DEL were both relative to the reference genome, if that’s what the reviewer’s point.*

L225: This is much lower than in rice (https://doi.org/10.1093/molbev/msaa185). Did the authors annotate the INSertions properly?

*Response: We agree with the reviewer that the TE-related SVs were underestimated in the current study, for reasons of i) both the de novo assembly step and the non-reference sequence calling step can filter out highly repetitive sequences; ii) the classical SV calling algorithms required reads with high map quality, which would also filter out the TE-mediated SVs. To accurately genotype TE variations (Mobile Element Inser-tions, or MEIs), additional alignments of reads to TE-specific reference datasets are usually needed, as the PoPoolationTE2 pipeline does as in the rice research suggested by the reviewer. In the current study, we were intent to focus on the classical SVs. Thus, we only checked for the overlap or sequence similarity of annotated TEs for the classical SVs, rather than add additional analyses on de novo identifying MEIs. We appreciate for the reviewer’s recommendation for this creative study in rice, which is definitely merit for reference in further maize MEI related studies.*

L252: This is interesting and worth further digging.

*Response: Thanks for the reviewer’s comments. We do have plans in digging into the general PVE, multi-genic effect and pleiotropy in the maize genetic mapping in further stud-ies.*

L439-440: Only ~33%? This should be much more if high quality data is used.

*Response: We agree with the reviewer that this proportion would be underestimated since the unavailable of high quality teosinte genomes, as one of the limitations we have discussed in the Conclusions section. The whole community is eager for high quality teosinte reference genomes, a goal we and our colleagues are trying to achieve in fur-ther studies.*

*Response References*

*Alkan, C., Coe, B.P., and Eichler, E.E. (2011). Genome structural variation discovery and genotyping. Nature Reviews Genetics 12, 363-376.*

*Chen, L., Luo, J., Jin, M., Yang, N., Liu, X., Peng, Y., Li, W., Qing, L., Yin, Y., Ye, X., et al. (2021). Portrait of a genus: the genetic diversity of Zea. bioRxiv, 2021.2004.2007.438828.*

*Golicz, A.A., Batley, J., and Edwards, D. (2016a). Towards plant pangenomics. Plant Biotechnology Journal 14, 1099-1105.*

*Golicz, A.A., Bayer, P.E., Barker, G.C., Edger, P.P., Kim, H., Martinez, P.A., Chan, C.K.K., Severn-Ellis, A., McCombie, W.R., and Parkin, I.A. (2016b). The pangenome of an agronomically important crop plant Brassica oleracea. Nature communications 7, 1-8.*

*Golicz, A.A., Martinez, P.A., Zander, M., Patel, D.A., Van De Wouw, A.P., Visendi, P., Fitzgerald, T.L., Edwards, D., and Batley, J. (2015). Gene loss in the fungal canola pathogen Leptosphaeria maculans. Functional & integrative genomics 15, 189-196.*

*Hübner, S., Bercovich, N., Todesco, M., Mandel, J.R., Odenheimer, J., Ziegler, E., Lee, J.S., Baute, G.J., Owens, G.L., and Grassa, C.J. (2019). Sunflower pan-genome analysis shows that hybridization altered gene content and disease resistance. Nature plants 5, 54-62.*

*Hickey, G., Heller, D., Monlong, J., Sibbesen, J.A., Siren, J., Eizenga, J., Dawson, E.T., Garrison, E., Novak, A.M., and Paten, B. (2020). Genotyping structural variants in pangenome graphs using the vg toolkit. Genome Biol 21, 35.*

*Ho, S.S., Urban, A.E., and Mills, R.E. (2020). Structural variation in the sequencing era. Nature Reviews Genetics 21, 171-189.*

*Hufford, M.B., Seetharam, A.S., Woodhouse, M.R., Chougule, K.M., Ou, S., Liu, J., Ricci, W.A., Guo, T., Olson, A., Qiu, Y., et al. (2021). De novo assembly, annotation, and comparative analysis of 26 diverse maize genomes. Science 373, 655-662.*

*Kou, Y., Liao, Y., Toivainen, T., Lv, Y., Tian, X., Emerson, J., Gaut, B.S., and Zhou, Y. (2020). Evolutionary genomics of structural variation in Asian rice (Oryza sativa) domestication. Molecular biology and evolution 37, 3507-3524.*

*Li, H., Feng, X., and Chu, C. (2020). The design and construction of reference pangenome graphs with minigraph. Genome biology 21, 1-19.*

*Liu, Y., Du, H., Li, P., Shen, Y., Peng, H., Liu, S., Zhou, G.A., Zhang, H., Liu, Z., Shi, M., et al. (2020). Pan-Genome of Wild and Cultivated Soybeans. Cell 182, 162-176 e113.*

*Montenegro, J.D., Golicz, A.A., Bayer, P.E., Hurgobin, B., Lee, H., Chan, C.K.K., Visendi, P., Lai, K., Doležel, J., and Batley, J. (2017). The pangenome of hexaploid bread wheat. The Plant Journal 90, 1007-1013.*

*Sherman, R.M., Forman, J., Antonescu, V., Puiu, D., Daya, M., Rafaels, N., Boorgula, M.P., Chavan, S., Vergara, C., Ortega, V.E., et al. (2019). Assembly of a pan-genome from deep sequencing of 910 humans of African descent. Nature genetics 51, 30-35.*

*Sibbesen, J.A., Maretty, L., Krogh, A., and The Danish Pan-Genome, C. (2018). Accurate genotyping across variant classes and lengths using variant graphs. Nature Genetics 50, 1054-1059.*

*Siren, J., Monlong, J., Chang, X., Novak, A.M., Eizenga, J.M., Markello, C., Sibbesen, J.A., Hickey, G., Chang, P.C., Carroll, A., et al. (2021). Pangenomics enables genotyping of known structural variants in 5202 diverse genomes. Science 374, abg8871.*

*Stuart, T., Eichten, S.R., Cahn, J., Karpievitch, Y.V., Borevitz, J.O., and Lister, R. (2016). Population scale mapping of transposable element diversity reveals links to gene regulation and epigenomic variation. elife 5, e20777.*

*Sudmant, P.H., Rausch, T., Gardner, E.J., Handsaker, R.E., Abyzov, A., Huddleston, J., Zhang, Y., Ye, K., Jun, G., Hsi-Yang Fritz, M., et al. (2015). An integrated map of structural variation in 2,504 human genomes. Nature 526, 75-81.*

*Wang, L., Beissinger, T.M., Lorant, A., Ross-Ibarra, C., Ross-Ibarra, J., and Hufford, M.B. (2017). The interplay of demography and selection during maize domestication and expansion. Genome biology 18, 1-13.*

*Wang, W., Mauleon, R., Hu, Z., Chebotarov, D., Tai, S., Wu, Z., Li, M., Zheng, T., Fuentes, R.R., Zhang, F., et al. (2018). Genomic variation in 3,010 diverse accessions of Asian cultivated rice. Nature 557, 43-49.*

*Yang, N., Liu, J., Gao, Q., Gui, S., Chen, L., Yang, L., Huang, J., Deng, T., Luo, J., He, L., et al. (2019). Genome assembly of a tropical maize inbred line provides insights into structural variation and crop improvement. Nature genetics 51, 1052-1059.*

*Yang, X., Gao, S., Xu, S., Zhang, Z., Prasanna, B.M., Li, L., Li, J., and Yan, J. (2011). Characterization of a global germplasm collection and its potential utilization for analysis of complex quantitative traits in maize. Molecular Breeding 28, 511-526.*

*Zhou, Y., Minio, A., Massonnet, M., Solares, E., Lv, Y., Beridze, T., Cantu, D., and Gaut, B.S. (2019). The population genetics of structural variants in grapevine domestication. Nature plants 5, 965-979.*

**Second round of review**

**Reviewer 1**

Many thanks for addressing all of my comments so thoroughly. I look forward to seeing your manuscript published.

**Reviewer 2**

Congratulations to the authors for this great achievement! All my concerns had been addressed and I don't have further comments.
